# Investigations for Bronchiolitis in Infants: An Overview of Reviews and Systematic Review of Primary Studies

**DOI:** 10.1002/ppul.71582

**Published:** 2026-03-30

**Authors:** Kate Loveys, Meredith L. Borland, Ed Oakley, Franz E. Babl, Elizabeth Cotterell, Georgia Harrison, Libby Haskell, Sharon O'Brien, Emma J. Tavender, Catherine L. Wilson, Stuart R. Dalziel, Paediatric Research in Emergency Departments International Collaborative (PREDICT) network

**Affiliations:** ^1^ Department of Paediatrics: Child and Youth Health The University of Auckland Auckland New Zealand; ^2^ Department of Emergency Medicine Perth Children's Hospital Perth Western Australia Australia; ^3^ Divisions of Emergency Medicine and Paediatrics The University of Western Australia Perth Western Australia Australia; ^4^ Institute for Paediatric Perioperative Excellence The University of Western Australia Perth Western Australia Australia; ^5^ Departments of Paediatrics and Critical Care The University of Melbourne Parkville Victoria Australia; ^6^ Emergency Department Royal Children's Hospital Parkville Victoria Australia; ^7^ Clinical Sciences Murdoch Children's Research Institute Parkville Victoria Australia; ^8^ Armidale Rural Referral Hospital Armidale New South Wales Australia; ^9^ School of Rural Medicine and Tablelands Clinical School University of New England Armidale New South Wales Australia; ^10^ Peterborough City Hospital Peterborough UK; ^11^ Children's Emergency Department Starship Children's Hospital Auckland New Zealand; ^12^ Australian Catholic University Sydney New South Wales Australia; ^13^ Department of Surgery, School of Medicine The University of Auckland Auckland New Zealand

**Keywords:** bronchiolitis, chest x‐ray, laboratory testing, systematic review, viral testing

## Abstract

**Aim:**

To synthesize evidence on the clinical utility of chest X‐ray (CXR), laboratory and viral testing in infants with bronchiolitis, including in subgroups with unexpected deterioration or intensive care unit (ICU) admission (severe disease).

**Methods:**

An overview of reviews and systematic review of primary studies were conducted. MEDLINE, EMBASE, PubMed, Cochrane Library, CINAHL were searched (2000 to 19/02/25) for systematic reviews and primary studies evaluating investigations in bronchiolitis management at hospital. Risk of bias (ROBIS, NOS) and certainty of evidence (GRADE) were evaluated. Results were narratively synthesized.

**Results:**

Thirty/28,602 publications were included (*N* = 23,605 infants, *N* = 59 studies; three systematic reviews [32 studies], 27 observational studies). In typical bronchiolitis: (1) CXR demonstrated insufficient diagnostic accuracy, increased antibiotic prescriptions, and was not associated with ICU length of stay (LOS) (very low quality of evidence); (2) Laboratory test results were not associated with mortality, and were inconsistent for LOS (very low quality of evidence); (3) Viral testing results were not associated with ICU admission, and were inconsistent for hospitalization (very low quality of evidence). In infants with severe disease: serum procalcitonin and c‐reactive protein testing at ICU admission had some benefit in predicting bacterial co‐infection and/or pneumonia (very low quality evidence). Evidence was lacking for infants with unexpected deterioration.

**Conclusions:**

Very low certainty evidence indicates that CXR, laboratory and viral testing may have limited clinical utility in individual management of typical bronchiolitis, and should not be routinely used in this group. Further research is required in subgroups with unexpected deterioration or severe disease.

**Abbreviations**
AUC: area under the curve
CI: confidence interval
CXR: Chest X‐Ray
GRADE: Grading of Recommendations Assessment, Development and Evaluation
IBI: invasive bacterial infection
ICU: intensive care unit
LOS: length of stay
NA: not applicable
NOS: Newcastle Ottawa Scale for cohort studies
PCR: polymerase chain reaction
RCT: Randomized controlled trial
ROBIS: risk of bias in systematic reviews
RR: Relative risk
RSV: respiratory syncytial virus
RV: rhinovirus
UTI: urinary tract infection
VLCE: very low certainty evidence

## 1 Introduction

Bronchiolitis is the most common reason for infants to be admitted to hospital [1]. It is a seasonal, acute respiratory illness caused by a viral infection, usually respiratory syncytial virus (RSV). Moderate or severe bronchiolitis can involve a combination of hypoxemia, apnea, tachypnoea, feeding difficulties, tachycardia, and irritability or lethargy, and usually requires hospital management. Universally, guidelines recommend that a diagnosis of bronchiolitis is clinical and that treatment is supportive [2, 3, 4, 5, 6, 7, 8, 9, 10].

Despite these universal recommendations, bronchiolitis management has historically been affected by the inappropriate use of low‐value interventions, for which there is insufficient evidence of a clinical benefit to outweigh the risks and costs of use. Chest X‐ray (CXR), laboratory and viral testing are low‐value investigations that are commonly used in bronchiolitis management. These investigations have not been shown to meaningfully change patient management nor result in an improved outcome for infants with typical bronchiolitis receiving emergency department or in‐patient pediatric ward‐based care [11, 12, 13, 14, 15]. CXR do expose infants to radiation [12], and its use has been associated with increased prescriptions of unnecessary antibiotics (by approximately 13%) due to interpretation issues, diagnostic uncertainty and non‐specific radiological findings [15, 16]. For laboratory and viral testing, the sampling process is invasive and often uncomfortable for infants, causing stress for the family [11]. Moreover, all of the above investigations have been associated with significant costs [13, 17], and have been subject to many international “*Choosing Wisely*” campaigns to de‐implement low‐value care [18, 19].

Yet CXR, laboratory, and viral testing continue to be used in the management of typical bronchiolitis. A 2021 cohort study of 602,375 infants with bronchiolitis in the United States (US), reported that approximately 37.3% (95% CI 34.0–40.6), 23.6% (95% CI 18.8–28.3), and 33.5% (95% CI 27.8–39.2) of patients received a CXR, complete blood count, and viral testing for bronchiolitis [20]. Inappropriate use of these investigations has been reported in similar proportions of bronchiolitis patients managed in the United Kingdom, Canada, Australia and New Zealand [15].

Clinician concerns about parental pressure, a lack of experience and confidence in diagnosing and managing bronchiolitis, a perception of a “need to be doing something,” misdiagnosis and illness severity appear to drive the inappropriate use of these investigations in infants with bronchiolitis [12, 13, 15, 21]. While most of these concerns can be ameliorated by education and experience of staff, illness severity of typical bronchiolitis patients and those undergoing unexpected deterioration warrants review of the available evidence. Systematic reviews for typical bronchiolitis patients are not current [11, 12, 13, 22, 23], and to date no systematic review has evaluated the use of investigations in patients undergoing unexpected deterioration or with severe disease. This review aimed to synthesize the evidence evaluating the clinical utility of CXR, laboratory and viral testing in infants (aged < 12 months) presenting or admitted to hospital with bronchiolitis. Secondary aims were to evaluate the clinical utility of CXR and laboratory testing in subgroups with: (1) unexpected deterioration; or (2) severe illness requiring intensive care unit (ICU) or high dependency unit (HDU) care. In this review, clinical utility was an encompassing term that referred to the extent to which an invesitgation influenced important clinical outcomes pertaining to management decisions (e.g., hospital admission, antibiotic prescribing), or patient outcomes (e.g., length of hospital or ICU stay, ICU admission, mortality), for a given research question.

## 2 Methods

An overview of systematic reviews and a systematic review of primary studies were conducted. An overview of reviews methodology was chosen as the research questions involved examining the evidence for different investigations in a population from two or more systematic reviews [24]. A supplementary systematic review of primary studies was conducted to synthesize the eligible literature not covered by the systematic review evidence.

This review informed an update to the Australasian Bronchiolitis Guideline [5, 25, 26]; which provides evidence‐based, clinical guidance for the management of infants with bronchiolitis in metropolitan and regional/rural hospitals throughout Australia and Aotearoa New Zealand. The protocol for this review was registered on PROSPERO (CRD42023463917). The Cochrane Handbook guidance on conducting overviews of reviews [24], and systematic reviews of primary studies were followed [27]. The Preferred Reporting Items for Overviews of Reviews (PRIOR), and the 2020 Preferred Reporting Items for Systematic reviews and Meta‐Analyses (PRISMA) and abstract extension were adhered to (Supporting Information S1: Appendix 1) [28, 29]. An amendment to the original protocol is reported in Supporting Information S1: Appendix 2.

### 2.1 Search Strategy and Study Selection

A systematic search was performed by a subject librarian on Ovid MEDLINE, Ovid EMBASE, CINAHL, PubMed, and the Cochrane Library, from 2000 to 19/02/2025 and limited to the English language (Supporting Information S1: Appendix 3). Manual searches were conducted from conference abstracts and bronchiolitis guidelines. The records were screened independently in duplicate in title and abstract and full‐text stages by a team of 11 researchers using Covidence software (Veritas Health Innovation, Melbourne, Australia), with the Cochrane randomized controlled trial (RCT) classifier enabled. Training meetings were held prior to each stage, and inter‐rater agreement was monitored through Covidence. Rating conflicts were resolved through consensus with a third researcher not involved in the initial vote. Subject matter experts in the research team reviewed the included article list to ensure no key studies were missing.

The eligibility criteria for each of the seven PICO questions are reported in Table 1. We included peer‐reviewed journal articles that reported on the association between use of CXR, or any type of laboratory or viral testing, on at least one of our primary or secondary outcomes in infants (aged < 12 months), presenting or admitted to hospital with bronchiolitis. The primary and secondary outcomes varied between research questions (Table 1), and were determined and prioritized through consensus of the guideline development committee of the Australasian Bronchiolitis Guideline [25]. The primary outcome definitions are presented in Table 2. The included articles could be systematic reviews or primary studies. Systematic reviews were defined as literature reviews reporting a systematic search of electronic databases. Primary studies could include prospective or retrospective observational studies, or randomized controlled trials. Some research questions pertaining to CXR and laboratory testing used additional eligibility criteria for the population, such as the presence of unexpected deterioration (RQ2, RQ5), or receipt of ICU or HDU level care (RQ3, RQ6). Unexpected deterioration was defined as an unexpected requirement for an escalation of care, based on broad criteria as determined and reported by the study authors explicitly around deterioration, time of escalation of care, or admission to hospital or ICU. Studies were screened in duplicate for this information during full‐text screening. The evidence for these subgroups was not assessed for viral testing, as viral testing was not judged to be relevant to the management of these subgroups.

**TABLE 1:** Eligibility criteria per review question in PICO format.

Investigation topic	Research question	Population	Comparator	Outcomes	Study design
Chest X‐ray	RQ1. In infants presenting to hospital or hospitalized with bronchiolitis, does performing a CXR, at the time of presentation or admission, beneficially change medical management or clinically relevant end‐points?	Infants (< 12 months of age) presenting to hospital/ hospitalized with bronchiolitis **(key criteria)**	No CXR An alternative investigation No comparator	**Primary:**	Observational RCTs Systematic reviews
				Diagnostic accuracy Indicator for administration of antibiotics	
				**Secondary:**	
				Cost‐effectiveness Readmission to hospital	
	RQ2. In infants who have an unexpected deterioration with bronchiolitis, does performing a CXR beneficially change medical management or clinically relevant end‐points?	**Key criteria plus:** unexpected deterioration during hospitalization^a^	No CXR An alternative investigation No comparator	**Primary:**	Observational RCTs Systematic reviews
				Diagnostic accuracy Length of stay Length of ICU stay	
				**Secondary:**	
				Cost‐effectiveness Indicator for administration of antibiotics	
	RQ3. In infants severely unwell with bronchiolitis (HDU/ ICU level care), does performing a CXR beneficially change medical management or clinically relevant end‐points?	**Key criteria plus:** presence of severe bronchiolitis requiring ICU or HDU level care	No CXR An alternative investigation No comparator	**Primary:**	Observational RCTs Systematic reviews
				Diagnostic accuracy Length of stay Length of ICU stay	
				**Secondary:**	
				Cost‐effectiveness Indicator for administration of antibiotics	
Laboratory tests	RQ4. In infants presenting to hospital or hospitalized with bronchiolitis, does performing laboratory tests (blood and/or urine), at the time of presentation or admission, beneficially change medical management or clinically relevant end points?	Infants (< 12 months of age) presenting to hospital/ hospitalized with bronchiolitis **(key criteria)**	No test An alternative test	**Primary:**	Observational RCTs Systematic reviews
				Length of stay Death	
				**Secondary:**	
				Length of ICU stay Diagnosis of bacterial co‐infection (incl. pneumonia, UTI)	
	RQ5. In infants who have an unexpected deterioration with bronchiolitis, does performing laboratory tests (blood and/or urine) beneficially change medical management or clinically relevant end‐points?	**Key criteria plus:** unexpected deterioration during hospitalization^a^	No test An alternative test	**Primary:**	Observational RCTs Systematic reviews
				Length of stay Death	
				**Secondary:**	
				Length of ICU stay Diagnosis of bacterial co‐infection (incl. pneumonia, UTI)	
	RQ6. In infants severely unwell with bronchiolitis (HDU/ICU level care), does performing laboratory tests (blood and/or urine) beneficially change medical management or clinically relevant end‐points?	**Key criteria plus:** presence of severe bronchiolitis requiring ICU or HDU level care	No test An alternative test	**Primary:**	Observational RCTs Systematic reviews
				Length of stay Death	
				**Secondary:**	
				Length of ICU stay Diagnosis of bacterial co‐infection (incl. pneumonia, UTI)	
Virological tests	RQ7. In infants presenting to hospital or hospitalized with bronchiolitis, does performing virological investigations beneficially change medical management or clinically relevant end‐points?	Infants (< 12 months of age) presenting to hospital/ hospitalized with bronchiolitis **(key criteria)**	No test An alternative investigation No comparator	**Primary:**	Observational RCTs Systematic reviews
				Rate of hospitalization Death Rate of ICU admission	
				**Secondary:**	
				Length of stay Length of ICU stay	

Abbreviations: CXR, chest X‐ray; HDU, high dependency unit; ICU, intensive care unit; RCT, randomized controlled trial; UTI, urinary tract infection.

^a^ Unexpected deterioration was defined as an unexpected requirement for an escalation of care. Gradual development of an oxygen requirement, increased work of breathing, and/or the need for high flow therapy in the first few days of a bronchiolitis illness are not considered “unexpected deterioration.”

**TABLE 2:** Primary outcome definitions.

Topic	Research question	Primary outcome	Definition
Chest X‐ray	1, 2, 3	Diagnostic accuracy	Test validity for detecting severe bronchiolitis, indicated through sensitivity, specificity, positive predictive values, negative predictive values, area under the curve.
	1	Indicator for administration of antibiotics	Number of infants who initiated antibiotics during the study period.
	2, 3	Length of stay	Length of stay in hospital, from admission to actual or judged ready for discharge. Includes periods of ICU admission.
	2, 3	Length of ICU stay	Length of stay in ICU, from ICU admission to ICU discharge.
Laboratory testing	4, 5, 6	Length of stay	Length of stay in hospital, from admission to actual or judged ready for discharge. Includes periods of ICU admission.
	4, 5, 6	Death	Number of infants with mortality during the study period.
Viral testing	7	Rate of hospitalization	Number of infants admitted to hospital during the study period.
	7	Death	Number of infants with mortality during the study period.
	7	Rate of ICU admission	Number of infants admitted to ICU during the study period.

To avoid introducing bias from double counting outcome data where there were multiple systematic reviews presenting overlapping evidence, we included the most recent, comprehensive, high quality review in accordance with Cochrane guidance [24]. The judgements for inclusion and exclusion of overlapping systematic reviews are presented in Supporting Information S1: Appendix 2. Supplementary primary studies were included that were not presented in the systematic reviews.

### 2.2 Data Extraction

Data were extracted by one researcher using a pre‐piloted spreadsheet form developed for the review, with independent review by a second researcher. Data were extracted on characteristics of the study (e.g., publication date, location, setting), population, investigation, comparator, methods (e.g., study design, primary outcome), and results. All results for an outcome domain were extracted, irrespective of the measure. In accordance with Cochrane guidance for overviews of reviews, eligible evidence was extracted from the systematic reviews [24]. Supporting data would have been sought from study authors, although this was not required.

### 2.3 Risk of Bias Assessment

The risk of bias for the systematic reviews and supplementary primary studies were assessed by one researcher, with independent review by a second researcher. The standardized forms were used for each tool [30, 31]. The risk of bias of the systematic reviews was assessed using the Risk Of Bias In Systematic reviews (ROBIS) tool [30]. ROBIS requires a rating of “high,” “unclear,” or “low” risk of bias across domains of relevance, eligibility criteria, study identification and selection, data collection and study appraisal, and synthesis and findings. An overall risk of bias rating of “high,” “unclear,” or “low” is derived for each review. The risk of bias appraisals that each review reported for its primary studies were extracted, irrespective of the tool used. The reviews used a modified Joanna Briggs Institute (JBI) Prevalence Critical Appraisal Tool [32], and a modified Newcastle Ottawa Scale (NOS) for cross‐sectional studies (developed for the review) [31, 33]. One review did not formally assess the risk of bias for the included studies [34], however reported on issues pertaining to study quality alongside study characteristics (e.g., selection bias).

The risk of bias of the primary studies was evaluated using the Newcastle Ottawa Scale (NOS) for Cohort Studies [31]. The NOS evaluates risk of bias in selection of the cohort (four items), comparability of the cohorts (one item), and the outcome measurement (three items). Each item can be awarded one star if the criteria are met, aside from the comparability of cohorts item which can be awarded two stars maximum. Thresholds per category were applied to convert total scores to quality ratings (good, fair, poor), based on prior research [35] (Supporting Information S1: Appendix 2). The ratings per study were tabulated.

### 2.4 Data Synthesis and Analysis

The data were narratively synthesized following the guidance of Popay and colleagues [36], with possible sources of heterogeneity in the findings discussed. An overview reporting map was produced to summarize the findings [37]. Random‐effects meta‐analyses were performed in two of the three included systematic reviews [33, 38]; these were extracted and reported. Cochrane criteria were followed to determine the appropriateness of quantitative syntheses of primary studies [39]. Quantitative syntheses were not appropriate for our pre‐planned comparisons (testing vs. no testing or an alternative test), as the included studies were heterogenous in terms of the investigations and outcomes evaluated.

### 2.5 Certainty of Evidence Assessment

The certainty of the evidence per outcome was assessed using the Grading of Recommendations Assessment, Development, and Evaluation (GRADE) methodology [40]. The evidence per outcome was appraised for risk of bias, inconsistency, indirectness, imprecision, and other factors (e.g., publication bias) to derive an overall rating of “high,” “moderate,” “low,” or “very low” certainty. In accordance with GRADE methodology, all observational evidence was downgraded to low certainty at the outset as RCT evidence was sought. The appraisals were made by one researcher with review by a second researcher, and tabulated using GRADEpro GDT software [41].

## 3 Results

### 3.1 Study Selection and Characteristics

**FIGURE 1:**
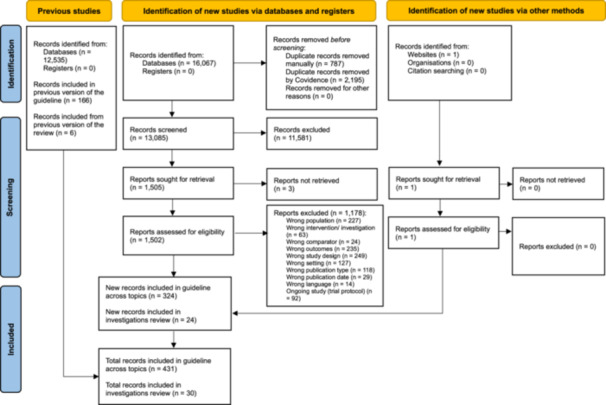
PRISMA flowchart. *Source:* Page MJ, et al. BMJ 2021;372:n71. doi: 10.1136/bmj.n71. This work is licensed under CC BY 4.0. To view a copy of this license, visit https://creativecommons.org/licenses/by/4.0/. [Color figure can be viewed at wileyonlinelibrary.com]

Thirty records were included of 28,602 screened (Figure 1). We included three systematic reviews (covering 32 relevant observational studies) [33, 34, 38], with an additional 15 prospective [42, 43, 44, 45, 46] and 12 retrospective observational studies [47, 48, 49, 50, 51, 52] (*N* = 23,605 infants; *N* = 59 studies overall). There was no primary study overlap across the included systematic reviews. Relevant studies were excluded in mixed populations (e.g., samples with < 75% bronchiolitis or RSV infection) where results were not separately reported [53, 54], with experimental tests not widely available in clinical practice [55, 56], or that reported related but incorrect outcomes (e.g., composite severity measures) [23].

The studies were conducted in a range of countries, including Aotearoa New Zealand (*n* = 1), Argentina (*n* = 1), Australia (*n* = 1), Brazil (*n* = 2), Canada (*n* = 2), Chile (*n* = 2), China (*n* = 3), France (*n* = 2), India (*n* = 2), Israel (*n* = 2), Italy (*n* = 2), Lebanon (*n* = 1), Poland (*n* = 1), Qatar (*n* = 1), Spain (*n* = 5), Turkey (*n* = 3), United Arab Emirates (*n* = 1), United Kingdom (*n* = 1), United States of America (USA) (*n* = 8). One review did not report the location of included studies [38]. The publication dates ranged from 2010 to 2025. The average age of participants ranged from 1.3 to 8 months (where reported). Most studies were conducted in pediatric wards (*n* = 22) and/or ICU settings (n = 18); fewer studies were conducted in EDs (*n* = 8) and outpatient clinics (*n* = 3). The characteristics and results of the individual studies are presented in Supporting Information S1: Appendix 4.

The evidence for CXR consisted of one systematic review reporting on four relevant observational studies [34], and three single‐center retrospective cohort studies across ED, ward, and ICU settings (*N* = 2056) [47, 57, 58]. The evidence covered assessments of atelectasis, hyperinflation, infiltration, peribronchial thickening, perihilar infiltrates, diffuse interstitial markings, pneumonic consolidation and other abnormal findings (e.g., pleural effusion, pneumothorax, foreign body). Two studies from one systematic review compared testing strategies (e.g., CXR/no CXR) [34], four studies compared test results [34, 47, 57], and one study compared both testing strategies and results (CXR/no CXR, positive/negative CXR) [58]. Three studies reported evidence on management decision outcomes (antibiotic prescribing) [34, 57, 58], three studies (two from one systematic review) reported on prognostic or diagnostic value outcomes (ICU LOS, diagnostic accuracy) [34, 47], and one study reported on cost‐effectiveness [34].

The evidence for laboratory testing included one systematic review of 18 relevant observational studies, eight prospective and seven retrospective observational studies (*N* = 13,875) [38, 42, 45, 46, 47, 49, 51, 52, 59, 60, 61, 62, 63, 64, 65, 66]. The laboratory tests included serum procalcitonin (PCT), C‐reactive protein (CRP), monocyte‐to‐lymphocyte ratio, neutrophil‐to‐lymphocyte ratio, white blood cell count (WBC), platelet count (thrombocytosis), systemic immune‐inflammation index, sodium (hyponatremia), glycemia, bacterial cultures (blood, cerebrospinal fluid, tracheal aspirate, urine), serum and urine N‐terminal pro‐brain natriuretic peptide (NT‐proBNP) concentrations, and urinalysis. Most studies (*n* = 33/34) compared test results [38, 45, 46, 47, 49, 51, 52, 59, 60, 61, 62, 63, 64, 65, 66]. One study compared the predictive performance of PCT and CRP results based on the timing of testing relative to ICU admission (e.g., at ICU admission, 24‐h post, 48‐h post) [42]. All studies reported on prognostic or diagnostic value outcomes (LOS, ICU LOS, mortality, diagnosis of bacterial co‐infection).

The evidence on viral testing included one systematic review of 10 relevant observational studies, eight prospective and five retrospective observational studies (*N* = 8271) [33, 43, 44, 47, 48, 50, 60, 61, 67, 68, 69, 70, 71, 72]. The viral testing included polymerase chain reaction (PCR) assays, rapid immunochromatographic assays, direct fluroescent antibody (DFA) kits, and direct immunofluoresence (IF) techniques with nasopharyngeal aspirates. The samples were typically collected within the first 24–48 h of hospital admission. All studies compared test results. The testing aimed to detect the presence or absence of particular viruses (e.g., RSV, rhinovirus), or the degree of viral co‐infection (e.g., one vs. two or more viruses). Three studies within one systematic review reported on management decision outcomes (hospital admission) [33], and 20 studies (seven from one systematic review) reported on prognostic value outcomes (LOS, ICU LOS, ICU admission) [33, 43, 44, 47, 48, 50, 60, 61, 67, 68, 69, 70, 71, 72].

### 3.2 Risk of Bias

The systematic reviews were found to be at high (*n* = 2) and unclear (*n* = 1) risk of bias (Table 3). The reviews were downgraded due to concerns in all domains. Across the reviews, the domains at highest risk of bias were relevance, synthesis and findings. The reviews rated their primary studies as being at moderate to high [33], and moderate to low risk of bias [38]. The reasons for downgrades were not clearly reported.

**TABLE 3:** Assessment of the Risk Of Bias In Systematic reviews (ROBIS).

Review	Phase 1	Phase 2				Phase 3
	Relevance^a^	Study eligibility criteria	Identification and selection of studies	Data collection and study appraisal	Synthesis and findings	Overall risk of bias in the review
Ambrozej 2024	NO	UNCLEAR	LOW	UNCLEAR	HIGH	HIGH
McDaniel 2019	NO	UNCLEAR	LOW	LOW	UNCLEAR	UNCLEAR
Williams 2012	UNCLEAR	UNCLEAR	UNCLEAR	HIGH	HIGH	HIGH

^a^ Does the research question addressed by the review match the target question?

The 27 primary studies were mostly rated at poor quality (*n* = 21), with fewer judged to have fair (*n* = 5) or good quality (*n* = 1) (Table 4). All studies were downgraded due to the risk of selection bias (*n* = 27), and most were downgraded for the comparability of cohorts (*n* = 20). Fewer studies (*n* = 6) were downgraded for bias related to the outcome assessment.

**TABLE 4:** Assessment of risk of bias using the Newcastle Ottawa Scale for cohort studies [31].

Study ID	Selection domain				Comparability domain	Outcome domain			Total score^c^	Rating
	Representativeness of the exposed cohort^a^	Selection of the non‐exposed cohort^a^	Ascertainment of exposure^a^	Demonstration the outcome of interest not present at study start^a^	Comparability of cohorts on basis of design or analysis^b^	Outcome assessment^a^	Adequate length of follow‐up^a^	Adequacy of follow‐up of cohorts^a^		
Akande et al. 2024	—	—	*	*	*	*	*	*	6	Fair
Al Shibli et al. 2017	*	—	*	*	—	*	*	—	5	Poor
Alejandre et al. 2021	*	—	*	*	—	*	*	*	6	Poor
Bamberger et al. 2012	*	—	*	*	—	*	*	*	6	Poor
Bermúdez‐Barrezueta et al. 2023	—	—	*	*	**	*	*	*	7	Fair
Boggio et al. 2023	—	—	*	*	—	*	*	*	5	Poor
Burrack et al. 2023	—	—	*	*	*	*	*	*	6	Fair
Cebey‐Lopez et al. 2016	—	—	*	*	—	*	*	*	5	Poor
Celik et al. 2020	*	—	*	*	—	*	*	—	5	Poor
Coleman et al. 2019	—	—	*	*	—	*	*	*	5	Poor
Erdede et al. 2023	*	—	*	*	—	*	*	*	6	Poor
Fares et al. 2011	*	—	*	—	—	*	*	*	5	Poor
Gaurav et al. 2024	—	—	*	*	—	—	—	*	3	Poor
Huguenin et al. 2012	—	—	*	*	*	*	*	*	6	Fair
Laham et al. 2014	*	—	*	*	—	*	*	*	6	Poor
Mansbach et al. 2012	—	—	*	—	**	*	*	*	6	Poor
Matera et al. 2022	*	—	*	*	—	*	*	*	6	Poor
Mitri et al. 2012	*	—	*	*	*	*	*	*	7	Good
Petrarca et al. 2018	*	—	*	*	—	*	*	—	5	Poor
Rodriguez‐Gonzalez et al. 2023	—	—	*	*	*	*	*	*	6	Fair
Rodriguez‐Gonzalez et al. 2022	*	—	*	*	—	*	*	*	6	Poor
Sai Kotha et al. 2023	—	—	*	*	—	*	*	*	5	Poor
Sun et al. 2020	—	—	*	*	—	*	*	*	5	Poor
Tan et al. 2021	—	—	*	—	—	*	*	*	4	Poor
Towriss et al. 2025	—	—	*	*	—	*	*	—	4	Poor
Yu et al. 2010	—	—	*	*	—	*	*	*	5	Poor
Wrotek et al. 2019	*	—	*	*	—	*	*	—	5	Poor

^a^ Maximum 1 star score.

^b^ Maximum 2 star score.

^c^ Maximum total score 9 stars.

### 3.3 Review Findings

A summary of the findings and certainty of the evidence per investigation and outcome are presented in Table 5.

**TABLE 5:** Summary of findings and certainty of evidence across investigations and population subgroups.

Outcome	Investigation						
	Chest X‐ray			Laboratory testing			Viral testing
	Typical bronchiolitis	Unexpected deterioration	ICU level care	Typical bronchiolitis	Unexpected deterioration	ICU level care	Mixed^b^
Diagnostic accuracy	⊕⊖⊖⊖ Very low^a^ x	—	—	NA	NA	NA	NA
Indicator for administration of antibiotics	⊕⊖⊖⊖ Very low^a^ +	—	—	NA	NA	NA	NA
Length of stay	NA	—	—	⊕⊖⊖⊖ Very low^a^ ±	—	—	⊕⊖⊖⊖ Very low ±
Length of ICU stay	NA	—	⊕⊖⊖⊖ Very low x	—	—	⊕⊖⊖⊖ Very low ±	⊕⊖⊖⊖ Very low x
Death	NA	NA	NA	⊕⊖⊖⊖ Very low^a^ x	—	⊕⊖⊖⊖ Very low^a^ x	—
Rate of hospitalization	NA	NA	NA	NA	NA	NA	⊕⊖⊖⊖ Very low^a^ ±
Rate of ICU admission	NA	NA	NA	NA	NA	NA	⊕⊖⊖⊖ Very low^a^ x
Cost‐effectiveness	⊕⊖⊖⊖ Very low ‐	—		NA	NA	NA	NA
Readmission to hospital	—	NA	NA	NA	NA	NA	NA
Diagnosis of bacterial co‐infection (incl. pneumonia, UTI)	NA	NA	NA	⊕⊖⊖⊖ Very low ?	—	⊕⊖⊖⊖ Very low +	NA

*Note*: — Indicates the outcome was not evaluated for the investigation and population. Findings classification (where evidence is available): **±**: inconsistent findings; **x**: no significant association (*p* > 0.05); **+**: significant increase or positive association (*p* < 0.05); **‐**: significant decrease or negative association (*p* < 0.05);?: inconclusive.

Abbreviations: ICU, intensive care unit; NA, not applicable; UTI, urinary tract infection.

^a^ Primary outcome for the topic.

^b^ Includes patients with typical bronchiolitis and more severe illness requiring ICU level care.

### 3.4 Chest X‐Ray (CXR)

There was limited evidence on the use of CXR in infants with typical bronchiolitis managed outside of the ICU, and no evidence in infants with unexpected deterioration. Two observational studies within a systematic review found that atelectasis, hyperinflation, infiltration, and a composite score of these were not significantly associated with illness severity in a mixed sample of infants with bronchiolitis, including within the ICU setting (very low certainty evidence [VLCE]) [34]. Performing a CXR was associated with a significant increase in prescriptions of antibiotic medication to infants with typical bronchiolitis (VLCE) [34, 57, 58]. In one study, 39.9% of infants with a benign CXR were prescribed antibiotic medication [57]. Omitting CXR was associated with a cost saving of CAD$59.09 per patient (based on 2005 cost evidence; USD$42.82) (VLCE) [34]. Abnormal CXR findings were not significantly associated with ICU length of stay (LOS) in a small retrospective study of ICU patients (VLCE) [47].

### 3.5 Laboratory Testing

One systematic review of 18 observational studies and 11 additional observational studies reported on the association between laboratory test results and clinical outcomes for infants with typical bronchiolitis [38, 45, 49, 52, 59, 60, 61, 62, 63, 64, 65, 66]. The systematic review reported on the use of urinalysis testing to diagnose urinary tract infections (UTI) in infants hospitalized with bronchiolitis (VLCE) [38]. The review found that cut‐offs for a positive urinalysis result varied between studies, however the most common was ≤ 10,000 cfu/mL of a single pathogen on a catheterized specimen. When a positive urinalysis test result was required for a diagnosis of UTI, the estimated prevalance of UTI in infants with bronchiolitis decreased from 3.1% (95% CI 1.8%–4.6%) to 0.8% (95% CI 0.3%–1.4%), indicating an uncommon prevalence in patients with bronchiolitis. Neither a positive tracheal aspirate culture, monocyte‐to‐lymphocyte ratio, nor neutrophil‐to‐lymphocyte ratio were significantly associated with diagnoses of bacterial co‐infection in two studies (VLCE) [49, 62], aside from one finding where infants within the highest quartile of neutrophil‐to‐lymphocyte ratio results were found to have a greater incidence of pneumonia (9.6% overall) [49].

The evidence was inconsistent as to whether laboratory test results were predictive of hospital length of stay in infants with typical bronchiolitis, with findings varying by test. The presence of bacteremia was associated with a longer length of hospital stay (mean 4.6 days difference) [45], as were lymphocyte count, urinary log‐10‐NT‐proBNP/creatinine ratio, CRP, CRP/albumin, glycaemia, and NT‐proBNP levels [63, 65, 66]. However, each of these tests were evaluated across one to two small observational studies that may have been susceptible to selection bias (VLCE). A neutrophil‐to‐lymphocyte ratio cut‐off of > 1.04 was predictive of prolonged hospitalization ( ≥ 9 days) in one study, albeit with a degree of accuracy slightly above chance level (Area under the curve (AUC) 0.68 (95% CI 0.55–0.80) [60]. The evidence for monocyte‐to‐lymphocyte and neutrophil‐to‐lymphocyte ratios were otherwise mixed [49]. The presence of thrombocytosis, a positive tracheal aspirate culture, systemic immune inflammation index, and eosinophil values were not significantly associated with length of hospital stay [52, 59, 61, 62, 64]. None of the evaluated tests were significantly associated with incidence of death in this population (urinary log‐10‐NT‐proBNP/creatinine ratio, CRP, NT‐proBNP, glycaemia, thrombocytosis) (VLCE) [59, 65, 66]. Overall, there were no clear biomarkers reliably associated with the clinical outcomes evaluated in infants with typical bronchiolitis.

Eight observational studies evaluated the use of laboratory testing in infants with severe bronchiolitis receiving ICU care [42, 45, 46, 47, 49, 51, 52, 66]. The evidence from two observational studies indicated that elevated serum PCT could play a role in the diagnosis of bacterial co‐infection in infants with severe bronchiolitis receiving ICU care. In one study, increased serum PCT (> 1.5 ng/mL cut‐off) was significantly associated with a diagnosis of bacterial co‐infection (AUC 0.88, Sensitivity (Se) 0.80, Specificity (Sp) 1.00, *p* < 0.0001), whereas reduced WBC (< 6400/µL cut‐off value) was not [51]. In a second study, elevated serum PCT at ICU admission (> 1.4 ng/mL cut‐off) was shown to have good predictive accuracy for a diagnosis of invasive bacterial infection (IBI) (AUC 0.84 (95% CI 0.79–0.88)), sepsis (AUC 0.91 (95% CI 0.87–0.95)), and pneumonia (AUC 0.82 (95% CI 0.77–0.87)) (VLCE) [42]. However, after 48 h of ICU admission, the accuracy declined to just above chance level for IBI and pneumonia. Elevated CRP (26 mg/dL cut‐off) at ICU admission was shown to have moderate accuracy at predicting diagnoses of IBI (AUC 0.72 (95% CI 0.66–0.76)), sepsis (AUC 0.73 (95% CI 0.66–0.80)), and pneumonia (AUC 0.77 (95% CI 0.72–0.83)), which declined to just above chance level after 48 h. A third study of 28 infants with severe bronchiolitis in an ICU reported that of the seven infants with a positive PCR, only one had a positive blood bacterial PCR (VLCE) [45].

Other forms of laboratory testing did not appear to be of value in the ICU subgroup, based on limited evidence. There was no significant difference in the incidence of death based on the presence of hyponatremia in infants with severe bronchiolitis in one small ICU study (*n* = 60) (VLCE) [46], nor was there a difference based on glycaemia, CRP, CRP/albumin, or NT‐proBNP values (*n* = 37) (VLCE) [66]. There was no significant association between the presence of hyponatremia, thrombocytosis, glycaemia, levels of CRP, CRP/albumin, monocyte‐to‐lymphocyte ratios, or neutrophil‐to‐lymphocyte ratios on ICU LOS in infants with severe bronchiolitis (VLCE) [42, 45, 46, 49, 51, 52, 66]. There was a significantly shorter ICU LOS (approximately 1.67 days) associated with co‐detection (a positive respiratory culture and elevated polymorphonuclear neutrophils), detected from an endotracheal tube aspirate, compared to no co‐detection (adjusted relative risk (Adj RR) 0.81 (95% CI 0.69–0.94), *p* < 0.01), however this was based on the results of one small retrospective study (*n* = 148) (VLCE) [47]. There were no eligible studies on the use of laboratory testing in infants with bronchiolitis and unexpected deterioration.

### 3.6 Viral Testing

There were inconsistent findings for the association between viral test results, and rates of hospital admission and LOS in infants with bronchiolitis, based on observational evidence within a systematic review [33]. In three small studies, the rates of hospital admission were numerically higher for infants with bronchiolitis and rhinovirus infection compared to RSV infection, however the statistical significance was only reported in one study (VLCE) [33]. The findings were also inconsistent for the outcome of LOS, based on the results of five observational studies comparing RSV‐ and rhinovirus‐bronchiolitis (VLCE) [33]. Moreover in 11 supplementary observational studies, the findings were inconsistent for the association between the degree of viral co‐infection [48, 50, 61, 67, 70, 71], or the type of viral infection [43, 60, 61, 68, 69, 70, 72], on LOS (VLCE).

The review found no significant difference in the odds of ICU admission between infants with RSV‐ and rhinovirus‐bronchiolitis (OR 0.87 (95% CI 0.01–3.97), *p* = 0.76), based on a meta‐analysis of five observational studies (VLCE) [33]. There was no significant difference in the ICU admission rates between infants with single versus multiple viral infections [48, 67, 70, 71], nor with varying types of viral infection (RSV, rhinovirus, RSV or rhinovirus co‐infections with other viruses) (VLCE) [69, 70, 72]. Most of the evidence indicated no significant association between viral test results and ICU LOS. This included for comparisons of single versus multiple viral infections (VLCE) [48, 50], RSV versus human bocavirus infection (VLCE) [44], and comparisons of RSV only, also RSV, no RSV, no pathogen (VLCE) [43]. The exception was one small retrospective study (*N* = 168) that reported a significantly longer ICU LOS associated with RSV‐bronchiolitis (Adj RR 1.40 (95% CI 1.20–1.63), *p* < 0.001) (VLCE) [47].

### 3.7 Certainty of Evidence

The certainty of the evidence for each investigation and outcome is detailed in Supporting Information S1: Appendix 5 and summarized in Table 3. The certainty of evidence was very low for all outcomes per investigation due to concerns about risk of bias, indirectness, and imprecision.

### 3.8 Recommendations for Clinical Practice

**FIGURE 2:**
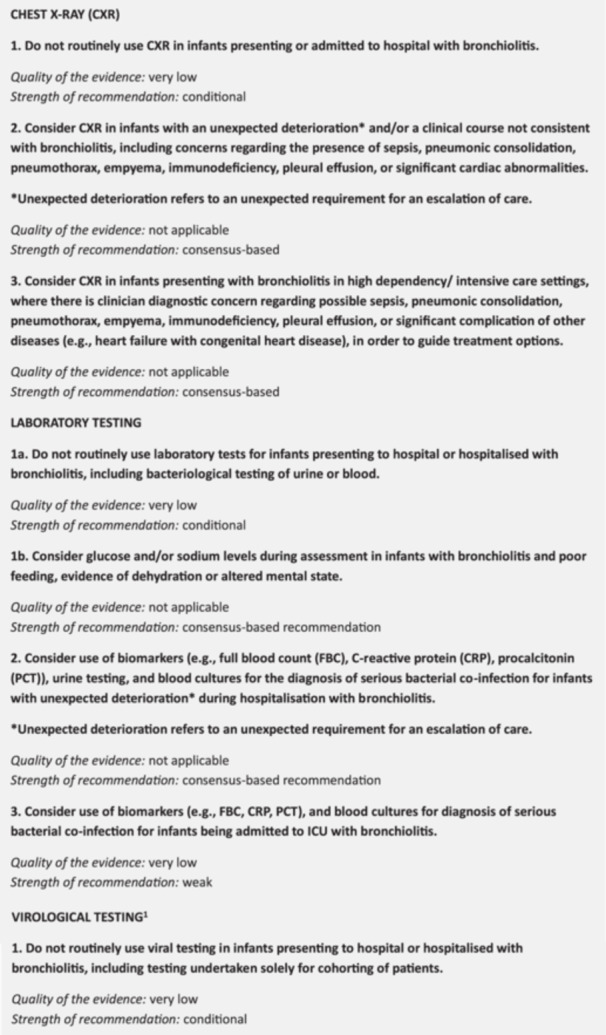
Recommendations for the use of investigations in infants with bronchiolitis [5, 26]. ^1^The recommendation for viral testing refers to testing to inform individual patient management decisions in the context of uncomplicated bronchiolitis. Selective testing may be required for institutional policy, infection‐control or public health contexts, to inform management of treatable viral pathogens (e.g., influenza, SARS‐CoV‐2), or for prognostic enrichment. [Color figure can be viewed at wileyonlinelibrary.com]

A series of clinical practice recommendations were developed from the findings of this review, pertaining to the use of CXR, laboratory and viral testing in infants with typical bronchiolitis, and infants with bronchiolitis and unexpected deterioration or severe illness requiring ICU management (Figure 2). Together, they indicate the following suggestions for clinical practice:

1. For individual patient management of uncomplicated bronchiolitis, do not routinely perform CXR, laboratory testing (including inflammatory markers or bacteriological testing), or viral testing.
  However, consider assessment of glucose and/or sodium levels in infants with uncomplicated bronchiolitis and poor feeding, evidence of dehydration, or altered mental state.
2. For infants requiring HDU/ICU‐level care or with unexpected deterioration (an unexpected requirement for care escalation), consider assessment of inflammatory biomarkers (e.g., CRP, PCT) as adjuncts plus blood cultures and CXR as clinically indicated (see Figure 2). Biomarkers should be interpreted in the context of clinical signs.

## 4 Discussion

Although for the past 60 years the key message for bronchiolitis management has been to safely do less [73], there is evidence that CXR, laboratory, and viral testing continue to be overused in bronchiolitis management [15, 20]. This is the first overview of reviews and systematic review of primary studies to synthesize the evidence on the clinical utility of CXR, laboratory and viral testing in infants with bronchiolitis, including in subgroups for whom there may be a benefit to performing testing, such as infants with unexpected deterioration or severe illness requiring intensive care.

This review found insufficient evidence of a clinical benefit to performing CXR, laboratory, or viral testing as part of the routine, individual management of typical bronchiolitis at hospital. Across the investigations evaluated, the evidence tended to show inconsistent or no associations between test results and important clinical outcomes, such as hospital or ICU LOS, and rates of hospital and ICU admission [33, 43, 44, 46, 47, 48, 49, 50, 52]; albeit the evidence was of very low certainty due to concerns about risk of bias, indirectness, and imprecision. The clinical implication of this finding is that these investigations are unlikely to be of value in the assessment of bronchiolitis severity in hospital, or for informing clinical decision‐making for infants with typical bronchiolitis. This finding is consistent with the last systematic review that evaluated multiple bronchiolitis investigations in 2004, despite the evidence having matured [22]. Our findings are also in keeping with more recent literature reviews and viewpoints discussing the evidence against the use of CXR [12, 16], laboratory [23], and viral testing in the hospital management of typical bronchiolitis [11, 13, 14].

We also looked at the evidence for the clinical utility of CXR and laboratory testing in subgroups for whom these tests may be indicated as part of bronchiolitis management, including infants with unexpected deterioration inconsistent with a usual clinical course of bronchiolitis, and infants with severe illness requiring intensive care. For these subgroups, the results of CXR and laboratory testing may bring clincians greater certainty in the presence of diagnostic concerns (e.g., regarding the presence of sepsis, pneumonic consolidation, pneumothorax, or bacterial co‐infection), and guide subsequent treatment options. Our review did not identify any evidence in infants with an unexpected deterioration, and there was limited, very low certainty evidence in infants with severe bronchiolitis receiving intensive care. In the severe illness subgroup, an assessment of blood biomarkers, such as elevated serum PCT (> 1.4–1.5 ng/mL) and CRP (26 mg/dL cut‐off), at ICU admission was found to be predictive of diagnoses of invasive bacterial co‐infection, including sepsis and pneumonia, with very low certainty evidence [42, 51].

It is important to acknowledge that there are other situations in which CXR, laboratory, and viral testing may play a valuable role in the management of infants in hospital, where testing is undertaken for purposes other than individual bronchiolitis management. These may include performing viral testing for hospital and/or public health monitoring requirements (e.g., during a pandemic environment), or undertaking a CXR in an infant with bronchiolitis and a history of possible congenital heart disease, foreign body aspiration, or chronic respiratory symptoms for purposes related to those conditions. In a seriously ill infant with possible sepsis, where bronchiolitis is a part of a broad differential diagnosis, blood testing may be necessary. Testing performed for purposes other than bronchiolitis management were outside the scope of this review, but play an important role in patient management.

Overall, the clinical practice recommendations from our systematic review (Figure 2) are consistent with other clinical practice guidelines for bronchiolitis in the US, Italy, Egypt, Australia, New Zealand and the United Kingdom [3, 4, 8, 9, 74]. However, these guidelines did not address the critical subgroups of infants with unexpected deterioration and ICU management. The resulting consensus‐based recommendations developed for these subgroups provides clinicians with pragmatic guidance allowing consideration of the investigations in those patients most critically unwell, while preserving the evidence not to use these low‐value investigations in patients with mild and moderate disease (Figure 2). This addresses a key dilemma in both guideline development and clinical practice, where evidence often fails to include the most severely unwell patients.

Theory‐informed, locally‐tailored implementation support should be paired with the release of clinical practice guidelines informing the use of CXR, laboratory and viral testing in bronchiolitis management. Although passive release of a clinical guideline has been shown to improve evidence‐based management of bronchiolitis [20], theory‐informed implementation interventions may improve practice by an additional 14% [75]. Some examples of effective interventions have included assigning local clinical leads for guideline implementation, conducting interest‐holder meetings, hosting train‐the‐trainer workshops, providing educational interventions to clinical staff, conducting monthly audits with staff feedback and comparing the hospital's performance to a top‐performing hospital [75, 76]. These strategies have been shown to be acceptable to clinical staff involved in bronchiolitis management across 13 hospitals [77]. Further strategies may include providing families with standardized materials (e.g., videos, pamphlets) that give informational support and reassurance surrounding a “safely doing less” approach [12]; removing low‐value investigations (e.g., CXR) as selectable options in order sets for bronchiolitis [78, 79]; and delivering digital alerts when low‐value investigations are inappropriately ordered [79]. Implementation interventions may help to reduce inappropriate use of CXR, laboratory and viral testing in bronchiolitis, that is contrary to the clinical evidence.

### 4.1 What This Review Does Not Answer

The scope of this review was investigations (CXR, laboratory or viral testing) to inform individual management of bronchiolitis at hospital, including ED, pediatric ward, and HDU/ICU settings. Our review focused on infants with uncomplicated bronchiolitis and subgroups with unexpected deterioration or requiring HDU/ICU level management. Our review does not look at the evidence for, nor make recommendations on, the use of these investigations performed for other purposes. For viral testing specifically, there are a number of situations where performing selective testing may be warranted. This includes where institutional policy requires testing for treatable viruses (e.g., SARS‐CoV‐2, influenza) with a high community prevalence, to inform epidemiological surveillance, or to inform infection control decisions relating to cohorting or isolation of patients at hospital. It is possible that selective viral testing could be warranted in the future on the basis of emerging evidence surrounding differing clinical phenotypes and prognoses associated with some viruses (e.g., RSV vs. HRV) [33, 80]. However, where respiratory virus testing is used, its value should be evaluated locally to understand impacts on antibiotic use and course duration, isolation practices, and patient flow, as stewardship effects may vary across geographical settings and testing platforms.

### 4.2 Limitations

A key limitation of this review was the indirectness of the body of evidence relative to the research questions. Although the included systematic reviews reported on relevant evidence, they did not evaluate the same research questions as our overview of reviews. This creates a risk that the reviews could have excluded studies that would have been relevant to our research questions. However, our supplementary systematic review of primary studies would have captured any further eligibile studies not included in the systematic reviews. A further issue with the directness of the evidence was found in the systematic review of primary studies, where most of the included studies compared outcomes by groups with varying test results (e.g., co‐infection vs. single infection; RSV positive vs. RSV negative), as opposed to by groups that did or did not receive testing, or alternative tests. For laboratory tests specifically, it is possible for the studies to have included infants with misdiagnosed bronchiolitis (e.g., sepsis). The quality of the evidence was further impaired by the lack of RCTs to understand the effects on clinical management and outcomes. Very few eligible systematic reviews were identified for each topic (CXR = 2, laboratory tests = 3, viral tests = 2; five unique reviews) (Supporting Information S1: Appendix 2), and the reviews within a topic reported overlapping as opposed to complementary evidence. Although we included the most comprehensive and recent, non‐overlapping systematic reviews, two of three were found to be at high risk of bias.

### 4.3 Future Research

Although there is consistent evidence to indicate that CXR, laboratory and viral testing should not have a role in the management of typical bronchiolitis, the findings are based on very low certainty evidence because of the lack of directly relevant, randomized trials. The ethical issues of exposing infants to testing that is anticipated to be unhelpful yet invasive, uncomfortable, and/or associated with radiation exposure suggests that further maturing of the evidence base is unlikely, and perhaps unwarranted. However, the findings of this review indicate several other important areas for future research, such as strengthening the evidence in subgroups where the use of these investigations may be beneficial. Large, multi‐center prospective observational research to further understand the role of biomarkers in diagnosing bacterial co‐infection in infants with bronchiolitis and unexpected deterioration or severe illness requiring intensive care are required. Research is also needed to define the clinical factors present in these subgroups that may result in improved outcomes through performing CXR. In addition, ongoing implementation and de‐implementation research is needed to support evidence‐based practice in regards to CXR, laboratory, and viral testing in bronchiolitis management.

## 5 Conclusion

The evidence supports de‐implementing CXR, laboratory and viral testing from the routine, individual management of uncomplicated bronchiolitis at hospital. These investigations were found to have inconsistent or no associations with the important clinical outcomes that we investigated, indicating that they are unlikely to be of value for informing individual management decisions. Given the very low certainty of the evidence, these conclusions should be interpreted cautiously; nonetheless, they are consistent with existing guideline recommendations to avoid routine testing in typical bronchiolitis. Further research is needed in infants with unexpected deterioration or severe illness requiring intensive care.

## Author Contributions

All authors contributed to the conceptualization of the research, screening of records, and review and editing of the manuscript. Meredith L Borland, Stuart R Dalziel, Kate Loveys, Emma J Tavender developed the methodology. Kate Loveys extracted the data, and performed evidence appraisals with review by Meredith L Borland, Stuart R Dalziel, Ed Oakley. Kate Loveys completed the formal analysis, data visualization, original draft writing, and integrated feedback. Kate Loveys and Catherine L Wilson provided project administration. Meredith L Borland and Stuart R Dalziel provided supervision. Franz E Babl, Meredith L Borland, Stuart R Dalziel, Emma J Tavender secured funding and resources.

## Conflicts of Interest

In accordance with ICMJE criteria, F.E.B., E.C., G.H., L.H., K.L., S.O., E.O., E.J.T., and C.L.W. have no conflicts of interest to declare. M.L.B. and S.R.D. have been provided with investigational product from Amphastar Pharmaceuticals Company (epinephrine inhalers), USA, and Trudell Medical (spacers), Canada, for a study of dexamethasone and epinephrine in bronchiolitis. None of the authors receive any personal financial benefits from industry sponsors. There were no industry sponsors for this review, nor did any of those listed have any role in the review's design, analysis, manuscript drafting, or decision to submit for publication.

## Supporting information


**Appendix 1:** PRIOR and PRISMA 2020 Checklists. **Appendix 2:** Supporting methods. **Appendix 3:** Systematic search strategies. **Appendix 4:** Study characteristics. **Appendix 5:** GRADE certainty of evidence tables.

## Data Availability

Data extraction forms and data can be accessed through request to the corresponding author.
