# Supplementary material for: Investigations for Bronchiolitis in Infants: An Overview of Reviews and Systematic Review of Primary Studies
Source: Pediatr Pulmonol. 2026 Mar 30;61(4):e71582. doi: 10.1002/ppul.71582 (PMC13034406; doi:10.1002/ppul.71582)
Supplement: Supplementary file 1 — Appendix 1: PRIOR and PRISMA 2020 Checklists. Appendix 2: Supporting methods. Appendix 3: Systematic search strategies. Appendix 4: Study characteristics. Appendix 5: GRADE certainty of evidence tables. [file PPUL-61-0-s001.docx]

Loveys K, Borland ML, Oakley E, Babl FE, Cotterell E, Harrison G, Haskell L, O’Brien S, Tavender EJ, Wilson CL, Dalziel SR, on behalf of the Paediatric Research in Emergency Departments International Collaborative (PREDICT) Network. Investigations for bronchiolitis in infants: an overview of reviews and systematic review of primary studies. Pediatric Pulmonology. 2026;e71582. doi:10.1002/ppul.71582.

# Supplementary material

**Contents**

[Appendix 1. PRIOR and PRISMA 2020 Checklists 2](#_Toc204935486)

[Appendix 2. Supplementary methods 13](#_Toc204935487)

[Appendix 3. Systematic search strategies 15](#_Toc204935488)

[Appendix 4. Study characteristics 36](#_Toc204935489)

[Chest Xray 36](#_Toc204935490)

[Laboratory tests 41](#_Toc204935491)

[Virological tests 62](#_Toc204935492)

[Appendix 5. GRADE certainty of evidence tables 79](#_Toc204935493)

## Appendix 1. PRIOR and PRISMA 2020 Checklists

**PRIOR Checklist (1) for overviews of reviews**

| **Section**  **Topic** | **#** | **Item** | **Location reported** |
| --- | --- | --- | --- |
| **TITLE** | | | |
| Title | 1 | Identify the report as an overview of reviews. | Title |
| **ABSTRACT** | | | |
| Abstract | 2 | Provide a comprehensive and accurate summary of the purpose, methods, and results of the overview of reviews. | Abstract (objectives, study design, results sections) |
| **INTRODUCTION** | | | |
| Rationale | 3 | Describe the rationale for conducting the overview of reviews in the context of existing  knowledge. | ‘Introduction’ section, paragraphs 2-4 |
| Objectives | 4 | Provide an explicit statement of the objective(s) or question(s) addressed by the overview of reviews. | ‘Introduction’ section, paragraph 5 |
| **METHODS** | | | |
| Eligibility criteria | 5a | Specify the inclusion and exclusion criteria for the overview of reviews.  If supplemental primary studies were included, this should be stated, with a rationale. | ‘Search strategy and study selection’ section, paragraphs 2-3, table 1; ‘Methods’ section paragraph 1 |
|  | 5b | Specify the definition of ‘systematic review’ as used in the inclusion criteria for the overview of reviews. | ‘Search strategy and study selection’ section, paragraph 2 |
| Information sources | 6 | Specify all databases, registers, websites, organizations, reference lists, and other sources searched or consulted to identify systematic reviews and supplemental primary studies (if included). Specify the date when each source was last searched or consulted. | ‘Search strategy and study selection’ section, paragraph 1 |
| Search strategy | 7 | Present the full search strategies for all databases, registers and websites, such that they could be reproduced. Describe any search filters and limits applied. | ‘Appendix 3. Systematic search strategies’ |
| Selection process | 8a | Describe the methods used to decide whether a systematic review or supplemental primary study (if included) met the inclusion criteria of the overview of reviews. | ‘Search strategy and study selection’ section, paragraph 1 |
|  | 8b | Describe how overlap in the populations, interventions, comparators, and/or outcomes of systematic reviews was identified and managed during study selection. | ‘Search strategy and study selection’ section, paragraph 3 |
| Data collection process | 9a | Describe the methods used to collect data from reports. | ‘Data extraction’ section, paragraph 1 |
|  | 9b | If applicable, describe the methods used to identify and manage primary study overlap at the level of the comparison and outcome during data collection. For each outcome, specify the method used to illustrate and/or quantify the degree of primary study overlap across systematic reviews. | Not applicable (see paragraph 3 of ‘search strategy and study selection’ section) |
|  | 9c | If applicable, specify the methods used to manage discrepant data across systematic reviews during data collection. | Not applicable |
| Data items | 10 | List and define all variables and outcomes for which data were sought. Describe any assumptions made and/or measures taken to identify and clarify missing or unclear information. | ‘Data extraction’ section, paragraph 1 |
| Risk of bias assessment | 11a | Describe the methods used to assess risk of bias or methodological quality of the included systematic reviews. | ‘Risk of bias assessment’ section, paragraph 1 |
|  | 11b | Describe the methods used to collect data on (from the systematic reviews) and/or assess the risk of bias of the primary studies included in the systematic reviews. Provide a justification for instances where flawed, incomplete, or missing assessments are identified but not re-assessed. | ‘Risk of bias assessment’ section, paragraph 1 |
|  | 11c | Describe the methods used to assess the risk of bias of supplemental primary studies (if included). | ‘Risk of bias assessment’ section, paragraph 2 |
| Synthesis methods | 12a | Describe the methods used to summarize or synthesize results and provide a rationale for the choice(s). | ‘Data synthesis and analysis’ section, paragraph 1 |
|  | 12b | Describe any methods used to explore possible causes of heterogeneity among results. | ‘Data synthesis and analysis’ section, paragraph 1 |
|  | 12c | Describe any sensitivity analyses conducted to assess the robustness of the synthesized results. | Not applicable- quantitative syntheses were not performed |
| Reporting bias assessment | 13 | Describe the methods used to collect data on (from the systematic reviews) and/or assess the risk of bias due to missing results in a summary or synthesis (arising from reporting biases at the levels of the systematic reviews, primary studies, and supplemental primary studies, if included). | ‘Risk of bias assessment’ section, paragraph 2; ‘Certainty of evidence assessment’ section, paragraph 1 |
| Certainty assessment | 14 | Describe the methods used to collect data on (from the systematic reviews) and/or assess certainty (or confidence) in the body of evidence for an outcome. | ‘Certainty of evidence assessment’ section, paragraph 1 |
| **RESULTS** | | | |
| Systematic review and supplemental primary study selection | 15a | Describe the results of the search and selection process, including the number of records screened, assessed for eligibility, and included in the overview of reviews, ideally with a flow diagram. | ‘Study selection and characteristics’ section, paragraph 1; Figure 1. |
|  | 15b | Provide a list of studies that might appear to meet the inclusion criteria, but were excluded, with the main reason for exclusion. | ‘Study selection and characteristics’ section, paragraph 1 |
| Characteristics of systematic reviews and supplemental primary studies | 16 | Cite each included systematic review and supplemental primary study (if included) and present its characteristics. | ‘Study selection and characteristics’ section, paragraphs 1-5; Appendix 4 |
| Primary study overlap | 17 | Describe the extent of primary study overlap across the included systematic reviews. | ‘Study selection and characteristics’ section, paragraph 1 |
| Risk of bias in systematic reviews, primary studies, and supplemental primary studies | 18a | Present assessments of risk of bias or methodological quality for each included systematic review. | ‘Risk of bias’ section, paragraph 1; Table 3 |
|  | 18b | Present assessments (collected from systematic reviews or assessed anew) of the risk of bias of the primary studies included in the systematic reviews. | ‘Risk of bias’ section, paragraph 1; Appendix 4 |
|  | 18c | Present assessments of the risk of bias of supplemental primary studies (if included). | ‘Risk of bias’ section, paragraph 2; Table 4 |
| Summary or synthesis of results | 19a | For all outcomes, summarize the evidence from the systematic reviews and supplemental primary studies (if included). If meta-analyses were done, present for each the summary estimate and its precision and measures of statistical heterogeneity. If comparing groups, describe the direction of the effect. | ‘Review findings’ section (all subsections); Table 5 |
|  | 19b | If meta-analyses were done, present results of all investigations of possible causes of  heterogeneity. | Not applicable |
|  | 19c | If meta-analyses were done, present results of all sensitivity analyses conducted to assess the robustness of synthesized results. | Not applicable |
| Reporting biases | 20 | Present assessments (collected from systematic reviews and/or assessed anew) of the risk of bias due to missing primary studies, analyses, or results in a summary or synthesis (arising from reporting biases at the levels of the systematic reviews, primary studies, and supplemental primary studies, if included) for each summary or synthesis assessed. | ‘Risk of bias’ section, paragraph 2; Table 4; Appendix 5. |
| Certainty of evidence | 21 | Present assessments (collected or assessed anew) of certainty (or confidence) in the body of evidence for each outcome. | ‘Review findings’ section (all subsections); Appendix 5 |
| **DISCUSSION** | | | |
| Discussion | 22a | Summarize the main findings, including any discrepancies in findings across the included  systematic reviews and supplemental primary studies (if included). | ‘Discussion’ section, paragraphs 2, 3. |
|  | 22b | Provide a general interpretation of the results in the context of other evidence. | ‘Discussion’ section, paragraphs 2-4, ‘What this review does not answer’ subsection. |
|  | 22c | Discuss any limitations of the evidence from systematic reviews, their primary studies, and supplemental primary studies (if included) included in the overview of reviews. Discuss any limitations of the overview of reviews methods used. | ‘Limitations’ section, paragraph 1. |
|  | 22d | Discuss implications for practice, policy, and future research (both systematic reviews and  primary research). Consider the relevance of the findings to the end users of the overview of reviews, e.g., healthcare providers, policymakers, patients, among others. | ‘Results’ section, ‘Recommendations for clinical practice’ subsection; ‘Discussion’ section, paragraph 2; Figure 2; ‘Future research’ section, paragraph 1. |
| **OTHER INFORMATION** | | | |
| Registration and protocol | 23a | Provide registration information for the overview of reviews, including register name and  registration number, or state that the overview of reviews was not registered. | ‘Methods’ section, paragraph 2. |
|  | 23b | Indicate where the overview of reviews protocol can be accessed, or state that a protocol was not prepared. | ‘Methods’ section, paragraph 2. |
|  | 23c | Describe and explain any amendments to information provided at registration or in the protocol. Indicate the stage of the overview of reviews at which amendments were made. | Not applicable |
| Support | 24 | Describe sources of financial or non-financial support for the overview of reviews, and the role of the funders or sponsors in the overview of reviews. | ‘Financial support’ section |
| Competing interests | 25 | Declare any competing interests of the overview of reviews' authors. | ‘Declaration of competing interests’ form |
| Author information | 26a | Provide contact information for the corresponding author. | Title page |
|  | 26b | Describe the contributions of individual authors and identify the guarantor of the overview of reviews. | ‘Author contributions’ section |
| Availability of data and other materials | 27 | Report which of the following are available, where they can be found, and under which conditions they may be accessed: template data collection forms; data collected from included systematic reviews and supplemental primary studies; analytic code; any other materials used in the overview of reviews. | ‘Data availability’ statement |

**PRISMA 2020 Checklist (2) for systematic reviews**

| **Section and Topic** | **Item #** | **Checklist item** | **Location where item is reported** |
| --- | --- | --- | --- |
| **TITLE** | | |  |
| Title | 1 | Identify the report as a systematic review. | Title |
| **ABSTRACT** | | |  |
| Abstract | 2 | See the PRISMA 2020 for Abstracts checklist. | See below extension checklist |
| **INTRODUCTION** | | |  |
| Rationale | 3 | Describe the rationale for the review in the context of existing knowledge. | ‘Introduction’ section, paragraphs 2-4 |
| Objectives | 4 | Provide an explicit statement of the objective(s) or question(s) the review addresses. | ‘Introduction’ section, paragraph 5 |
| **METHODS** | | |  |
| Eligibility criteria | 5 | Specify the inclusion and exclusion criteria for the review and how studies were grouped for the syntheses. | ‘Search strategy and study selection’ section, paragraph 2, table 1; ‘Methods’ section paragraph 1 |
| Information sources | 6 | Specify all databases, registers, websites, organisations, reference lists and other sources searched or consulted to identify studies. Specify the date when each source was last searched or consulted. | ‘Search strategy and study selection’ section, paragraph 1 |
| Search strategy | 7 | Present the full search strategies for all databases, registers and websites, including any filters and limits used. | ‘Appendix 3. Systematic search strategies’ |
| Selection process | 8 | Specify the methods used to decide whether a study met the inclusion criteria of the review, including how many reviewers screened each record and each report retrieved, whether they worked independently, and if applicable, details of automation tools used in the process. | ‘Search strategy and study selection’ section, paragraph 1 |
| Data collection process | 9 | Specify the methods used to collect data from reports, including how many reviewers collected data from each report, whether they worked independently, any processes for obtaining or confirming data from study investigators, and if applicable, details of automation tools used in the process. | ‘Data extraction’ section, paragraph 1 |
| Data items | 10a | List and define all outcomes for which data were sought. Specify whether all results that were compatible with each outcome domain in each study were sought (e.g. for all measures, time points, analyses), and if not, the methods used to decide which results to collect. | ‘Data extraction’ section, paragraph 1; Tables 1 and 2 |
|  | 10b | List and define all other variables for which data were sought (e.g. participant and intervention characteristics, funding sources). Describe any assumptions made about any missing or unclear information. | ‘Data extraction’ section, paragraph 1 |
| Study risk of bias assessment | 11 | Specify the methods used to assess risk of bias in the included studies, including details of the tool(s) used, how many reviewers assessed each study and whether they worked independently, and if applicable, details of automation tools used in the process. | ‘Risk of bias assessment’ section, paragraph 2 |
| Effect measures | 12 | Specify for each outcome the effect measure(s) (e.g. risk ratio, mean difference) used in the synthesis or presentation of results. | ‘Data extraction’ section, paragraph 1 |
| Synthesis methods | 13a | Describe the processes used to decide which studies were eligible for each synthesis (e.g. tabulating the study intervention characteristics and comparing against the planned groups for each synthesis (item #5)). | Not applicable |
|  | 13b | Describe any methods required to prepare the data for presentation or synthesis, such as handling of missing summary statistics, or data conversions. | ‘Data extraction’ section, paragraph 1 |
|  | 13c | Describe any methods used to tabulate or visually display results of individual studies and syntheses. | ‘Data synthesis and analysis’ section, paragraph 1 |
|  | 13d | Describe any methods used to synthesize results and provide a rationale for the choice(s). If meta-analysis was performed, describe the model(s), method(s) to identify the presence and extent of statistical heterogeneity, and software package(s) used. | ‘Data synthesis and analysis’ section, paragraph 1 |
|  | 13e | Describe any methods used to explore possible causes of heterogeneity among study results (e.g. subgroup analysis, meta-regression). | Not applicable |
|  | 13f | Describe any sensitivity analyses conducted to assess robustness of the synthesized results. | Not applicable |
| Reporting bias assessment | 14 | Describe any methods used to assess risk of bias due to missing results in a synthesis (arising from reporting biases). | ‘Risk of bias assessment’ section, paragraph 2; ‘Certainty of evidence assessment’ section, paragraph 1 |
| Certainty assessment | 15 | Describe any methods used to assess certainty (or confidence) in the body of evidence for an outcome. | ‘Certainty of evidence assessment’ section, paragraph 1 |
| **RESULTS** | | |  |
| Study selection | 16a | Describe the results of the search and selection process, from the number of records identified in the search to the number of studies included in the review, ideally using a flow diagram. | ‘Study selection and characteristics’ section, paragraph 1; Figure 1. |
|  | 16b | Cite studies that might appear to meet the inclusion criteria, but which were excluded, and explain why they were excluded. | ‘Study selection and characteristics’ section, paragraph 1; Figure 1. |
| Study characteristics | 17 | Cite each included study and present its characteristics. | ‘Study selection and characteristics’ section, paragraph 1; Appendix 4 |
| Risk of bias in studies | 18 | Present assessments of risk of bias for each included study. | ‘Risk of bias’ section, paragraph 2; Table 4 |
| Results of individual studies | 19 | For all outcomes, present, for each study: (a) summary statistics for each group (where appropriate) and (b) an effect estimate and its precision (e.g. confidence/credible interval), ideally using structured tables or plots. | Appendix 4 |
| Results of syntheses | 20a | For each synthesis, briefly summarise the characteristics and risk of bias among contributing studies. | ‘Review findings’ section (all subsections); Table 5 |
|  | 20b | Present results of all statistical syntheses conducted. If meta-analysis was done, present for each the summary estimate and its precision (e.g. confidence/credible interval) and measures of statistical heterogeneity. If comparing groups, describe the direction of the effect. | Not applicable |
|  | 20c | Present results of all investigations of possible causes of heterogeneity among study results. | Not applicable |
|  | 20d | Present results of all sensitivity analyses conducted to assess the robustness of the synthesized results. | Not applicable |
| Reporting biases | 21 | Present assessments of risk of bias due to missing results (arising from reporting biases) for each synthesis assessed. | ‘Risk of bias’ section, paragraph 2; Table 4; Appendix 5. |
| Certainty of evidence | 22 | Present assessments of certainty (or confidence) in the body of evidence for each outcome assessed. | ‘Review findings’ section (all subsections); Appendix 5 |
| **DISCUSSION** | | |  |
| Discussion | 23a | Provide a general interpretation of the results in the context of other evidence. | ‘Discussion’ section, paragraphs 2-4; ‘What this review does not answer’ subsection. |
|  | 23b | Discuss any limitations of the evidence included in the review. | ‘Limitations’ section, paragraph 1. |
|  | 23c | Discuss any limitations of the review processes used. | ‘Limitations’ section, paragraph 1. |
|  | 23d | Discuss implications of the results for practice, policy, and future research. | ‘Discussion’ section, paragraph 2; Figure 2; ‘Future research’ section, paragraph 1. |
| **OTHER INFORMATION** | | |  |
| Registration and protocol | 24a | Provide registration information for the review, including register name and registration number, or state that the review was not registered. | ‘Methods’ section, paragraph 2. |
|  | 24b | Indicate where the review protocol can be accessed, or state that a protocol was not prepared. | ‘Methods’ section, paragraph 2. |
|  | 24c | Describe and explain any amendments to information provided at registration or in the protocol. | Not applicable |
| Support | 25 | Describe sources of financial or non-financial support for the review, and the role of the funders or sponsors in the review. | ‘Financial support’ section |
| Competing interests | 26 | Declare any competing interests of review authors. | ‘Declaration of competing interests’ form |
| Availability of data, code and other materials | 27 | Report which of the following are publicly available and where they can be found: template data collection forms; data extracted from included studies; data used for all analyses; analytic code; any other materials used in the review. | ‘Data availability’ statement |

**PRISMA 2020 extension for abstracts (2)**

| **Section and Topic** | **Item #** | **Checklist item** | **Reported (Yes/No)** |
| --- | --- | --- | --- |
| **TITLE** | | |  |
| Title | 1 | Identify the report as a systematic review. | Yes |
| **BACKGROUND** | | |  |
| Objectives | 2 | Provide an explicit statement of the main objective(s) or question(s) the review addresses. | Yes |
| **METHODS** | | |  |
| Eligibility criteria | 3 | Specify the inclusion and exclusion criteria for the review. | Yes |
| Information sources | 4 | Specify the information sources (e.g. databases, registers) used to identify studies and the date when each was last searched. | Yes |
| Risk of bias | 5 | Specify the methods used to assess risk of bias in the included studies. | Yes |
| Synthesis of results | 6 | Specify the methods used to present and synthesise results. | Yes |
| **RESULTS** | | |  |
| Included studies | 7 | Give the total number of included studies and participants and summarise relevant characteristics of studies. | Yes |
| Synthesis of results | 8 | Present results for main outcomes, preferably indicating the number of included studies and participants for each. If meta-analysis was done, report the summary estimate and confidence/credible interval. If comparing groups, indicate the direction of the effect (i.e. which group is favoured). | Yes |
| **DISCUSSION** | | |  |
| Limitations of evidence | 9 | Provide a brief summary of the limitations of the evidence included in the review (e.g. study risk of bias, inconsistency and imprecision). | Yes |
| Interpretation | 10 | Provide a general interpretation of the results and important implications. | Yes |
| **OTHER** | | |  |
| Funding | 11 | Specify the primary source of funding for the review. | Yes |
| Registration | 12 | Provide the register name and registration number. | Yes |

**References**

1. Gates M, Gates A, Pieper D, Fernandes RM, Tricco AC, Moher D, et al. Reporting guideline for overviews of reviews of healthcare interventions: development of the PRIOR statement. BMJ. 2022;378:e070849.

2. Page MJ, McKenzie JE, Bossuyt PM, Boutron I, Hoffmann TC, Mulrow CD, et al. The PRISMA 2020 statement: an updated guideline for reporting systematic reviews. BMJ. 2021;372:n71.

3. Johnston A, Smith C, Zheng C, Aaron SD, Kelly SE, Skidmore B, et al. Influence of prolonged treatment with omalizumab on the development of solid epithelial cancer in patients with atopic asthma and chronic idiopathic urticaria: A systematic review and meta-analysis. Clin Exp Allergy. 2019;49(10):1291-305.

## Appendix 2. Supplementary methods

**Protocol amendment**

The eligibility criteria for setting relative to study design was amended to include observational studies located outside of an ICU setting. This enabled the inclusion of the full body of evidence pertaining to CXR, laboratory, or viral testing across patient subgroups.

**Study selection**

**Table A3.A** **Judgments for inclusion and exclusion of overlapping systematic reviews**

| **Topic** | **Study ID** | **Citation** | **Judgment** | **Final rating** |
| --- | --- | --- | --- | --- |
| Chest Xray | Bordley 2004 | Bordley WC, Viswanathan M, King VJ, Sutton SF, Jackman AM, Sterling L, Lohr KN. Diagnosis and testing in bronchiolitis: a systematic review. Archives of pediatrics & adolescent medicine. 2004 Feb 1;158(2):119-26. | Some overlapping evidence with Williams 2012. More dated and less comprehensive evidence overall. | Exclude |
|  | Williams 2012 | Williams C, Bartram T. Towards evidence based emergency medicine: best BETs from the Manchester Royal Infirmary. BET 4: Chest x-rays in bronchiolitis. Emergency medicine journal: EMJ. 2012 Jun;29(6):514-5. | Includes more recent evidence and as a result is more comprehensive than Bordley 2004. | Include |
| Laboratory testing | Bordley 2004 | Bordley WC, Viswanathan M, King VJ, Sutton SF, Jackman AM, Sterling L, Lohr KN. Diagnosis and testing in bronchiolitis: a systematic review. Archives of pediatrics & adolescent medicine. 2004 Feb 1;158(2):119-26. | No results reported for the review outcomes. | Exclude |
|  | Ralston 2011 | Ralston S, Hill V, Waters A. Occult serious bacterial infection in infants younger than 60 to 90 days with bronchiolitis: a systematic review. Archives of pediatrics & adolescent medicine. 2011 Oct 3;165(10):951-6. | Reports overlapping evidence to McDaniel 2019. More dated and less comprehensive evidence overall. | Exclude |
|  | McDaniel 2019 | McDaniel CE, Ralston S, Lucas B, Schroeder AR. Association of diagnostic criteria with urinary tract infection prevalence in bronchiolitis: a systematic review and meta-analysis. JAMA pediatrics. 2019 Mar 1;173(3):269-77. | Presents the most recent and comprehensive evidence on UTI in bronchiolitis, and includes meta-analysis. | Include |
| Viral testing | Bordley 2004 | Bordley WC, Viswanathan M, King VJ, Sutton SF, Jackman AM, Sterling L, Lohr KN. Diagnosis and testing in bronchiolitis: a systematic review. Archives of pediatrics & adolescent medicine. 2004 Feb 1;158(2):119-26. | Reports no trials were identified that investigated whether knowing RSV is the causative agent affects clinical outcomes in bronchiolitis. Does not report any results for our review outcomes. | Exclude |
|  | Ambrozej 2024 | Ambrożej D, Orzołek I, Makrinioti H, Castro-Rodriguez JA, Camargo Jr CA, Hasegawa K, Papadopoulos NG, Gern JE, Nino G, da Silva LV, Takeyama A. Association of respiratory virus types with clinical features in bronchiolitis: Implications for virus testing strategies. A systematic review and meta-analysis. Paediatric respiratory reviews. 2024 Mar 1;49:34-42 | Presents a systematic review with meta-analysis on the association of respiratory virus types with clinical outcomes in bronchiolitis. | Include |

**Risk of bias assessment**

The following thresholds were used to convert Newcastle Ottawa Scale (NOS) scores to the Agency for Healthcare Research and Quality (NHRQ) study quality standards (good, fair poor), based on prior research by the NOS developers (3).

| **AHRQ Study Quality Standard** | **NOS criteria** |
| --- | --- |
| Good quality | - Selection domain: 3 or 4 stars; AND - Comparability domain: 1 or 2 stars; AND - Outcome/ exposure domain: 2 or 3 stars |
| Fair quality | - Selection domain: 2 stars; AND - Comparability domain: 1 or 2 stars; - Outcome/ exposure domain: 2 or 3 stars |
| Poor quality | - Selection domain: 0 or 1 star; OR - Comparability domain: 0 stars; OR - Outcome/ exposure domain: 0 or 1 stars |

## Appendix 3. Systematic search strategies

**Ovid MEDLINE(R) ALL <1946 to June 19, 2023> (search updated February 19, 2025)**

Search date: 21/06/23

1 bronchiolitis/ or bronchiolitis, viral/ or respiratory syncytial viruses/ or respiratory syncytial virus, human/ or Respiratory Syncytial Virus Infections/ or (bronchiolit* or wheez* or (Respiratory adj1 Syncytial adj1 Virus*)).af. or rsv.tw. 48765

2 limit 1 to (case reports or comment or editorial or letter) 6402

3 limit 1 to (clinical trial, all or clinical trial, phase i or clinical trial, phase ii or clinical trial, phase iii or clinical trial, phase iv or clinical trial or controlled clinical trial or guideline or meta analysis or practice guideline or randomized controlled trial or "review" or systematic reviews) 8638

4 1 and exp Evidence-Based Medicine/ 135

5 (1 not 2) or 3 or 4 42783

6 *bronchiolitis, viral/bl, ci, cl, co, dg, di, dt, ep, et, ge, hi, im, mi, mo, pa, pp, pc, th, ur, vi 1012

7 *bronchiolitis/bl, ci, cl, co, dg, di, dt, ep, et, ge, hi, im, mi, mo, pa, pp, pc, th, ur, vi 2446

8 6 or 7 3450

9 limit 8 to (case reports or comment or editorial or letter) 805

10 limit 8 to (clinical trial, all or clinical trial, phase i or clinical trial, phase ii or clinical trial, phase iii or clinical trial, phase iv or clinical trial or controlled clinical trial or guideline or meta analysis or practice guideline or randomized controlled trial or "review" or systematic reviews) 877

11 8 and exp Evidence-Based Medicine/ 64

12 (8 not 9) or 10 or 11 2702

13 Natural History/ or exp Epidemiology/ 29277

14 exp "reproducibility of results"/ or (scoring adj1 system*).tw. 503239

15 "severity of illness index"/ or (disease adj1 severity).tw. 311661

16 diagnosis, differential/ 467890

17 physical examination/ or exp auscultation/ or blood pressure determination/ or exp palpation/ or percussion/ or pulse/ or exp vital signs/ 517012

18 exp *Respiratory Tract Infections/ 529915

19 risk factors/ 955289

20 "length of stay"/ or patient admission/ or patient discharge/ or (criteria adj4 discharge).tw. 156283

21 exp intensive care units, pediatric/ or respiratory care units/ or (nicu or icu or picu or intensive-care).tw. 231239

22 morbidity/ or prevalence/ or exp mortality/ 781380

23 exp Diagnostic Imaging/ or radiography, thoracic/ or ((chest adj1 x-ray*) or (chest adj1 xray*)).tw. 2929342

24 exp Hematologic Tests/ or ((blood adj1 test*) or (blood adj1 exam*) or (virologic* adj1 investigation*)).tw. 297077

25 Urinalysis/ 9268

26 Nasal Lavage Fluid/ or ((nose or nasal) adj1 (mucus or mucosa)).tw. 10677

27 Emergency Service, Hospital/ or (emergency adj1 department*).tw. 157373

28 asthma/ or hypersensitivity, immediate/ or (atopy or atopic).tw. 186484

29 (salbutamol or ventolin or levalbuterol or adrenalin* or epinephrin* or (beta* adj2 adrenergic*) or (beta* adj2 agonist*) or ics or (inhaled adj1 corticosteroid*) or montelukast).tw. 133389

30 exp Albuterol/ad, tu 7239

31 exp Epinephrine/ad, tu 10031

32 exp Bronchodilator Agents/ad, tu or bronchodilat*.tw. 63186

33 exp steroids/ad, tu 245236

34 exp Cholinergic Antagonists/ or receptors, adrenergic, beta-2/ or (cholinergic adj1 receptor adj1 block* adj1 agent*).tw. 91309

35 exp Anti-Inflammatory Agents/ 568228

36 exp Adrenal Cortex Hormones/ or (corticosteroid* or (cortico adj1 steroid*) or glucocorticoid* or gluco corticoid*).tw. 513603

37 Leukotriene Antagonists/ or (Leukotriene adj1 receptor adj1 block* adj1 agent*).tw. 3242

38 Saline Solution, Hypertonic/ad, tu or (hypertonic and (saline adj1 solution)).tw. 3206

39 exp Aerosols/ and exp Sodium Chloride/ 410

40 exp "nebulizers and vaporizers"/ and exp sodium chloride/ 116

41 ((aerosoli#ed adj1 saline) or (nebuli#ed adj1 saline)).tw. 203

42 exp Oxygen Inhalation Therapy/ or *Oxygen/ad, st [Administration & Dosage, Standards] 30943

43 (exp Oximetry/ or oximet*.tw.) and (exp "reproducibility of results"/ or (reliability or function or (technical adj1 specification*) or (percutaneous adj1 measurement*)).tw. or exp blood gas analysis/ or ((pulse adj1 oximet*) or (supplementa* adj1 oxygen) or (oxygen adj1 saturation) or (oxygen adj1 therap*) or (oxygen adj1 treatment*)).tw.) 22242

44 continuous positive airway pressure/ or positive pressure respiration/ or (bubble adj1 CPAP).tw. 27198

45 exp Physical Therapy Modalities/ 178123

46 Physical Therapy Specialty/ or physical therapists/ 5873

47 (physiotherap* or (physical adj therap*)).tw. 59298

48 ((nasal* or nose or naso) adj3 suction*).tw. 122

49 suction/ or (deep adj1 suction*).tw. 13138

50 saline.tw. and Administration, Intranasal/ 756

51 ((saline adj1 drop*) or (nasal adj1 saline)).tw. 483

52 exp Fluid Therapy/ or Rehydration Solutions/ 22592

53 enteral nutrition/ or exp parenteral nutrition/ or intubation, gastrointestinal/ 50460

54 (((non adj1 oral) or oral) and (feed* or hydration or fluid* or solution* or therap*)).tw. 211378

55 exp bacterial infections/ 961373

56 exp otitis media/ 25757

57 exp Meningitis/ 58718

58 exp *anti-bacterial agents/tu or (antibiotic* or 3z or aruzilina or atizor or azadose or azasite or azatril or azenil or azibiot or azibiot-neo or azimin or azithral or azithromycin or azitrocin or azitromax or azitromicin or azitromicina or aziwok or azomyne or aztrin or azydrop or azyter or azythromycin or bazyt or cp-62933 or cp-62993 or cp62933 or cp62993 or forcin or inedol or infectoazit or isv-401 or isv401 or kromicin or macrozit or mezatrin or octavax or ordipha or ribotrex or sumamed or sunamed or tobyl or tromix or trozocina or ultreon or vinzam or xithrone or xz-450 or xz450 or zaret or zarom or zetamax or zeto or zibramax or zifin or zimericina or zistic or zithromax or zithrox or zitinn or zitrim or zitrobifan or zitrocin or zitromax or zmax).tw. 490886

59 exp Sepsis/ 141157

60 exp Urinary Tract Infections/ 50669

61 Tracheitis/ 1560

62 (serious adj1 bacterial adj1 infection*).tw. 1294

63 infection control/ or exp primary prevention/ or patient isolation/ 213304

64 COVID-19/ or SARS-CoV-2/ 234537

65 (2019-novel or 2019nCoV or 2019-nCoV or COVID-19 or COVID19 or COVID-2019 or COVID2019 or CONVID-19 or CONVID19 or CORVID-19 or CORVID19 or CoV2 or CoV-2 or HCoV* or Ncov* or Ncorona* or Ncorono* or NcovChina* or NcovChinese* or NcovHubei* or NcovWuhan* or SARS2 or SARS-2 or SARScoronavirus2 or SARScoronavirus-2 or SARScoronovirus2 or SARScoronovirus-2 or SARSCov19 or SARSCov-19 or SARS-CoV-2 or SARSCoV-2 or SARSCoV2 or WN-CoV or WNCoV or wuhan-virus).tw. 341860

66 ((pneumonia* or outbreak* or respiratory-illness* or respiratory-disease* or respiratory-symptom* or seafood-market* or food-market* or wildlife) and (Wuhan or China or Chinese or Hubei or Huanan)).tw. 21170

67 ((new or novel or nouveau or risk factors or "2019" or Wuhan or Hubei or Huanan or China or Chinese) adj3 (coronavirus* or corona virus* or betacoronavirus* or CoV or HCoV)).tw. 75998

68 POST-ACUTE COVID-19 SYNDROME/ or (longCOVID* or postCOVID* or postcoronavirus* or postSARS*).tw. 2221

69 (coronavirus/ or betacoronavirus/ or coronavirus infections/) and (disease outbreaks/ or epidemics/ or pandemics/) 40238

70 ((coronavirus* or corona-virus* or betacoronavirus*) adj3 (pandemic* or epidemic* or outbreak* or crisis)).tw. 15507

71 exp Antibodies, Monoclonal/ or (monoclonal-antibod* or clonal-antibod* or hybridoma-antibod* or nirsevimab or medi-8897 or medi8897 or sp-0232 or sp-232 or sp0232 or sp232 or Motavizumab or medi-524 or medi524 or numax or Palivizumab or abbosynagis or medi-493 or medi493 or synagis or synagys).tw. 362383

72 or/13-71 9536442

73 5 and 72 28221

74 exp pregnant women/ or exp pregnancy/ or prenatal care/ or (parturition or ante-natal or antenatal* or pre-natal* or prenatal* or pregnan*).tw. 1176170

75 exp immunization/ or (immunis* or immuniz* or vaccin*).tw. 554939

76 (Respiratory-syncytial-virus-vaccine* or RSV-vaccine* or Arexvy).tw. 910

77 (74 and 75 and 5) or (74 and 76) 271

78 (75 and 5) or 76 4709

79 12 or 73 or 78 29996

80 (newborn* or new-born* or baby or babies or neonat* or neo-nat* or infan* or toddler* or aged-1 or aged-one or one-year-old or 1-year-old or under-two or under-2 or younger-than-two or younger-than-2 or below-two or below-2 or under-24-months or younger-than-24-months or below-24-months or aged-1-to-23-months or aged-one-to-twenty-three-months).tw,kf,hw. 1692458

81 exp Bronchiolitis Obliterans/ or (bronchiolitis adj1 obliterans).af. 5735

82 (79 and 80) not 81 13211

83 77 not 81 271

84 82 or 83 13251

85 limit 84 to (english language and yr="2000 -Current") 9850

**Embase <1974 to 2023 June 19> (search updated February 19, 2025)**

Search date: 21/06/23

1 bronchiolitis/ or viral bronchiolitis/ or exp human respiratory syncytial virus/ or respiratory syncytial virus infection/ or (bronchiolit* or wheez* or (Respiratory adj1 Syncytial adj1 Virus*)).af. or rsv.tw. 93187

2 limit 1 to (editorial or letter or note) 7059

3 1 and (exp controlled clinical trial/ or exp practice guideline/ or meta analysis/ or "review"/ or "systematic review"/) 16414

4 1 and exp evidence based medicine/ 6467

5 (1 not 2) or 3 or 4 86742

6 *bronchiolitis/co, di, dm, dr, dt, ep, et, pc, rt, th 3796

7 *viral bronchiolitis/co, di, dm, dr, dt, ep, et, pc, th 548

8 6 or 7 4336

9 limit 8 to (editorial or letter or note) 571

10 8 and (exp controlled clinical trial/ or exp practice guideline/ or meta analysis/ or "review"/ or "systematic review"/) 1137

11 8 and exp evidence based medicine/ 555

12 (8 not 9) or 10 or 11 3866

13 History/ or exp Epidemiology/ 4735239

14 reproducibility/ or exp validity/ or scoring system/ or exp reliability/ 847991

15 exp disease severity/ or (severity adj2 illness).tw. 2249656

16 differential diagnosis/ or physical examination/ or palpation/ or percussion/ or blood pressure measurement/ or blood pressure monitoring/ or pulse rate/ or vital sign/ or ascultation.tw. 813575

17 exp *respiratory tract infection/ 235051

18 risk factor/ 1336657

19 "length of stay"/ or hospital admission/ or hospital discharge/ or (criteria adj4 discharge).tw. 638513

20 intensive care unit/ or medical intensive care unit/ or neonatal intensive care unit/ or pediatric intensive care unit/ or (nicu or icu or picu or intensive-care).tw. 452434

21 morbidity/ or prevalence/ or mortality/ or childhood mortality/ or infant mortality/ 2024864

22 exp diagnostic imaging/ or exp thorax radiography/ or ((chest adj1 x-ray*) or (chest adj1 xray*)).tw. 485079

23 exp blood examination/ or ((blood adj1 test*) or (haem* adj1 exam*) or (haem* adj1 test*) or (virologic* adj1 investigation*)).tw. 370894

24 exp urinalysis/ 127990

25 nose mucus/ or nose mucosa/ or (nasal adj1 lavage adj1 fluid*).tw. 18815

26 (emergency adj1 department*).tw. 193543

27 exp asthma/ or atopy/ 315735

28 (salbutamol or ventolin or levalbuterol or adrenalin* or epinephrin* or (beta* adj2 adrenergic*) or (beta* adj2 agonist*) or ics or (inhaled adj1 corticosteroid*) or montelukast).tw. 178202

29 salbutamol/ad, do, dt 18740

30 epinephrine/ad, do, dt 5593

31 exp bronchodilating agent/ad, do, dt or bronchodilat*.tw. 99057

32 exp steroid/ad, do, dt 649505

33 exp cholinergic receptor blocking agent/ or (cholinergic adj1 antagonist*).tw. 318374

34 exp antiinflammatory agent/ 2480733

35 exp corticosteroid/ or (corticosteroid* or (cortico adj1 steroid*) or glucocorticoid* or gluco corticoid*).tw. 1152309

36 exp leukotriene receptor blocking agent/ or (leukotriene adj1 antagonist*).tw. 23668

37 (sodium chloride/ad, do, dt and (aerosol/ or hypertonic solution/ or exp nebulizer/ or vaporizer/)) or (hypertonic and (saline adj1 solution)).tw. 1308

38 ((aerosoli#ed adj1 saline) or (nebuli#ed adj1 saline)).tw. 284

39 exp oximetry/ or exp oxygen therapy/ or oxygen/ad, do 124833

40 (exp oximetry/ or oximet*.tw.) and (reproducibility/ or (reliability or function or (technical adj1 specification*) or (percutaneous adj1 measurement*)).tw. or exp blood gas analysis/ or ((pulse adj1 oximet*) or (supplementa* adj1 oxygen) or (oxygen adj1 saturation) or (oxygen adj1 therap*) or (oxygen adj1 treatment*)).tw.) 24303

41 positive end expiratory pressure ventilation/ or ((continuous adj1 positive adj1 airway adj1 pressure) or (positive adj1 pressure adj1 respiration) or (bubble adj1 CPAP)).tw. 22005

42 exp physiotherapy/ 107415

43 (physiotherap* or physical therap*).tw. 96671

44 ((nasal* or nose or naso) adj3 suction*).tw. 183

45 suction/ or (deep adj1 suction*).tw. 12931

46 sodium chloride/na [Intranasal Drug Administration] 177

47 ((saline adj1 drop*) or (nasal adj1 saline)).tw. 667

48 enteric feeding/ or exp parenteral nutrition/ or exp digestive tract intubation/ 92632

49 (((non adj1 oral) or oral) and (feed* or hydration or fluid* or solution* or therap*)).tw. 337435

50 exp fluid therapy/ or oral rehydration solution/ 112397

51 exp bacterial infection/ 955606

52 exp otitis media/ 38079

53 exp meningitis/ 114158

54 exp *antiinfective agent/dt or (antibiotic* or 3z or aruzilina or atizor or azadose or azasite or azatril or azenil or azibiot or azibiot-neo or azimin or azithral or azithromycin or azitrocin or azitromax or azitromicin or azitromicina or aziwok or azomyne or aztrin or azydrop or azyter or azythromycin or bazyt or cp-62933 or cp-62993 or cp62933 or cp62993 or forcin or inedol or infectoazit or isv-401 or isv401 or kromicin or macrozit or mezatrin or octavax or ordipha or ribotrex or sumamed or sunamed or tobyl or tromix or trozocina or ultreon or vinzam or xithrone or xz-450 or xz450 or zaret or zarom or zetamax or zeto or zibramax or zifin or zimericina or zistic or zithromax or zithrox or zitinn or zitrim or zitrobifan or zitrocin or zitromax or zmax).tw. 977691

55 exp sepsis/ 334492

56 exp urinary tract infection/ 139560

57 exp tracheitis/ 4182

58 (serious adj1 bacterial adj1 infection*).tw. 1891

59 infection control/ or patient care/ or isolation.tw. 787000

60 coronavirus disease 2019/ or experimental coronavirus disease 2019/ 363085

61 (2019-novel or 2019nCoV or 2019-nCoV or COVID-19 or COVID19 or COVID-2019 or COVID2019 or CONVID-19 or CONVID19 or CORVID-19 or CORVID19 or CoV2 or CoV-2 or HCoV* or Ncov* or Ncorona* or Ncorono* or NcovChina* or NcovChinese* or NcovHubei* or NcovWuhan* or SARS2 or SARS-2 or SARScoronavirus2 or SARScoronavirus-2 or SARScoronovirus2 or SARScoronovirus-2 or SARSCov19 or SARSCov-19 or SARS-CoV-2 or SARSCoV-2 or SARSCoV2 or WN-CoV or WNCoV or wuhan-virus).tw. 418425

62 ((pneumonia* or outbreak* or respiratory-illness* or respiratory-disease* or respiratory-symptom* or seafood-market* or food-market* or wildlife) and (Wuhan or China or Chinese or Hubei or Huanan)).tw. 23979

63 ((new or novel or nouveau or ("length of stay" or hospital admission or hospital discharge or (criteria adj4 discharge)) or "2019" or Wuhan or Hubei or Huanan or China or Chinese) adj3 (coronavirus* or corona virus* or betacoronavirus* or CoV or HCoV)).tw. 85970

64 long COVID/ or (longCOVID* or postCOVID* or postcoronavirus* or postSARS*).tw. 4909

65 (coronavirinae/ or betacoronavirus/ or coronavirus infection/) and (epidemic/ or pandemic/) 9531

66 ((coronavirus* or corona-virus* or betacoronavirus*) adj3 (pandemic* or epidemic* or outbreak* or crisis)).tw. 17154

67 severe-acute-respiratory-syndrome-coronavirus-2.hw. 97668

68 coronavirus-disease-2019.hw. 363661

69 exp Monoclonal antibody/ or (monoclonal-antibod* or clonal-antibod* or hybridoma-antibod* or nirsevimab or medi-8897 or medi8897 or sp-0232 or sp-232 or sp0232 or sp232 or Motavizumab or medi-524 or medi524 or numax or Palivizumab or abbosynagis or medi-493 or medi493 or synagis or synagys).tw. 833604

70 or/13-69 14210720

71 5 and 70 69784

72 pregnant woman/ or exp pregnancy/ or prenatal care/ or (parturition or ante-natal or antenatal* or pre-natal* or prenatal* or pregnan*).tw. 1159109

73 exp immunization/ or (immunis* or immuniz* or vaccin*).tw. 656913

74 (Respiratory-syncytial-virus-vaccine* or RSV-vaccine* or Arexvy).tw. 1061

75 (72 and 73 and 5) or (72 and 74) 512

76 (73 and 5) or 74 8119

77 12 or 71 or 76 71881

78 (newborn* or new-born* or baby or babies or neonat* or neo-nat* or infan* or toddler* or aged-1 or aged-one or one-year-old or 1-year-old or under-two or under-2 or younger-than-two or younger-than-2 or below-two or below-2 or under-24-months or younger-than-24-months or below-24-months or aged-1-to-23-months or aged-one-to-twenty-three-months).tw,kf,hw,dq. 1649049

79 bronchiolitis obliterans/ or (bronchiolitis adj1 obliterans).af. 10869

80 (77 and 78) not 79 20025

81 75 not 79 503

82 80 or 81 20142

83 limit 82 to (english language and embase and yr="2000 -Current") 11881

**Cochrane Library**

Search date: 21/06/23 (search updated February 19, 2025)

#1 MeSH descriptor: [Bronchiolitis] explode all trees

#2 bronchiolit* or wheez* or (Respiratory Syncytial Virus*) or rsv

#3 MeSH descriptor: [Respiratory Syncytial Viruses] explode all trees

#4 MeSH descriptor: [Respiratory Syncytial Virus Infections] explode all trees

#5 MeSH descriptor: [Natural History] explode all trees

#6 MeSH descriptor: [Epidemiology] explode all trees

#7 MeSH descriptor: [Severity of Illness Index] explode all trees

#8 MeSH descriptor: [Diagnosis, Differential] explode all trees

#9 MeSH descriptor: [Physical Examination] explode all trees

#10 MeSH descriptor: [Respiratory Tract Infections] explode all trees

#11 MeSH descriptor: [Risk Factors] explode all trees

#12 MeSH descriptor: [Length of Stay] explode all trees

#13 MeSH descriptor: [Patient Admission] explode all trees

#14 MeSH descriptor: [Intensive Care Units] explode all trees

#15 MeSH descriptor: [Morbidity] explode all trees

#16 MeSH descriptor: [Mortality] explode all trees

#17 MeSH descriptor: [Diagnostic Imaging] explode all trees

#18 MeSH descriptor: [Hematologic Tests] explode all trees

#19 MeSH descriptor: [Urinalysis] explode all trees

#20 MeSH descriptor: [Nasal Lavage Fluid] explode all trees

#21 MeSH descriptor: [Emergency Service, Hospital] explode all trees

#22 MeSH descriptor: [Albuterol] explode all trees

#23 MeSH descriptor: [Epinephrine] explode all trees

#24 MeSH descriptor: [Steroids] explode all trees

#25 MeSH descriptor: [Bronchodilator Agents] explode all trees

#26 MeSH descriptor: [Cholinergic Antagonists] explode all trees

#27 MeSH descriptor: [Anti-Inflammatory Agents] explode all trees

#28 MeSH descriptor: [Adrenal Cortex Hormones] explode all trees

#29 MeSH descriptor: [Leukotriene Antagonists] explode all trees

#30 MeSH descriptor: [Saline Solution, Hypertonic] explode all trees

#31 MeSH descriptor: [Aerosols] explode all trees

#32 MeSH descriptor: [Nebulizers and Vaporizers] explode all trees

#33 MeSH descriptor: [Sodium Chloride] explode all trees

#34 (#31 or #32) and #33

#35 MeSH descriptor: [Oxygen Inhalation Therapy] explode all trees

#36 MeSH descriptor: [Oxygen] explode all trees and with qualifier(s): [administration & dosage - AD, standards - ST]

#37 MeSH descriptor: [Oximetry] explode all trees

#38 MeSH descriptor: [Reproducibility of Results] explode all trees

#39 #37 and #38

#40 MeSH descriptor: [Physical Therapy Modalities] explode all trees

#41 MeSH descriptor: [Physical Therapy Specialty] explode all trees

#42 MeSH descriptor: [Suction] explode all trees

#43 MeSH descriptor: [Fluid Therapy] explode all trees

#44 MeSH descriptor: [Infusions, Intravenous] explode all trees

#45 MeSH descriptor: [Administration, Oral] explode all trees

#46 #43 and (#44 or #45)

#47 MeSH descriptor: [Administration, Intranasal] explode all trees

#48 saline (Word variations have been searched)

#49 #48 and #47

#50 MeSH descriptor: [Rehydration Solutions] explode all trees

#51 MeSH descriptor: [Bacterial Infections] explode all trees

#52 MeSH descriptor: [Otitis Media] explode all trees

#53 MeSH descriptor: [Meningitis] explode all trees

#54 MeSH descriptor: [Anti-Bacterial Agents] explode all trees and with qualifier(s): [therapeutic use - TU]

#55 MeSH descriptor: [Sepsis] explode all trees

#56 MeSH descriptor: [Urinary Tract Infections] explode all trees

#57 MeSH descriptor: [Tracheitis] explode all trees

#58 MeSH descriptor: [Radiography, Thoracic] explode all trees

#59 MeSH descriptor: [Asthma] explode all trees

#60 MeSH descriptor: [Hypersensitivity, Immediate] explode all trees

#61 MeSH descriptor: [Receptors, Adrenergic, beta-2] explode all trees

#62 MeSH descriptor: [Continuous Positive Airway Pressure] explode all trees

#63 MeSH descriptor: [Enteral Nutrition] explode all trees

#64 MeSH descriptor: [Parenteral Nutrition] explode all trees

#65 MeSH descriptor: [Intubation, Gastrointestinal] explode all trees

#66 MeSH descriptor: [Infection Control] explode all trees

#67 MeSH descriptor: [Primary Prevention] explode all trees

#68 MeSH descriptor: [Patient Isolation] explode all trees

#69 (History or Epidemiolog* or "severity of illness" or “disease severity” or scoring system* or diagnosis or physical exam* or auscultation or "blood pressure" or palpation* or percussion or pulse or vital sign* or Respiratory Tract Infection* or risk factor* or "length of stay" or admission or discharge or morbidit* or prevalence or mortalit* or "Diagnostic Imaging" or ((chest or thorac*) and (x-ray* or xray* or “x ray” or radiograph*)) or ((Hematolog* or Haematolog* or blood or virolog* or urine) and (Test or tests or exam* or investigation*)) or Urinalys* or “Nasal Lavage” or ((nose or nasal) and (mucosa or mucus)) or emergency department* or asthma* or atopy or atopic or hypersensitiv*) (Word variations have been searched)

#70 salbutamol or albuterol or ventolin or levalbuterol or adrenalin* or epinephrin* or beta2 adrenergic* or beta2 agonist* or ics or corticosteroid* or cortico steroid*OR cortico-steroid* or glucocorticoid* or gluco corticoid* or gluco-corticoid* or montelukast or Bronchodilat* or steroid or steroids or Cholinergic Antagonist* or cholinergic receptor* or Anti Inflammatory Agent* or Adrenal Cortex Hormone* or Leukotriene Antagonist* or Leukotriene receptor* or "Hypertonic Saline" or (("Sodium Chloride" or saline) and (nebuliz* or nebulis* or vaporiz* or vaporis* or aerosol* or intranasal or "intra nasal" or intra-nasal or nasal)) (Word variations have been searched)

#71 (Oxygen or ((Oximetry or oximeter*) and ("reproducibility of results" or reliability or validity or function* or technical specification* or percutaneous measurement* or blood gas analys*)) or CPAP or “continuous positive airway pressure” or “positive pressure respiration” or “positive end respiratory pressure”) (Word variations have been searched)

#72 Physical Therap* or physiotherap* or ((nasal* or nose or naso) and (suction* or toilet or irrigation)) or suction* or saline drop* or "nasal saline" or "intranasal saline" (Word variations have been searched)

#73 (Fluid Therap* or Intravenous infusion* or “non oral” or Rehydrat* or “enteric feeding” or “parenteral nutrition” or “parenteral feeding” or “enteral nutrition” or oral* administ* or bacterial infection* or "otitis media" or Meningitis or antibacterial agent* or anti bacterial agent* or antimicrobial agent* or anti microbial agent* or antibiotic* or 3z or aruzilina or atizor or azadose or azasite or azatril or azenil or azibiot or azibiot-neo or azimin or azithral or azithromycin or azitrocin or azitromax or azitromicin or azitromicina or aziwok or azomyne or aztrin or azydrop or azyter or azythromycin or bazyt or cp-62933 or cp-62993 or cp62933 or cp62993 or forcin or inedol or infectoazit or isv-401 or isv401 or kromicin or macrozit or mezatrin or octavax or ordipha or ribotrex or sumamed or sunamed or tobyl or tromix or trozocina or ultreon or vinzam or xithrone or xz-450 or xz450 or zaret or zarom or zetamax or zeto or zibramax or zifin or zimericina or zistic or zithromax or zithrox or zitinn or zitrim or zitrobifan or zitrocin or zitromax or zmax or Sepsis or septic or Urinary Tract Infection* or tracheitis or serious bacterial infection* or “infection control” or “primary prevention” or isolation or “patient care”) (Word variations have been searched)

#74 “intensive care” or ICU or "respiratory care" or NICU or PICU

#75 MeSH descriptor: [SARS-CoV-2] this term only

#76 MeSH descriptor: [COVID-19] this term only

#77 ((corona* or corono*) NEAR (virus* or viral* or virinae*)) (Word variations have been searched)

#78 (coronavirus* or coronovirus* or coronavirinae* or CoV or CoV2 or CoV-2 or HCoV*) (Word variations have been searched)

#79 (“2019 nCoV” or 2019nCoV or nCoV2019 or nCoV-2019 or COVID-19 or COVID19 or CORVID-19 or CORVID19 or WNCoV or HCoV-19 or HCoV19 or (2019 NEXT novel*) or Ncov or SARSCoV-2 or SARSCoV2 or SARSCov19 or SARSCov-19 or Ncov or Ncorona* or Ncorono* or NcovWuhan* or NcovHubei* or NcovChina* or NcovChinese* or SARS2 or SARS-2 or SARScoronavirus2 or SARScoronavirus-2 or SARScoronovirus2 or SARScoronovirus-2)

#80 (respiratory* NEAR/2 (symptom* or disease* or illness* or condition*) NEAR/10 (Wuhan* or Hubei* or China* or Chinese* or Huanan*))

#81 ((seafood-market* or food-market* or pneumonia*) NEAR/10 (Wuhan* or Hubei* or China* or Chinese* or Huanan*))

#82 ((outbreak* or wildlife* or pandemic* or epidemic*) NEAR (Wuhan* or Hubei or China* or Chinese* or Huanan*))

#83 (COVID-2019 or COVID2019)

#84 MeSH descriptor: [Post-Acute COVID-19 Syndrome] this term only

#85 (longCOVID* or postCOVID* or postcoronavirus* or postSARS*)

#86 MeSH descriptor: [Antibodies, Monoclonal] explode all trees

#87 (monoclonal-antibod* or clonal-antibod* or hybridoma-antibod* or nirsevimab or medi-8897 or medi8897 or sp-0232 or sp-232 or sp0232 or sp232 or Motavizumab or medi-524 or medi524 or numax or Palivizumab or abbosynagis or medi-493 or medi493 or synagis or synagys)

#88 #5 or #6 or #7 or #8 or #9 or #10 or #11 or #12 or #13 or #14 or #15 or #16 or #17 or #18 or #19 or #20 or #21 or #22 or #23 or #24 or #25 or #26 or #27 or #28 or #29 or #30 or #34 or #35 or #36 or #39 or #40 or #41 or #42 or #46 or #49 or #50 or #51 or #52 or #53 or #54 or #55 or #56 or #57 or #58 or #59 or #60 or #61 or #62 or #63 or #64 or #65 or #66 or #67 or #68 or #69 or #70 or #71 or #72 or #73 or #74 or #75 or #76 or #77 or #78 or #79 or #80 or #81 or #82 or #83 or #84 or #85 or #86 or #87

#89 (#1 or #2 or #3 or #4) and #88

#90 MeSH descriptor: [Pregnant Women] explode all trees

#91 MeSH descriptor: [Pregnancy] explode all trees

#92 MeSH descriptor: [Prenatal Care] this term only

#93 (parturition or ante-natal or antenatal* or pre-natal* or prenatal* or pregnan*)

#94 #90 OR #91 or #92 or #93

#95 MeSH descriptor: [Immunization] explode all trees

#96 (immunis* or immuniz* or vaccin*)

#97 #95 OR #96

#98 Respiratory-syncytial-virus-vaccine* or RSV-vaccine* or Arexvy

#99 (#94 and #97 and (#1 or #2 or #3 or #4)) or (#94 and #98)

#100 (#97 and (#1 or #2 or #3 or #4)) or #98

#101 #89 or #100

#102 (newborn* or new born* or baby or babies or neonat* or neo nat* or infan* or toddler* or “aged 1” or “aged one” or “one year old” or “1 year old” or “under two” or “under 2” or “younger than two” or “younger than 2” or “below two” or “below 2” or “under 24 months” or “younger than 24 months” or “below 24 months” or “aged 1 to 23 months” or “aged one to twenty three months”)

#103 MeSH descriptor: [Bronchiolitis Obliterans] explode all trees

#104 ("bronchiolitis obliterans")

#105 (#101 and #102) not (#103 or #104)

#106 #99 not (#103 or #104)

#107 #105 or #106 with Cochrane Library publication date from Jan 2000 to present

**Results = 2164**

**CINAHL**

Search date 21/06/23 (search updated February 19, 2025)

S20 S18 AND S19

Limiters - English; Published Date: 20000101-; Peer Reviewed (56)

S19 newborn* or new-born* or baby or babies or neonat* or neo-nat* or infan* or toddler* or aged-1 or aged-one or one-year-old or 1-year-old or under-two or under-2 or younger-than-two or younger-than-2 or below-two or below-2 or under-24-months or younger-than-24-months or below-24-months or aged-1-to-23-months or aged-one-to-twenty-three-months (553,820)

S18 S9 AND S17 (114)

S17 S10 OR S11 OR S12 OR S13 OR S14 OR S15 OR S16 (202,865)

S16 (saline W1 drop*) or (nasal W1 saline) (104)

S15 (MH "Administration, Intranasal") and saline (145)

S14 physiotherap* or "physical therap*" (87,362)

S13 (MH "Physical Therapy+") (159,654)

S12 (MH "Suctioning, Nasopharyngeal") OR (MH "Suction") OR (deep W1 suction*) OR ((nasal* or nose or naso) W3 suction*) (2,755)

S11 (MH "Rehabilitation, Pulmonary")

Limiters - Published Date: 20000101-20011231 (106)

S10 (MH "Chest Physiotherapy (Saba CCC)") OR (MH "Chest Physiotherapy (Iowa NIC)") OR (MH "Chest Physical Therapy+") (870)

S9 (s8 not s5) or s6 or s7 (8,657)

S8 S1 OR S2 OR S3 OR S4 (10,610)

S7 (S1 OR S2 OR S3 OR S4) and (MH "Professional Practice, Evidence-Based+") (108)

S6 S1 OR S2 OR S3 OR S4

Limiters - Publication Type: Clinical Trial, Critical Path, Meta Analysis, Practice Guidelines, Randomized Controlled Trial, Review, Systematic Review (1,501)

S5 S1 OR S2 OR S3 OR S4

Limiters - Publication Type: Case Study, Commentary, editorial, Letter (2,006)

S4 bronchiolit* or wheez* or "respiratory syncytial virus*" or rsv (10,438)

S3 (MH "Respiratory Syncytial Virus Infections") OR (MH "Respiratory Syncytial Viruses") (2,772)

S2 (MH "Bronchial Diseases")

Limiters - Published Date: 20000101-20001231 (40)

S1 (MH "Bronchiolitis+") (2,540)

**PubMed**

Search date 21/6/23 (search updated February 19, 2025)

#1 bronchiolitis OR bronchiolitic OR respiratory-syncytial-virus* OR wheez* OR rsv

#2 History OR Epidemiolog* OR "severity of illness" OR "disease severity" OR "scoring system" OR diagnosis OR physical-exam* OR auscultation OR "blood pressure" OR palpation* OR percussion OR pulse OR vital-sign* OR Respiratory-Tract-Infection* OR risk-factor* OR "length of stay" OR admission OR discharge OR "intensive care" OR NICU OR ICU OR PICU OR "respiratory care" OR morbidit* OR prevalence OR mortalit* OR "Diagnostic Imaging" OR ((chest OR thorax OR thoracic) AND (x-ray* OR xray* OR radiograph*)) OR ((Hematolog* OR Haematolog* OR blood OR virolog* OR urine) AND (Test OR tests OR exam* OR investigation*)) OR Urinalys* OR "Nasal Lavage" OR ((nose OR nasal) AND (mucosa OR mucus)) OR emergency-department* OR asthma* OR atopy OR atopic OR hypersensitiv* OR salbutamol OR albuterol OR ventolin OR levalbuterol OR adrenalin* OR epinephrin* OR beta2-adrenergic* OR beta2-agonist* OR ics OR corticosteroid* OR cortico-steroid* OR glucocorticoid* OR gluco corticoid* OR montelukast OR Bronchodilat* OR steroid OR steroids OR Cholinergic-Antagonist* OR cholinergic-receptor* OR Anti-Inflammatory-Agent* OR Adrenal-Cortex-Hormone* OR Leukotriene-Antagonist* OR Leukotriene-receptor* OR "Hypertonic Saline" OR (("Sodium Chloride" OR saline) AND (nebuliz* OR nebulis* OR vaporiz* OR vaporis* OR aerosol* OR intranasal OR intra-nasal OR nasal)) OR Oxygen OR ((Oximetry OR oximeter*) AND ("reproducibility of results" OR reliability OR validity OR function* OR technical-specification* OR percutaneous-measurement* OR blood-gas-analys*)) OR CPAP OR "continuous positive airway pressure" OR "positive pressure respiration" OR "positive end respiratory pressure" OR Physical-Therap* OR physiotherap* OR ((nasal* OR nose OR naso) AND suction*) OR suction* OR saline-drop* OR "nasal saline" OR "nasal toilet" OR "nasal irrigation" OR Fluid-Therap* OR Intravenous-infusion* OR "non oral" OR Rehydrat* OR "enteric feeding" OR "parenteral nutrition" OR "parenteral feeding" OR "enteral nutrition" OR oral* AND administ* OR bacterial-infection* OR "otitis media" OR Meningitis OR antibacterial-agent* OR anti-bacterial-agent* OR antimicrobial-agent* OR anti-microbial-agent* OR antibiotic* OR 3z OR aruzilina OR atizor OR azadose OR azasite OR azatril OR azenil OR azibiot OR azibiot-neo OR azimin OR azithral OR azithromycin OR azitrocin OR azitromax OR azitromicin OR azitromicina OR aziwok OR azomyne OR aztrin OR azydrop OR azyter OR azythromycin OR bazyt OR cp-62933 OR cp-62993 OR cp62933 OR cp62993 OR forcin OR inedol OR infectoazit OR isv-401 OR isv401 OR kromicin OR macrozit OR mezatrin OR octavax OR ordipha OR ribotrex OR sumamed OR sunamed OR tobyl OR tromix OR trozocina OR ultreon OR vinzam OR xithrone OR xz-450 OR xz450 OR zaret OR zarom OR zetamax OR zeto OR zibramax OR zifin OR zimericina OR zistic OR zithromax OR zithrox OR zitinn OR zitrim OR zitrobifan OR zitrocin OR zitromax OR zmax OR Sepsis OR septic OR Urinary-Tract-Infection* OR tracheitis OR serious-bacterial-infection* OR "infection control" OR "primary prevention" OR isolation OR "patient care" OR monoclonal-antibod* OR clonal-antibod* OR hybridoma-antibod* OR nirsevimab OR medi-8897 OR medi8897 OR sp-0232 OR sp-232 OR sp0232 OR sp232 OR Motavizumab OR medi-524 OR medi524 OR numax OR Palivizumab OR abbosynagis OR medi-493 OR medi493 OR synagis OR synagys OR (2019-novel OR 2019nCoV OR 2019-nCoV OR COVID-19 OR COVID19 OR COVID-2019 OR COVID2019 OR CONVID-19 OR CONVID19 OR CORVID-19 OR CORVID19 OR CoV2 OR CoV-2 OR HCoV* OR Ncov* OR Ncorona* OR Ncorono* OR NcovChina* OR NcovChinese* OR NcovHubei* OR NcovWuhan* OR SARS2 OR SARS-2 OR SARScoronavirus2 OR SARScoronavirus-2 OR SARScoronovirus2 OR SARScoronovirus-2 OR SARSCov19 OR SARSCov-19 OR SARS-CoV-2 OR SARSCoV-2 OR SARSCoV2 OR WN-CoV OR WNCoV OR wuhan-virus) OR ((pneumonia* OR outbreak* OR respiratory-illness* OR respiratory-disease* OR respiratory-symptom* OR seafood-market* OR food-market* OR wildlife) AND (Wuhan OR China OR Chinese OR Hubei OR Huanan)) OR ((new OR novel OR nouveau OR 19 OR 2019 OR Wuhan OR Hubei OR Huanan OR China OR Chinese) AND (coronavirus* OR corona virus* OR betacoronavirus* OR CoV OR HCoV)) OR (longCOVID* OR postCOVID* OR postcoronavirus* OR postSARS*) OR ((coronavirus* OR corona-virus* OR betacoronavirus*) AND (pandemic* OR epidemic* OR outbreak* OR crisis))

#3 newborn* OR new-born* OR baby OR babies OR neonat* OR neo-nat* OR infan* OR toddler* OR aged-1 OR aged-one OR one-year-old OR 1-year-old OR under-two OR under-2 OR younger-than-two OR younger-than-2 OR below-two OR below-2 OR under-24-months OR younger-than-24-months OR below-24-months OR aged-1-to-23-months OR aged-one-to-twenty-three-months

#4 NOTNLM

#5 "Bronchiolitis Obliterans"

#6 (#1 AND #2 AND #3 AND #4) NOT #5

Limit 2000 onwards; AND English 2168

[Search name: Cate Wilson 300623 Bronchiolitis AND VOI AND Age AND NLM]

#7 #1 AND #2 AND #3) NOT #5

Limit 2013 onwards; AND English 3576

[Search name: Cate Wilson 300623 Bronchiolitis AND VOI AND Age]

#8 ((bronchiolitis OR bronchiolitic OR respiratory-syncytial-virus* OR wheez* OR rsv) AND (parturition OR ante-natal OR antenatal* OR pre-natal* OR prenatal* OR pregnan*) AND (immunis* OR immuniz* OR vaccin*)) OR ((parturition OR ante-natal OR antenatal* OR pre-natal* OR prenatal* OR pregnan*) AND (Respiratory-syncytial-virus-vaccine* OR RSV-vaccine* OR Arexvy))

#9 (#8 AND #4) NOT #5

Limit 2000 onwards; AND English 160

[Search name: Cate Wilson 300623 Bronchiolitis AND Maternal immunization AND NLM]

#10 #8 NOT #5

Limit 2013 onwards; AND English 271

[Search name: Cate Wilson 300623 Bronchiolitis AND Maternal immunization]

#11 ((bronchiolitis OR bronchiolitic OR respiratory-syncytial-virus* OR wheez* OR rsv) AND (immunis* OR immuniz* OR vaccin*)) OR (Respiratory-syncytial-virus-vaccine* OR RSV-vaccine* OR Arexvy)

#12 (#11 AND #3 AND #4) NOT #5

Limit 2000 onwards; AND English 1087

[Search name: Cate Wilson 300623 Bronchiolitis AND Infant immunization AND Age AND NLM]

#13 (#11 AND #3) NOT #5

Limit 2013 onwards; AND English 1725

[Search name: Cate Wilson 300623 Bronchiolitis AND Infant immunization AND Age]

#14 #6 OR #7 OR #9 OR #10 OR #12 OR #13 4384

## Appendix 4. Study characteristics

### Chest Xray

| **Study design** | **Participants** | **Investigations** | **Methods** | **Outcomes and results** | **Comments** |
| --- | --- | --- | --- | --- | --- |
| **Akande 2024**  Impact of respiratory bacterial codetection on outcomes in ventilated infants with bronchiolitis. The Pediatric Infectious Disease Journal. 2024 Feb 1;43(2):117-22.  **Country**  United States of America  **Study type**  Retrospective observational  **Location of study**  Nationwide Children’s Hospital, Columbus, Ohio, USA  **Study aim**  To examine the association between codetection and outcomes in infants requiring emergency department (ED) intubation for bronchiolitis. | **Sample**  N=149 infants with bronchiolitis admitted to PICU (148 analysed)  **Characteristics**  Median (IQR) age in months: 1.3 (0.75-1.86)  Ethnicity:  White 58%  Black 23%  Other 11%  Hispanic 8%  **Enrolment setting**  ED (with subsequent PICU admission)  **Key inclusion criteria**  Aged <2 years.  Infants were included with viral bronchiolitis requiring endotracheal intubation in the ED between July 2012 – June 2017, and subsequently admitted to PICU.  Exclusion criteria were tracheostomy, admission to a unit other than PICU. | **Test**  Chest radiograph. Results were recorded using dictated radiologist reports into:  a) normal;  b) atelectasis (only);  c) bronchiolitis/ viral (hyperinflation, peribronchial thickening or cuffing, +/- atelectasis);  d) pneumonia;  e) other abnormal findings.  **Comparator**  No comparator. | Single-centre, retrospective cohort study.  Univariate regression. | **ICU length of stay:** Relative risk (RR) with 95% confidence intervals (CI) for longer marginal mean ICU length of stay in days, associated with a normal chest radiograph^1^.  Presence of normal (n=37) vs. abnormal chest radiograph findings (n=112):  RR 0.83 (95% CI 0.69 – 1.01), *p*=.07.  **Primary outcomes:**   - Mechanical ventilation; - ICU length of stay;   Plus secondary outcomes reported in the article. | ^1^Average ICU LOS values not reported by chest radiograph results. The sample median ICU LOS was 6.8 days (IQR 5-10). |
| **Towriss 2025**  Common Radiological Features on Chest X‐Rays of Infants With Bronchiolitis: Do They Support Management?. Acta Paediatrica (Oslo, Norway: 1992). 2025 Jan 30;114(6):1478.  **Country**  United Kingdom  **Study Type**  Observational (retrospective)  **Location of study**  Noah’s Ark Children’s Hospital for Wales, Cardiff, United Kingdom  **Study Aim**  To assess the rate of radiographically defined complicated bronchiolitis, and the rate of alternative radiographic diagnoses in infants with bronchiolitis. | **Sample**  N=431 infants with bronchiolitis and CXR  n=248 pathological result  n=183 benign result  **Characteristics**  Aged under 3 months (%):  Benign: 67%  Pathological: 57%  Ethnicity not reported.  **Enrolment setting**  ED, paediatric ward, PICU  **Key inclusion criteria**  Age criteria: <12 months  Infants admitted to hospital with acute bronchiolitis between 1 October 2015 to 21 March 2023, with CXR.  Exclusion criteria were extreme prematurity (<32wGA), congenital heart disease, lung disease or neurological disability, incomplete discharge advice letters. | **Investigation**  CXR reported by a consultant paediatric radiologist, classified by researchers into benign and pathological based on report.  Benign (‘typical bronchiolitis’): normal, hyperinflation, perihilar infiltrates, diffuse interstitial markings, bronchial wall thickening or linear atelectasis.  Pathological (‘complicated bronchiolitis’): pulmonary infiltrates, collapse, consolidation, airspace opacification, nonlinear atelectasis, pleural effusion, pneumothorax, cardiomegaly, foreign body.  **Comparator**  No allocated comparator (testing was performed on all patients, and comparisons were made by test results). | Retrospective observational study, health record data | **Indicator for administration of antibiotics:** Number of infants (%) who received antibiotics.  Benign vs. pathological CXR:  73/183 (40%) vs. 155/248 (63%), *p*<.001.  **Primary outcomes:**   - Rate of radiographically defined complicated bronchiolitis; - Rate of alternative diagnoses;   Plus other secondary outcomes reported in the article. |  |
| **Williams 2012**  Towards evidence based emergency medicine: best BETs from the Manchester Royal Infirmary. BET 4: Chest x-rays in bronchiolitis. Emergency medicine journal: EMJ. 2012 Jun;29(6):514-5.  **Country**  Canada (2), New Zealand (1), United States of America (1)  **Study type**  Literature review with systematic search  **Location of study**  NA  **Study aim**  To investigate whether chest x-ray (CXR) is a useful investigation to predict severity or alter management in infants with bronchiolitis. | **Sample**  N=1,571 infants with bronchiolitis  N=896 in extracted studies  **Characteristics**  Extracted studies: aged 5.6 (SD 3.1) to 7.5 (SD 5.5) months.  Ethnicity not reported.  **Enrolment setting**  ED, Paediatric ward, ICU  **Key inclusion criteria**  Systematic review: Aged <24 months.  Extracted studies: Aged <12 months to <22 months.  Eligibility criteria for the review not reported. | **Tests**  CXR (any use)  **Comparator**  No comparator | Literature review with systematic search, including 8 observational studies.  4 studies were eligible and extracted^1^. 3 prospective cohort, 1 retrospective cohort study.  Databases searched: MEDLINE, EMBASE.  Date of last search: March 2012 | **Diagnostic accuracy:** a) Atelectasis on CXR as a predictor of severe disease, as rated ‘mild’ or ‘severe’ by radiologists (Shaw et al. 1991).  OR 2.7 (95% CI 0.97 to 3.70)  Se 21%  Sp 98%  PPV 82%  NPV 70%  b) Association between clinical severity grading and radiological changes of hyperinflation, infiltration, and atelectasis (Dawson et al. 1990).  No significant relationship between clinical severity ratings and the degree of hyperinflation (X^2^=9.92 (df 9), *p*<.10), degree of infiltration (X^2^=4.56 (df 12), *p*<.10), or a summed grading score indicative of the degree of hyperinflation, infiltration, and atelectasis (X^2^=6.55 (df 6), *p*<.10).  **Indicator for administration of antibiotics:** Rate of antibiotic prescription (Schuh et al. 2007).  Pre vs. post CXR (n=265):  7 of 265 (2%) vs. 39 of 265 (14.7%).  95% CI for difference in agreement 0.08 to 0.16.  **Cost-effectiveness:** Cost savings per patient associated with omitting CXR (2005 CAD) (Yong et al. 2009).  CAD$59.09 per patient cost-saving.  **Primary outcomes:**   - Disease severity;   Plus secondary outcomes reported in the article. | ^1^ The remaining studies did not meet our eligibility criteria.  Extracted from eligible studies:  -Dawson et al., 1990;  -Shaw et al., 1991 ;  -Schuh et al., 2007  -Yong et al., 2009.  Informal risk of bias evaluations were reported per study, including selection bias (Dawson et al., 1990; Schuh et al., 2007), lack of blinding to result (Shaw et al., 1991). |
| **Wrotek 2019**  Chest radiography in children hospitalized with bronchiolitis. In: Pokorski, M. (eds) Pulmonology. Advances in Experimental Medicine and Biology, vol 1222. Springer, Cham.    **Country**  Poland  **Study Type**  Observational (retrospective)  **Location of study**  Bielanski Hospital, Warsaw, Poland  **Study Aim**  To evaluate the use of CXR in children with bronchiolitis. | **Sample**  N=581 children  n=459 infants with bronchiolitis (n=390 with RSV)  n=65 children with RSV pneumonia  n=57 children with RSV bronchitis  **Characteristics**  Median (IQR) age in months:  With CXR: 3.0 (1.5 to 5.9)  No CXR: 2.4 (1.5 to 4.0).  Ethnicity not reported.  **Enrolment setting**  ED, paediatric ward  **Key inclusion criteria**  Age criteria: not reported.  Eligibility criteria not reported. | **Investigation**  CXR performed, divided into CXR positive and CXR negative subgroups.  **Comparator**  No CXR control | Retrospective observational study | **Indicator for administration of antibiotics:** Odds ratio (OR; 95% CI) for administration of antibiotic treatment.  CXR (n=166) vs. no CXR (n=415):  22.9 fold (95% CI 14.1 to 37.1) greater risk of antibiotic treatment with receipt of CXR (any results) compared to no CXR (*p*<.01).  Positive CXR vs. negative CXR:  4.4 fold (95% CI 2.2 to 8.9) greater risk of antibiotic treatment with positive CXR compared to negative CXR (*p*<.01).  **Primary outcomes:**   - Frequency of CXR;   Plus other secondary outcomes reported in the article. |  |

### Laboratory tests

| **Study design** | **Participants** | **Investigations** | **Methods** | **Outcomes and results** | **Comments** |
| --- | --- | --- | --- | --- | --- |
| **Akande 2024**  Impact of respiratory bacterial codetection on outcomes in ventilated infants with bronchiolitis. The Pediatric Infectious Disease Journal. 2024 Feb 1;43(2):117-22.  **Country**  United States of America  **Study type**  Retrospective observational  **Location of study**  Nationwide Children’s Hospital, Columbus, Ohio, USA  **Study aim**  To examine the association between codetection and outcomes in infants requiring emergency department (ED) intubation for bronchiolitis. | **Sample**  N=149 infants with bronchiolitis admitted to PICU (148 analysed)  **Characteristics**  Median (IQR) age in months: 1.3 (0.75-1.86)  Ethnicity:  White 58%  Black 23%  Other 11%  Hispanic 8%  **Enrolment setting**  ED (with subsequent PICU admission)  **Key inclusion criteria**  Aged <2 years.  Infants were included with viral bronchiolitis requiring endotracheal intubation in the ED between July 2012 – June 2017, and subsequently admitted to PICU.  Exclusion criteria were tracheostomy, admission to a unit other than PICU. | **Test**  Lower respiratory cultures, obtained via endotracheal tube aspirate using new sterile catheters. The following were analysed:  a. presence of codetection;  b. presence of moderate/ many polymorphonuclear neutrophils (PMNs);  c. presence of positive culture.  Codetection was defined as a positive respiratory culture and moderate or many PMNs on Gram stain. A positive culture was defined as growth of any pathogenic bacteria.  **Comparator**  No comparator. | Single-centre, retrospective cohort study.  Univariate and multivariate regression, adjusted for significant variables in univariate analyses (presence of prematurity, RSV positive status, any other positive culture). | **ICU length of stay:** adjusted relative risk (Adj RR) with 95% confidence intervals (CI) for longer marginal mean ICU length of stay in days.  Codetection (n=77) vs. no codetection (n=72):  6.9 days (95% CI 6.21-7.68) vs. 8.57 days (7.68–9.56)  Adj RR 0.81 (95% CI 0.69–0.94), *p*<.01.  **Primary outcomes:**   - Mechanical ventilation; - ICU length of stay;   Plus secondary outcomes reported in the article. |  |
| **Al Shibli 2017**  Significance of platelet count in children admitted with bronchiolitis. World Journal of Clinical Pediatrics. 2017 May 8;6(2):118.    **Country**  United Arab Emirates  **Study Type**  Observational (retrospective)  **Location of study**  Tawam Hospital, Abu Dhabi, United Arab Emirates  **Study Aim**  To determine the true prevalence of thrombocytosis in children <2 years of age with bronchiolitis, its association with risk factors, disease severity, and thromboembolic complications. | **Sample**  N=305 infants hospitalised for bronchiolitis  **Characteristics**  Mean age (SD) in months: 6.5 (0.2)  Ethnicity not reported.  **Enrolment setting**  Paediatric ward  **Key inclusion criteria**  Age criteria: ≤2 years  Infants admitted to hospital with acute bronchiolitis between 1 November 2008 to 30 June 2012.  Exclusion criteria were presence of bacterial co-infection, chronic disease, immune deficiencies, splenectomy, congenital cyanotic heart disease with polycythemia, presence of intravascular lines, treatment with medications associated with thrombocytosis, personal or family history of thrombophilia. | **Investigation**  Thrombocytosis (platelet count of >500 x 10^9^/L), from first platelet count taken at admission. Categorised into none, mild, moderate, and severe.  **Comparator**  No allocated comparator (testing was performed on all patients, and comparisons were made by test results). | Retrospective observational medical chart review | **Length of stay:** Mean (SD) length of hospital stay in days.  Thrombocytosis none (n=217) vs. mild (n=78) vs. moderate (n=9) vs. severe (n=1):  4.0 (5.5) vs. 4.4 (4) vs. 4.5 (3) vs. 5.0 (0), *p*=.9.  **Death:** Number of deaths.  Thrombocytosis none vs. mild vs. moderate vs. severe:  0/217 (0%) vs. 0/78 (0%) vs. 0/9 (0%) vs. 0/1 (0%).  **Primary outcomes:**   - Prevalence;   Plus other secondary outcomes reported in the article. |  |
| **Alejandre 2021**  Use of procalcitonin and C-reactive protein in the diagnosis of bacterial infection in infants with severe bronchiolitis. European Journal of Pediatrics. 2021 Mar;180:833-42.  **Country**  Spain  **Study type**  Prospective observational  **Location of study**  Hospital Sant Joan de Deu, University of Barcelona, Barcelona, Spain  **Study aim**  To evaluate the use of procalcitonin (PCT) and C-reactive protein (CRP) for the diagnosis of bacterial infection in infants with severe bronchiolitis. | **Sample**  N=675 infants with severe acute bronchiolitis  n=181 patients with invasive bacterial infection (IBI)  (n=72 (10.7%) had sepsis  n=106 (15.7%) had pneumonia  n=41 (6.1%) had a UTI)  n=494 patients with no bacterial infection (NBI)  **Characteristics**  Median age in days (IQR): 47 days (25 to 100.3).  Ethnicity not reported.  **Enrolment setting**  PICU  **Key inclusion criteria**  Age criteria not reported.  Patients with severe acute bronchiolitis who were admitted to PICU between January 2011 to July 2017.  Excluded patients <7 days old, infants who received antibiotic treatment before PICU admission, infants with chronic pathology that may affect CRP values, infants who had surgery before or after the hospitalisation. | **Tests**  1) Serum PCT  2) CRP  Blood samples were collected at PICU admission, and at 24 and 48 hours post admission.  **Comparator**  No allocated comparator (all participants received the same testing, and were compared by the results). | Prospective observational study, single-centre | **Diagnosis of bacterial co-infection:**  1) Best PCT cut-off points for invasive bacterial infection (IBI) diagnosis:  a) At PICU admission:  1.4 ng/mL, Sn 69% (95% CI 58.4 to 74.9), Sp 91% (95% CI 88.1 to 92.5), positive predictive value 76.7% (95% CI 70.2 to 83.1), negative predictive value 86.2% (95% CI 83.1 to 89.3).  b) 24 hours after PICU admission:  3.3 ng/mL, Sn 56% (95% CI 48.5 to 64.5), Sp 90.7% (95% CI 84.9 to 93.6%), positive predictive value 81.9% (95% CI 72.1 to 87.2), negative predictive value 74.3% (95% CI 68.2 to 79.1).  2) Best CRP cut-off points for IBI diagnosis:  a) At PICU admission:  26 mg/dL, Sn 62.7%, Sp 71.9% (95% CIs, PPV, NPV not reported)  b) 24 hours after PICU admission:  38 mg/dL, Sn 72.9%, Sp 64.1% (95% CIs, PPV, NPV not reported)  3) Area under the curve (AUC) (95% CI) for diagnosing IBI according to the values of PCT and CRP at admission, 24h, and 48h.  At PICU admission:  PCT: 0.835 (0.792 to 0.878)  CRP: 0.716 (0.66 to 0.76)  After 24 hours:  PCT: 0.833 (0.765 to 0.902)  CRP: 0.718 (0.638 to 0.798)  After 48 hours:  PCT: 0.670 (0.570 to 0.771)  CRP: 0.630 (0.526 to 0.733)  4) AUC (95% CI) for diagnosing sepsis.  At PICU admission:  PCT: 0.91 (0.87 to 0.95)  CRP: 0.73 (0.66 to 0.8)  After 24 hours:  PCT: 0.89 (0.92 to 0.95)  CRP: 0.73 (0.64 to 0.82)  After 48 hours:  PCT: 0.81 (0.71 to 0.91)  CRP: 0.64 (0.50 to 0.78)  5) AUC (95% CI) for diagnosing pneumonia.  At PICU admission:  PCT: 0.82 (0.77 to 0.87)  CRP: 0.77 (0.72 to 0.83)  After 24 hours:  PCT: 0.81 (0.74 to 0.88)  CRP: 0.71 (0.62 to 0.8)  After 48 hours:  PCT: 0.64 (0.52 to 0.75)  CRP: 0.65 (0.53 to 0.76)  6) AUC (95% CI) for diagnosing UTI not reported.  **Primary outcomes:**   - Diagnosis of invasive bacterial infection;   Plus secondary outcomes reported in the article. |  |
| **Burrack 2023**  Monocyte and neutrophil to lymphocyte ratios in hospitalized children with RSV bronchiolitis. Pediatric Pulmonology. 2023 Dec;58(12):3530-41.  **Country**  Israel  **Study type**  Retrospective cohort  **Location of study**  Soroka University Medical Center, Israel  **Study aim**  To assess the value of MLR and NLR as biomarkers of severe clinical outcomes in children hospitalised with RSV-bronchiolitis. | **Sample**  N=2,038 infants hospitalised with RSV-bronchiolitis.  N=1,934 ward patients  n=104 PICU patients  **Characteristics**  Median age in months (IQR): 4.4 months (IQR 1.9 to 9.8).  Ethnicity:  Arab 62.5%  Jewish 37.5%  **Enrolment setting**  Paediatric hospital ward, PICU  **Key inclusion criteria**  Age ≤24 months.  Infants were included who were admitted to hospital for acute RSV-bronchiolitis between January 2018 and March 2022, who had a complete blood count upon admission. RSV was confirmed through a positive molecular reverse transcription polymerase chain reaction (RT-PCR) test from nasal swabs up to 7 days after admission. Included patients were admitted for their first hospitalisation during the study period.  Patients were excluded with a severe medical condition, including genetic and chromosomal abnormalities, CHD, neuromuscular impairments. | **Test**  Monocyte-to-lymphocyte ratio (MLR): absolute monocyte count/absolute lymphocyte count.  Neutrophil-to-lymphocyte ratio (NLR): absolute neutrophil count/absolute lymphocyte count.  Collected from routine complete blood counts upon hospital admission.  **Comparator**  No allocated comparator (all participants received a complete blood count, and were compared by MLR and NLR quartiles). | Retrospective cohort study, single-centre.  Data were collected via electronic medical records and the hospital’s medical informatics unit.  The cohort was divided into quartiles based on MLR and NLR scores (Q1 to Q4).  Multivariable analysis adjusted for age and sex, for outcomes of prolonged length of stay (admission longer than 75% percentile; 4.2 days), and diagnosis of bacterial co-infection (pneumonia). | **Length of stay:** Mean (SD) length of stay in days. Adjusted relative risk (Adj RR; 95% confidence interval) for prolonged length of stay.  1) MLR:  a) Q1 (n=510) vs. Q2 (n=510) vs. Q3 (n=509) vs. Q4 (n=509):  3.1 (3.0) vs. 3.2 (2.4) vs. 3.7 (3.2) vs. 4.1 (3.3), *p*<.001.  b) i) Q4 vs. Q1: Adj RR 1.6 (95% CI 1.3 to 2.0), *p*<.001.  ii) Q3 vs. Q1: Adj RR 1.4 (95% CI 1.1 to 1.8), *p*=.003.  iii) Q2 vs. Q1: Adj RR 1.0 (95% CI 0.8 to 1.3), *p*=.9.  2) NLR:  a) Q1 (n=510) vs. Q2 (n=510) vs. Q3 (n=509) vs. Q4 (n=509):  3.3 (2.7) vs. 2.2 (3.0) vs. 3.6 (2.8) vs. 3.8 (3.6), *p*=.03.  b) i) Q4 vs. Q1: Adj RR 1.7 (95% CI 1.4 to 2.2), *p*<.001.  ii) Q3 vs. Q1: Adj RR 1.5 (95% CI 1.2 to 1.8), *p*<.001.  iii) Q2 vs. Q1: Adj RR 1.1 (95% CI 0.9 to 1.4), *p*=.5.  **Length of ICU stay:** Mean (SD) length of stay in PICU in days.  1) MLR Q1 (n=12) vs. Q2 (n=20) vs. Q3 (n=24) vs. Q4 (n=48):  4.9 (SD 5.9) vs. 3.8 (SD 1.8) vs. 4.8 (SD 4.2) vs. 5.5 (SD 4.8), *p*=.6.  2) NLR Q1 (n=26) vs. Q2 (n=22) vs. Q3 (n=29) vs. Q4 (n=27):  5.3 (SD 5.0) vs. 4.0 (SD 3.3) vs. 4.6 (SD 2.7) vs. 5.7 (SD 5.7), *p*=.8.  **Diagnosis of bacterial co-infection:** Number of infants (%) with a diagnosis of bacterial co-infection (pneumonia, UTI).  1) MLR:  Q1 (n=510) vs. Q2 (n=510) vs. Q3 (n=509) vs. Q4 (n=509):  i) Bacteremia: 0 (0.0%) vs. 3 (0.6%) vs. 4 (0.8%) vs. 1 (0.2%), *p*=.13.  ii) Pneumonia: 34 (6.7%) vs. 33 (6.5%) vs. 30 (5.9%) vs. 34 (6.7%), *p*>.9.  iii) UTI: 2 (0.4%) vs. 6 (1.2%) vs. 6 (1.2%) vs. 8 (1.6%), *p*=.3.  2) NLR:  a) Q1 (n=510) vs. Q2 (n=510) vs. Q3 (n=509) vs. Q4 (n=509):  i) Bacteremia: 2 (0.4%) vs. 3 (0.6%) vs. 1 (0.2%) vs. 2 (0.4%), *p*>.9.  ii) Pneumonia: 19 (3.7%) vs. 29 (5.7%) vs. 34 (6.7%) vs. 49 (9.6%), *p*=.002.  iii) UTI: 1 (0.2%) vs. 6 (1.2%) vs. 6 (1.2%) vs. 9 (1.8%), *p*=.11.  **Primary outcomes:**   - PICU admission; - Extended length of stay; - Low minimal saturation; - Diagnosis of secondary pneumonia;   Plus secondary outcomes reported in the article. |  |
| **Cebey-Lopez 2016**  Bacteraemia in children hospitalized with respiratory syncytial virus infection. PLoS One. 2016 Feb 12;11(2):e0146599.  **Country**  Spain  **Study type**  Prospective observational  **Location of study**  13 Spanish tertiary hospitals (GENDRES network), Spain  **Study aim**  To determine the rate and predictive factors of bacteraemia in children hospitalised with confirmed RSV acute respiratory illness. | **Sample**  N=66 infants with RSV-positive acute respiratory illness (78.8% with bronchiolitis)  n=28 PICU patients  n=7 PICU patients with a positive PCR and bronchiolitis  **Characteristics**  Age <12 months: 92.4% of sample.  7 of 7 (100%) of PICU patients with a positive PCR.  Ethnicity not reported.  **Enrolment setting**  Hospital ward, PICU  **Key inclusion criteria**  Age ≤24 months.  Previously healthy infants admitted to a GENDRES network hospital with confirmed RSV infection between 2011-2013.  Patients were excluded with a known history of immunodeficiency, severe cardiac anomaly, severe CLD, neuromuscular disease, chromosomal abnormalities or any ongoing chronic disease. | **Test**  Nested PCR to assess for 19 viruses including RSV, and the presence of 10 different organisms.  PCR were performed using samples obtained after patients were transferred to PICU and recruited into the study.  Participants categorised by positive and negative blood bacterial PCR (with presence of Bacteremia defined as a positive PCR and/or blood culture).  **Comparator**  No allocated comparator (all participants received the same testing). | Prospective observational sub-study, multicentre. | **Length of stay:** Mean (SD) length of hospital stay in days.  PCR confirmed bacteremia vs. negative:  12.1 (4.3) vs. 7.5 (4.7), *p*=.007.  **Diagnosis of bacterial co-infection:** Number of PICU patients with positive PCR (bacteremia):  7 of 28 (25%)  Number of patients with a positive blood bacterial PCR:  1 of 7 (14.3%)  **Primary outcomes:**   - Rate of bacteraemia; - Predictive factors of bacteraemia;   Plus secondary outcomes reported in the article. | The article reports that only 14.3% of PCR positive patients had been vaccinated against pneumococcal disease, whereas 52.5% of the PCR negative patients had received the vaccine. |
| **Celik 2020**  Neutrophil responses in infants with acute bronchiolitis. Ann Clin Anal Med. 2020;11(04):277-82.  **Country**  Turkey  **Study Type**  Observational (prospective)  **Location of study**  Hospital not reported; Aydin, Turkey  **Study Aim**  To evaluate neutrophil counts and to explore its clinical significance in infants with acute bronchiolitis. | **Sample**  N=146 infants  n=94 hospitalised with acute bronchiolitis (n=47 RSV, n=47 rhinovirus (RV))  n=52 healthy infant controls who presented to a child health outpatient clinic  **Characteristics**  Median age in days (IQR): 82 (52.2-180).  Ethnicity not reported.  **Enrolment setting**  Paediatric ward, outpatient clinic  **Key inclusion criteria**  Age criteria: <12 months  Infants admitted to hospital with acute bronchiolitis, or healthy infants who presented to a child health outpatient clinic.  Exclusion criteria were hospitalisation in prior 2 weeks before current admission, prematurity, bronchopulmonary dysplasia, congenital heart disease, immunodeficiency, chronic lung disease. | **Investigation**  Complete blood count with assessment of neutrophil-to-lymphocyte ratios (NLR).  **Comparator**  No allocated comparator (testing was performed on all patients, and comparisons were made by test results). | Observational cohort study, prospective | **Length of stay:** Cut-off value for NLR to predict a duration of hospitalisation ≥9 days in infants with acute bronchiolitis.  NLR cut-off value >1.04:  AUC 0.675 (95% CI 0.548 – 0.802), Se 44.4%, Sp 81%, *p*=.022.  **Primary outcomes:**   - Clinical significance of complete blood count parameters;   Plus other secondary outcomes reported in the article. |  |
| **Erdede 2023**  Neutrophil-to-lymphocyte ratio and the systemic Immune-inflammation index: biomarkers in infants with bronchiolitis: a cross-sectional study. Japanese Journal of Infectious Diseases. 2023 Nov 30;76(6):351-7.    **Country**  Turkey  **Study Type**  Observational (retrospective)  **Location of study**  Zeynep Kamil Maternity and Children’s Disease Training and Research Hospital, Istanbul, Turkey  **Study Aim**  To evaluate the use of systemic immune-inflammation index and neutrophil-to-lymphocyte ratios in infants with bronchiolitis, and to determine whether they could predict bronchiolitis with RSV alone or co-infection with other viruses, and disease severity. | **Sample**  N=155 infants hospitalised for bronchiolitis  **Characteristics**  Mean (SD) age in months: 5.9 (5.5).  Ethnicity not reported.  **Enrolment setting**  Paediatric ward  **Key inclusion criteria**  Age criteria: <24 months  Infants admitted to hospital with moderate or severe bronchiolitis caused by RSV and/or other viral pathogens between 1 October 2021 to 31 January 2022, with first episode of wheezing. Illness severity determined by scoring system (Gadjos).  Exclusion criteria were presence of comorbidity, such as primary immunodeficiency, neuromuscular disease, chronic lung and heart disease. Presence of pneumonia confirmed by chest radiography, or SARS-CoV-2 infection. PICU admission or need for mechanical ventilation. | **Investigation**  Laboratory testing performed on first day of admission:  a) Neutrophil-to-lymphocyte ratios (NLR)  b) Systemic immune-inflammation index (SII): platelet count x NLR.  **Comparator**  No allocated comparator (testing was performed on all patients, and comparisons were made by test results). | Single centre retrospective observational study using electronic health records | **Length of stay:** Mean length of stay in days.  SII values:  r= -0.03, *p*=.66.  **Primary outcomes:**   - Disease severity score;   Plus other secondary outcomes reported in the article. | Only LOS results for SII were reported. Mean LOS not reported. |
| **Fares 2011**  The use of C-reactive protein in predicting bacterial co-Infection in children with bronchiolitis. North American journal of medical sciences. 2011 Mar;3(3):152.    **Country**  Lebanon  **Study Type**  Observational (prospective)  **Location of study**  Makassed General Hospital, Beirut, Lebanon  **Study Aim**  To evaluate the use of C-reactive protein in predicting bacterial co-infection in patients hospitalised for bronchiolitis, and to correlate the results with use of antibiotics. | **Sample**  N=49 infants hospitalised with bronchiolitis  **Characteristics**  Mean (SD) age in days:  Positive culture: 107 (80)  Negative culture: 128 (114)  Ethnicity not reported.  **Enrolment setting**  Paediatric ward  **Key inclusion criteria**  Age criteria: <24 months  Infants admitted to hospital with bronchiolitis from October 2008 to April 2009.  Exclusion criteria were presence of pneumonia from chest radiograph, evidence of other associated infections (acute otitis media, acute gastroenteritis), underlying chronic lung disease, use of antibiotics within 2 weeks prior to admission, systemic disease, previous exposure to mechanical ventilation, malnutrition, immunocompromised patients, two morphotypes from tracheal aspirate culture. | **Investigation**  Tracheal aspirate culture collected at hospital admission, assessed for a variety of organisms including haemophilus influenzae, Moraxella catarrhalis, Escherichia coli, staphylococcus aureus, streptococcus pneumoniae, among others. Results classified into positive and negative cultures.  C-reactive protein (CRP) from blood sample collected at hospital admission. CRP >0.3 mg/dL was considered positive (however different cut-offs are reported in results).  **Comparator**  No allocated comparator (testing was performed on all patients, and comparisons were made by test results). | Prospective observational study, comparing groups with positive and negative tracheal aspirate cultures. | **Length of stay:** Mean (SD) length of stay in days.  Positive culture (n=29) vs. negative culture (n=20):  7.60 (2.38) vs. 4.50 (0.71), *p*=NS^1^.  **Diagnosis of bacterial co-infection:** number of infants with definite bacterial co-infection (CRP ≥2 mg/dL).  Positive culture vs. negative culture:  5/29 (17.2%) vs. 0/20 (0%), *p*=NR.  **Primary outcomes:**   - Presence of bacterial co-infection;   Plus other secondary outcomes reported in the article. | ^1^Value not reported. |
| **Gaurav 2024**  Hyponatremia as early predictor for bronchiolitis grading severity in children admitted to intensive care unit. International Journal of Pharmaceutical and Clinical Research. 2024; 16(6):971-975.  **Country**  India  **Study type**  Prospective observational  **Location of study**  Hospital name not reported; Pawapuri, Nalanda, India.  **Study aim**  To study the association of hyponatremia and bronchiolitis severity and its clinical outcome. | **Sample**  N=60 infants admitted to PICU with bronchiolitis.  **Characteristics**  Age <12 months: 91.7%  Ethnicity not reported.  **Enrolment setting**  PICU  **Key inclusion criteria**  Aged 2 months – 2 years.  Infants were included who were admitted to PICU with a diagnosis of bronchiolitis within 18 hours of hospitalisation.  Infants were excluded who were referral cases treated outside of the hospital, and/or with hypernatremia (serum sodium >145 mEq/l), congenital heart disease, neuromuscular disorders, chronic lung disease. | **Test**  Serum sodium status within 18 hours of admission, from a 3ml serum sample. Results were categorised into:  a. normonatremic (serum sodium of 135-145 mEq/L);  b. hyponatremic (serum sodium <135 mEq/L).  **Comparator**  No comparator. | Prospective cohort study, convenience sampling. | **Death:** number (%) of deaths per group.  Hyponatremia vs. normonatremia:  0/21 (0.0%) vs. 0/39 (0.0%).  **ICU length of stay:** number (%) of infants whose duration of ICU stay was <2 days, 2-4 days, or >4 days duration.  Hyponatremia (n=21) vs. normonatremia (n=39):  <2 days: 13 vs. 25  2-4 days: 7 vs. 12  >4 days: 1 vs. 2  **Primary outcomes:**   - Mechanical ventilation use; - ICU length of stay;   Plus secondary outcomes reported in the article. | Study reports having collected length of stay data; results are not reported. |
| **Laham 2014**  Procalcitonin to predict bacterial coinfection in infants with acute bronchiolitis: a preliminary analysis. Pediatric emergency care. 2014 Jan 1;30(1):11-5.  **Country**  United States of America  **Study type**  Retrospective observational  **Location of study**  University of Kentucky Chandler Medical Centre, Lexington, KY, USA.  **Study aim**  To conduct a preliminary analysis of serum procalcitonin (PCT) to predict bacterial co-infection in infants with acute bronchiolitis. | **Sample**  N=40 infants with bronchiolitis  **Characteristics**  Mean age in months: 2 (SD not reported)  Ethnicity not reported.  **Enrolment setting**  PICU  **Key inclusion criteria**  Age criteria not reported.  Included were infants with a primary diagnosis of acute bronchiolitis at the time of PICU admission, and a PCT assay obtained at admission. | **Tests**   1. Serum PCT 2. White blood cell count (WBC) 3. Bacterial cultures (blood, cerebrospinal fluid, tracheal aspirate, urine)   **Comparator**  No comparator | Retrospective cohort study of electronic medical records. | **Diagnosis of bacterial co-infection:** a) Number of patients diagnosed with bacterial co-infection from bacterial cultures.  Any co-infection: 9 of 40 (22.5%).  Bacterial pneumonia: 8 of 18 (44.4%) tested.  Urinary tract infection: 2 of 33 (6.1%) tested.  Bacteraemia: 0 of 36 (0.0%) tested.  Meningitis: 0 of 12 (0.0%) tested.  b) Performance of PCT versus WBC at predicting a diagnosis of bacterial co-infection.  PCT (>1.5 ng/mL cut-off value):  AUC 0.88, Se 0.80, Sp 1.00. Serum PCT was significantly associated with bacterial co-infection (*p*<.0001).  WBC (<6400/µL cut-off value):  AUC 0.67, Se 0.33, Sp 0.96, *p*=.06.  **Primary outcomes:**   - Diagnosis of bacterial co-infection;   Plus secondary outcomes reported in the article. |  |
| **Matera 2022**  Low lymphocyte count: A clinical severity marker in infants with bronchiolitis. Pediatric Pulmonology. 2022 Jul;57(7):1770-5.    **Country**  Italy  **Study Type**  Observational (retrospective)  **Location of study**  Department of Maternal Infantile and Urological Sciences, Sapienza University of Rome, Rome, Italy.  **Study Aim**  To evaluate the correlation between lymphocyte count and clinical manifestation in infants hospitalised with bronchiolitis. | **Sample**  N=1,297 infants hospitalised for bronchiolitis.  **Characteristics**  Median (IQR) age in days:  84.4 (60.8).  Ethnicity not reported.  **Enrolment setting**  Paediatric ward, ICU  **Key inclusion criteria**  Age criteria: <12 months  Infants admitted to hospital with bronchiolitis from 2004 to 2019, full-term.  Exclusion criteria were presence of underlying chronic diseases (such as cystic fibrosis, chronic neuromuscular diseases, immunodeficiencies, congenital heart disease, chronic lung disease), prematurity (born <37wGA). | **Investigation**  Lymphocyte count from blood sample collected at hospital admission. Divided into three groups by lymphocyte count tertiles:  a) G1: 2914.2 (SD 745.5) /mm^3^  b) G2: 4897.6 (SD 561.5) /mm^3^  c) G3: 7884.3 (SD 1903.3) /mm^3^  **Comparator**  No allocated comparator (testing was performed on all patients, and comparisons were made by test results). | Retrospective cohort study with annual follow up over five years. | **Length of stay:** Mean (SD) length of hospitalisation in days.  G1 (n=433) vs. G2 (n=432) vs. G3 (n=432):  5.8 (3.3) vs. 5.1 (3.3) vs. 5.0 (2.3), *p*<.001.  **Primary outcomes:**   - NA;   Plus other secondary outcomes reported in the article. |  |
| **McDaniel 2019**  Association of diagnostic criteria with urinary tract infection prevalence in bronchiolitis: a systematic review and meta-analysis. JAMA pediatrics. 2019 Mar 1;173(3):269-77.  **Country**  United States of America  **Study Type**  Systematic review and meta-analysis of prevalence studies  **Location of included studies**  Not reported  **Study Aim**  To examine the prevalence of UTI in infants with bronchiolitis when positive urinalysis results are included in the UTI definition. | **Sample**  N=7,453 infants diagnosed with bronchiolitis  **Characteristics**  Mean age not reported. The average sample age in all 18 studies was <12m.  Ethnicity not reported.  **Enrolment setting**  ED, ward, ICU, outpatient  **Key inclusion criteria**  Age criteria: <24 months for the systematic review. The age criteria of the included studies ranged from infants <60 days to <24 months.  Studies were included that reported a UTI prevalence rate in infants with a primary diagnosis of bronchiolitis. | **Test**  Urinalysis test for diagnosing UTI. | Systematic review of 18 UTI prevalence studies.  The included studies were:  4 prospective cohort  11 retrospective medical record review  1 retrospective cohort  1 prospective cross-sectional  1 prospective observational  7 studies were included in the meta-analysis.  Databases searched: MEDLINE, EMBASE.  Date of last search: September 2017 | **Diagnosis of bacterial co-infection:** Sensitivity, specificity, and AUC were not reported. Rather, a narrative synthesis is provided on varying definitions of a positive urinalysis test result.  Definitions of a positive urinalysis test result varied between studies.  The most common definition was at least 10,000 cfu/mL of a single pathogen on a catheterized specimen.  Others accepted any growth of a known pathogen as a positive result.  Three studies did not define what a positive UA result entailed.  **Primary outcomes:**   - Definitions of UTI in bronchiolitis; - Prevalence of concomitant UTI in bronchiolitis;   Plus secondary outcomes reported in the article. | The included articles were rated 5 to 7 out of 7 for quality from an adapted version of the JBI Checklist for Prevalence Studies. |
| **Mitri 2021**  Blood eosinophils, specific immunoglobulin E, and bronchiolitis severity. Pediatric pulmonology. 2021 Sep;56(9):2997-3004.    **Country**  United States of America  **Study Type**  Observational (prospective)  **Location of study**  17 hospitals (14 states, USA)  **Study Aim**  To investigate the association between blood eosinophilia and severity, sIgE sensitization and severity, and interation between the two biomarkers on severity in infants hospitalised with bronchiolitis. | **Sample**  N=1,016 infants hospitalised for bronchiolitis (n=873 analysed)  **Characteristics**  Median (IQR) age in months: 3.0 (NR).  Ethnicity:  Normal:  Non-hispanic white 43%  Non-hispanic black 24%  Hispanic 28%  High:  Non-hispanic white 46%  Non-hispanic black 21%  Hispanic 31%  **Enrolment setting**  Paediatric ward  **Key inclusion criteria**  Age criteria: <12 months  Infants admitted to hospital with bronchiolitis between 2011-2014 fall/winter seasons.  Exclusion criteria were transfer to a participating hospital >48 hours after original admission, >24 hours since transfer to a participating hospital, presence of certain chronic conditions (immunodeficiency, known heart-lung disease), gestational age <32 weeks. | **Investigation**  Blood eosinophil values measured at enrolment (during pre-admission visit or hospitalisation), categorised into normal (<3%) and high (≥3%).  **Comparator**  No allocated comparator (testing was performed on all included patients, and comparisons were made by test results). | Secondary analysis of a multicentre, prospective cohort study (MARC-35), with multivariable logistic regression, adjusted for age, sex, median household income (ZIP code), prematurity, history of eczema, days since problem breathing began, presence of apnoea, oxygen saturation, and potential clustering by hospital. | **Length of stay:** Number of infants (%) with an index hospital length of stay <3 days versus ≥3 days.  Normal (n=713) vs. high (n=160):  LOS <3 days:  430/713 (60%) vs. 103/160 (64%)  LOS ≥3 days:  283/713 (40%) vs. 57/160 (36%).  **Primary outcomes:**   - Use of intensive care therapy (ICU admission, intubation, and/or receipt of continuous positive pressure ventilation);   Plus other secondary outcomes reported in the article. | Note, adjusted odds ratios not reported for LOS. |
| **Rodriguez-Gonzalez 2023**  Correlation between urinary and serum NT‐proBNP in acute bronchiolitis: A pilot study. Pediatric Pulmonology. 2023 Feb;58(2):492-9.    **Country**  Spain  **Study Type**  Observational (pilot study)  **Location of study**  Puerta del Mar University Hospital, Cadiz, Spain  **Study Aim**  To analyse the association of urinary and serum NT-proBNP and disease severity in acute bronchiolitis. | **Sample**  N=17 infants hospitalised for bronchiolitis.  **Characteristics**  Median (IQR) age in days: 68 (36 to 91).  Ethnicity not reported.  **Enrolment setting**  ED, Paediatric ward  **Key inclusion criteria**  Age criteria: <12 months  Infants admitted to hospital with bronchiolitis of any severity.  Exclusion criteria were presence of significant congenital anomalies including cardiac diseases, chronic renal diseases, acute kidney injury, incomplete data, refusal of parental consent. | **Investigation**  Urinary and serum N-terminal pro-brain natriuretic peptide (NT-proBNP) concentrations, time-matched and collected at time of inclusion (with possibility of later samples during hospitalisation).  **Comparator**  No allocated comparator (testing was performed on all patients, and comparisons were made by test results). | Pilot observational study, prospective.  Z-log values were calculated to adjust for age in days. | **Length of stay:** Median (IQR) length of hospital stay in days.  Total sample (n=17):  4 (2 to 11)  Urinary log-10-NT-proBNP/creatinine ratio:  Spearman’s ρ=0.84, *p*<.001.  **Death:** Number of infants (%).  0/17 (0%).  **Primary outcomes:**   - Relationship between serum and urine NT-proBNP levels;   Plus other secondary outcomes reported in the article. |  |
| **Rodriguez-Gonzalez 2022**  Routine laboratory test to assess the need of respiratory support in acute bronchiolitis. Pediatric Pulmonology. 2022 May;57(5):1339-47.    **Country**  Spain  **Study Type**  Observational (prospective)  **Location of study**  Puerta del Mar University Hospital, Cadiz, Spain  **Study Aim**  To explore the relationship between several biomarkers available at the paediatric emergency care setting with the need for advanced respiratory support in infants with bronchiolitis. | **Sample**  N=149 infants hospitalised for bronchiolitis.  N=37 PICU patients.  **Characteristics**  Median (IQR) age in months: 1 (0.5 to 2.5).  Ethnicity not reported.  **Enrolment setting**  Paediatric ED, PICU  **Key inclusion criteria**  Age criteria: <12 months  Infants admitted to hospital with bronchiolitis from 1 October 2018 to 1 May 2020.  Exclusion criteria were history of malnutrition, dehydration, haemodynamically significant heart disease, chronic respiratory disease, renal insufficiency, hepatic impairment, immunosuppression, tumour, metabolic disease, or chronic inflammatory disease. Only pre-term infants with moderate to severe bronchopulmonary dysplasia requiring medication or supplemental oxygen therapy at home were excluded. Receipt of any medication other than nebulised hypertonic saline or any respiratory support other than low flow or high flow oxygen before the clinical assessment. | **Investigation**  Blood samples performed at time of hospital admission, including glycemia (mg/dl), CRP (mg/L), CRP/Albumin, NT-ProBNP (pg/ml).  **Comparator**  No allocated comparator (testing was performed on all patients, and comparisons were made by test results). | Single centre, prospective observational study.  Multivariate logistic regression performed to control for age, bacterial superinfection, comorbidity known as risk factors for severe bronchiolitis; only reported for primary outcome. | **Length of stay:** Median (IQR) length of hospitalisation in days. Spearman’s correlation coefficient (ρ).  Overall sample:  4 (2 to 9)  Glycemia:  ρ=0.252, *p*=.005  CRP:  ρ=0.348, *p*=.000  CRP/Albumin:  ρ=0.340, *p*=.000  NT-ProBNP:  ρ=0.581, *p*=.000  **Death:** Number of infants (%).  0/149 (0%).  **ICU length of stay:** Median (IQR) length of ICU stay in days. Spearman’s correlation coefficient (ρ).  Glycemia:  ρ=0.256, *p*=.15  CRP:  ρ=0.043, *p*=.81  CRP/Albumin:  ρ=-0.07, *p*=.99  NT-ProBNP:  ρ=0.490, *p*=.02  **Primary outcomes:**   - Need for advanced respiratory support;   Plus other secondary outcomes reported in the article. |  |
| **Sun 2020**  The implications of platelet count changes during hospitalization in the disease management of paediatric patients with bronchiolitis. Infectious Diseases. 2020 Nov 1;52(11):786-92.  **Country**  China  **Study type**  Retrospective observational  **Location of study**  Children’s Hospital of Soochow University, Suzhou, Jiangsu Province, China  **Study aim**  To assess the significance of platelet count on admission and platelet count changes. | **Sample**  N=404 infants with acute bronchiolitis  n=79 with thrombocytosis  n=325 with normal platelet count  n=38 admitted to PICU (9.4% of sample)  **Characteristics**  Median age in months (overall sample): 8 months (IQR not reported).  Average age of PICU sample not reported.  Ethnicity not reported.  **Enrolment setting**  Hospital ward, PICU.  **Key inclusion criteria**  Age ≤24 months.  Infants admitted to hospital and diagnosed with bronchiolitis between January 2015 to December 2018. Bronchiolitis defined as first episode of wheezing, cough or rhinorrhoea; wheezes or crackles, or over-aeration or peribronchial infiltration with or without atelectasis; complete blood count results obtained within 6 hours after admission and 3 to 5 days after admission.  Infants were excluded with immunosuppression, primary haematological disorders, active tuberculosis, trauma or surgery in the preceding 2 weeks, chronic gastrointestinal disorder, prematurity, CHD, bronchopulmonary malformation, genetic or neurological disorder. | **Test**  Platelet count on admission. Thrombocytosis was defined as a platelet count >500x10^9^/L.  Complete blood counts were obtained within 6 hours and 3 to 5 days of hospital admission.  **Comparator**  No allocated comparator (all participants received the same testing, and were compared by the results). | Retrospective observational study | **Length of stay:** Median length of stay (IQR) in days.  Thrombocytosis (n=79) vs. normal platelet count (n=325):  7.0 (6.0 to 8.0) vs. 7.0 (7.0 to 8.0), *p*=.54.  **Length of ICU stay:** Median length of PICU day in days (IQR).  Thrombocytosis (n=5) vs. normal platelet count (n=33):  6.0 days (4.5 to 10.5) vs. 5.50 days (4.0 to 9.0), *p*=.55.  **Primary outcomes:**   - Severe disease (defined as length of hospitalisation >24 hours); - Clinical characteristics; - Disease severity;   Plus secondary outcomes reported in the article. |  |

### Virological tests

| **Study design** | **Participants** | **Investigation** | **Methods** | **Outcomes and results** | **Comments** |
| --- | --- | --- | --- | --- | --- |
| **Akande 2024**  Impact of respiratory bacterial codetection on outcomes in ventilated infants with bronchiolitis. The Pediatric Infectious Disease Journal. 2024 Feb 1;43(2):117-22.  **Country**  United States of America  **Study type**  Retrospective observational  **Location of study**  Nationwide Children’s Hospital, Columbus, Ohio, USA  **Study aim**  To examine the association between codetection and outcomes in infants requiring emergency department (ED) intubation for bronchiolitis. | **Sample**  N=149 infants with bronchiolitis admitted to PICU (148 analysed)  **Characteristics**  Median (IQR) age in months: 1.3 (0.75-1.86)  Ethnicity:  White 58%  Black 23%  Other 11%  Hispanic 8%  **Enrolment setting**  ED (with subsequent PICU admission)  **Key inclusion criteria**  Aged <2 years.  Infants were included with viral bronchiolitis requiring endotracheal intubation in the ED between July 2012 – June 2017, and subsequently admitted to PICU.  Exclusion criteria were tracheostomy, admission to a unit other than PICU. | **Test**  RSV positive status at hospital admission. Viral testing results were categorised into:  a. RSV positive;  b. RSV positive plus other;  c. Only non-RSV;  d. Polyviral.  **Comparator**  No comparator. | Single-centre, retrospective cohort study.  Univariate and multivariate regression, adjusted for significant variables in univariate analyses (presence of prematurity, codetection, any other positive culture). | **ICU length of stay:** Adjusted and unadjusted relative risk (Adj RR; RR) with 95% confidence intervals (CI) for longer marginal mean ICU length of stay in days, associated with a positive RSV status^1^.  RSV positive only (n=83) vs. other viral categories (n=85)^2^:  RR 1.35 (95% CI 1.15-1.60), *p*<.001.  Adj RR 1.40 (95% CI 1.20-1.63), *p*<.0001.  **Primary outcomes:**   - Mechanical ventilation; - ICU length of stay;   Plus secondary outcomes reported in the article. | ^1^Average ICU LOS values not reported by virological results. The sample median ICU LOS was 6.8 days (IQR 5-10).  ^2^Comparator assumed; not explicitly reported in paper. |
| **Ambrożej 2024**  Association of respiratory virus types with clinical features in bronchiolitis: Implications for virus testing strategies. A systematic review and meta-analysis. Paediatric Respiratory Reviews. 2024 Sep 17.  **Country**  Multiple  **Study Type**  Systematic review with meta-analysis  **Location of study**  Extracted studies: Brazil (2), Chile (2), France (1), Qatar (1), Turkey (1), USA (3)  **Study Aim**  To evaluate whether clinical features and patient history can differentiate the viral aetiology of bronchiolitis. | **Sample**  N=6,305 infants overall  n=4,396 infants with RSV or RV bronchiolitis  n=3,101 infants in studies reporting our review outcomes  **Characteristics**  Mean age not reported.  The upper age limits were 24 months (n=10 studies), 12 months (n=5 studies), and 6 months (n=4 studies).  9 of 10 studies reporting on our guideline outcomes met our age eligibility criteria <12m^1^.  Ethnicity not reported.  **Enrolment setting**  ED, hospital ward, outpatient clinic, ICU  **Key inclusion criteria**  Age <24 months.  Studies were included that reported on infants with a clinical presentation described as either bronchiolitis, an acute wheezing episode, or an ALRI, with assessment at a hospital (ED and/or ward), or as outpatients assessed by the study investigators. Studies were required to assess at least two groups of patients with virologically confirmed RSV or RV infection.  Studies were excluded in infants presenting with confirmed respiratory infection with only one viral agent. | **Investigation**  RSV-positive bronchiolitis, confirmed in virological testing.  **Comparator**  Rhinovirus (RV)-positive bronchiolitis, confirmed in virological testing.  The article does not report on the type of virological testing that was performed within the included studies to detect RSV infection. | Systematic review with meta-analysis of 18 studies and narrative synthesis of 21 studies.  Of these studies, 17 were prospective observational, 3 were retrospective observational, and 1 was a RCT.  10 studies reported data on our guideline outcomes and were extracted.  Databases: MEDLINE, PubMed, Web of Science, Cochrane Library.  Date of last search: 29^th^ March 2022. | **Rate of hospitalisation:** Rate of hospital admission (not quantitatively synthesized).  RSV-bronchiolitis vs. RV-bronchiolitis:  1) Nascimento 2010:  2 of 13 (15%) vs. 19 of 36 (53%), *p* not reported.  2) Luchsinger 2014:  10 of 22 (46%) vs. 57 of 74 (77%), ***p*<.05.**  3) Diaz 2015:  1 of 27 (4%) vs. 49 of 60 (82%), *p* not reported.  **Rate of PICU admission:** Odds ratio (OR) (95% CI) for PICU transfer.  RSV-bronchiolitis vs. RV-bronchiolitis (5 studies, N=1,010; I^2^=42%)^1^:  130 of 818 (15.9%) vs. 28 of 192 (14.6%)  OR 0.87 (95% CI 0.01 to 3.97), *p*=.76.  **Length of stay:** Length of hospital stay (varying measures, not quantitatively synthesized).  RSV-bronchiolitis vs. RV-bronchiolitis:  1) Marguet 2009:  Median 3 days (IQR 3 to 4.7) vs. 6 days (IQR 5 to 8), ***p*<.05**.  2) Mansbach 2016:  Mean 2.6 days (SD 3.5) vs. 3.3 days (3.4), *p* not reported.  Median 2 days (IQR 1 to 3) vs. 2 days (IQR 1 to 4), *p* not reported.  3) Janahi 2017 (n of infants with hospitalisation length >4 days):  52 of 58 (90%) vs. 133 of 160 (83%), *p* not reported.  4) Hasegawa 2019:  Mean 2.9 days (SD 5.8) vs. 3.0 days (SD 3.7), p not reported.  Median 2 days (IQR 1 to 2) vs. 2 days (IQR 1 to 3), *p* not reported.  5) Arroyo 2020^1^:  Mean 6.47 days (SD 11.01) vs. 12.91 days (SD 20.41), *p* not reported.  **Primary outcomes:**   - Presence of wheezing on auscultation; - Fever; - Severity of infection; - Personal history of atopy in the child; - Parental history of atopy or asthma;   Plus other secondary outcomes reported in the article. | The 10 eligible studies were rated as moderate to low risk of bias, based on a modified Newcastle Ottawa quality assessment scale for cross-sectional studies.  ^1^ Arroyo 2020, included in the meta-analysis, had median ages ranging from 6.36 months (IQR 8.64) to 12.78 months (IQR 12.24), that varied by bronchiolitis phenotype (mild, hypoxaemia, wheezing) and control groups. |
| **Bamberger 2012**  What is the clinical relevance of respiratory syncytial virus bronchiolitis?: findings from a multi-center, prospective study. European journal of clinical microbiology & infectious diseases. 2012 Dec;31:3323-30.  **Country**  Israel  **Study Type**  Observational (prospective)  **Location of study**  3 sites (HaEmek Medical Center, Bnai Zion Medical Center, Meyer Children’s Hospital) in Israel.  **Study Aim**  To evaluate whether the etiological agents of acute bronchiolitis affect its course. | **Sample**  N=366 infants hospitalised with bronchiolitis (n=311 analysed for length of ICU stay outcome)  n=149 infants with only RSV infection  n=92 infants with RSV plus infection with another agent  n=44 infants with no RSV (but infection with another agent)  n=26 infants with no pathogen detected  **Characteristics**  Mean age: 17.5 weeks (SD 16.6)  Ethnicity not reported.  **Enrolment setting**  Hospital ward, PICU  **Key inclusion criteria**  Age criteria: <24 months  Previously healthy children who presented with 4 or less days duration of acute bronchiolitis. Bronchiolitis was diagnosed as upper respiratory tract infections with cough and at least two of the following signs: chest retractions, tachypnoea, wheezing, or rales on auscultation.  Children were excluded who were hospitalised within 2 weeks before the index admission, developed nosocomial bronchiolitis, or received passive RSV vaccination in the current season. | **Investigation**  Viral detection from PCR assay with nasopharyngeal aspirate. Tests were performed for RSV, rhinovirus, bocavirus, Bordetella pertussis, hMPV, influenza A, adenovirus, influenza B, and parainfluenza 3.  Testing enabled dividing the patients into the following groups for analysis:  a. Only RSV infection  b. Also RSV (RSV plus at least one other agent)  c. No RSV (RSV negative but infection with another agent)  d. No pathogen identified  **Comparator**  No allocated comparator (testing was performed on all included patients, and comparisons were made by test results). | Prospective observational study, multicentre (3 sites) | **Length of stay:** Number of infants with the specified mean length of stay in days (≤3 days, 4-7 days, >7days).  ≤3 days:  Only RSV: 62 (41.6%)  Also RSV: 47 (52.2%)  No RSV: 28 (65.1%)  No pathogen: 11 (44%)  4-7 days:  Only RSV: 76 (51%)  Also RSV: 35 (38.9%)  No RSV: 13 (38.9%)  No pathogen: 11 (44%)  >7 days:  Only RSV: 11 (7.4%)  Also RSV: 8 (8.9%)  No RSV: 2 (4.7%)  No pathogen: 3 (12%)  *p*=.01.  (Post hoc tests, only RSV vs. no RSV *p*<.02).  **Length of ICU stay:** Number of infants with the specified mean length of stay (0 days, 1 day, >1 day) in the PICU in days.  0 days in PICU:  Only RSV: 144/149 (96.6%)  Also RSV: 88/92 (95.7%)  No RSV: 41/44 (93.2%)  No pathogen: 23/26 (88.5%)  1 day in PICU:  Only RSV: 0/149 (0%)  Also RSV: 1/92 (1.1%)  No RSV: 1/44 (2.3%)  No pathogen: 1/26 (3.9%)  >1 day in PICU:  Only RSV: 5/149 (3.4%)  Also RSV: 3/92 (3.3%)  No RSV: 2/44 (4.6%)  No pathogen: 2/26 (7.7%)  *p*=.437.  **Primary outcomes:**   - Clinical severity score at admission - Overall disease severity   And other secondary outcomes reported in the article. |  |
| **Bermúdez-Barrezueta 2023**  Outcomes of viral coinfections in infants hospitalized for acute bronchiolitis. Virology Journal. 2023 Oct 16;20(1):235.  **Country**  Spain  **Study Type**  Retrospective cohort  **Location of study**  Hospital Clinico Universitario de Valladolid, Valladolid, Spain.  **Study Aim**  To evaluate the effect of viral co-infection on the progression and severity of acute bronchiolitis, as evidenced through hospitalisation days and PICU admissions. | **Sample**  N=445 infants admitted to hospital with bronchiolitis  n=270 with single virus infection  n=175 with viral co-infection (n=126 with 2 viruses; n=49 3+ viruses)  **Characteristics**  Median age (months): 2.5 months (IQR 1.4 to 4.6)  Ethnicity not reported.  **Enrolment setting**  Hospital ward, PICU  **Key inclusion criteria**  Age <24 months.  Infants were included who were admitted to hospital for acute bronchiolitis during 1 September 2012 and 15 March 2020, for whom at least one virus was identified by molecular diagnostic techniques in nasopharyngeal lavage or pharyngeal smear samples that were collected during the first 24 hours of hospital admission.  Bronchiolitis was defined as the first episode of respiratory distress with wheezing and/or crackles or rales, preceded by a catarrhal stage, in an infant aged <two years.  Infants were excluded who did not undergo molecular testing. | **Investigation**  Single virus infection or co-infection with multiple viruses, detected via molecular testing of nasopharyngeal lavage or pharyngeal smear samples collected during the first 24 hours of hospital admission.  Viral co-infection was defined as the simultaneous identification of two or more viruses in a single respiratory sample.  The molecular techniques used for virological testing were the Luminex NxTAG Respiratory Pathogen PCR panel, and the FilmArray Respiratory Panel.  **Comparator**  No allocated comparator (testing was performed on all included patients, and comparisons were made by test results). | Retrospective cohort study using medical records and data from the Clinical Documentation and Archives Unit.  Univariate and multivariate Cox regression analyses assessed factors predictive of longer length of stay and PICU admission. | **ICU admission:** Number of infants (%) admitted to ICU.  Single virus (n=270) vs. co-infections (n=175):  61 of 270 (22.6%) vs. 44 of 175 (25.1), *p*=.54.  No virus (reference) vs. 2 viruses:  Adj HR 0.94 (95% CI 0.53 to 1.67), *p*=.56.  No virus (reference) vs. ≥3 viruses:  Adj HR 1.84 (95% CI 0.88 to 3.86), *p*=.07.  **Length of stay:** Median length of hospital stay in days (IQR).  Single virus (n=270) vs. co-infections (n=175):  6 (4 to 8) vs. 7 (5 to 10), *p*=.004.  No virus (reference) vs. 2 viruses:  Adj HR 0.89 (95% CI 0.69 to 1.07), *p*=.18.  No virus (reference) vs. ≥3 viruses:  Adj HR 0.57 (95% CI 0.41 to 0.79), *p*<.001.  **Length of ICU stay:** Median length of PICU stay in days (IQR).  Single virus (n=270) vs. co-infections (n=175):  Median 4 days (IQR 3 to 6) vs. 5 days (IQR 3 to 7), *p*=.08.  **Primary outcomes:**   - Length of hospitalisation; - Rate of PICU admission;   Plus other secondary outcomes reported in the article. |  |
| **Boggio 2023**  Clinical characterization of human bocavirus 1 infection in infants hospitalized in an intensive care unit for severe acute respiratory tract disease. Diagnostic Microbiology and Infectious Disease. 2023 Nov 1;107(3):116050.  **Country**  Argentina  **Study Type**  Prospective cross-sectional  **Location of study**  Cordoba, Argentina (hospital not reported)  **Study Aim**  To describe the clinical characteristics and evolution of infants who were hospitalised for severe acute respiratory infection (SARI) due to human bocavirus (HBoV1), and compare them to infants with RSV infection, taking into account patients with single detection and no previous comorbidities. | **Sample**  N=141 infants admitted to the ICU with an acute respiratory infection.  n=65 infants with single RSV infection.  n=5 infants with single HBoV1 infection.  **Characteristics**  Mean age in months (SD):  Overall sample: 5.4 months (SD 4.5)  Ethnicity not reported.  **Enrolment setting**  ICU  **Key inclusion criteria**  Age <24 months.  Infants were included who were admitted to the ICU with a diagnosis of acute respiratory infection (ARI) during April 2021 to 2022. ARI could include bronchiolitis, recurrent obstructive bronchitis, bacterial pneumonia, or pertussis like syndrome.  Infants were excluded for whom collecting a clinical specimen for diagnosis was difficult or impossible. | **Investigation**  Viral infections were detected by use of the direct immunofluorescence (IF) technique with nasopharyngeal aspirates, collected within the first 24 hours of hospital admission.  Testing was used to detect RSV infection and human bocavirus (HBoV1) infection.  **Comparator**  No allocated comparator (testing was performed on all included patients, and comparisons were made by test results). | Cross-sectional study with prospectively enrolled patients. | **Length of ICU stay:** Mean days of stay in the ICU (SD).  RSV vs. HBoV1:  13.11 days (SD 8.19) vs. 14.6 days (SD 7.92), *p*>.05.  **Primary outcomes:**   - Clinical characteristics;   Plus other secondary outcomes reported in the article. |  |
| **Celik 2020**  Neutrophil responses in infants with acute bronchiolitis. Ann Clin Anal Med. 2020;11(04):277-82.  **Country**  Turkey  **Study Type**  Observational (prospective)  **Location of study**  Hospital not reported; Aydin, Turkey  **Study Aim**  To evaluate neutrophil counts and to explore its clinical significance in infants with acute bronchiolitis. | **Sample**  N=146 infants  n=94 hospitalised with acute bronchiolitis (n=47 RSV, n=47 rhinovirus (RV))  n=52 healthy infant controls who presented to a child health outpatient clinic  **Characteristics**  Median age in days (IQR): 82 (52.2-180).  Ethnicity not reported.  **Enrolment setting**  Paediatric ward, outpatient clinic  **Key inclusion criteria**  Age criteria: <12 months  Infants admitted to hospital with acute bronchiolitis, or healthy infants who presented to a child health outpatient clinic.  Exclusion criteria were hospitalisation in prior 2 weeks before current admission, prematurity, bronchopulmonary dysplasia, congenital heart disease, immunodeficiency, chronic lung disease. | **Investigation**  Nasopharyngeal swab samples to assess for RSV and RV infection.  **Comparator**  No allocated comparator (testing was performed on all patients, and comparisons were made by test results). | Observational cohort study, prospective | **Length of stay:** Mean (SD) duration of hospital stay in days.  RSV vs. RV:  6.39 (3.70) vs. 5.82 (3.48), *p*=.453.  **Primary outcomes:**   - Clinical significance of complete blood count parameters;   Plus other secondary outcomes reported in the article. |  |
| **Coleman 2019**  Respiratory support during bronchiolitis due to one virus versus more than one virus: an observational study. Journal of Pediatric Intensive Care. 2019 Dec;8(04):204-9.  **Country**  Australia  **Study Type**  Observational (retrospective)  **Location of study**  The John Hunter Children’s Hospital, Newcastle, NSW, Australia  **Study Aim**  To determine if there is a difference in the duration of respiratory support which bronchiolitis is caused by one versus more than one virus. | **Sample**  N=306 infants admitted to PICU with bronchiolitis  n=215 were infected with a single virus  n=91 were infected with more than one virus  **Characteristics**  Median age (days):  One virus: 102 (IQR 44 to 226)  Two + viruses: 111 (IQR 54 to 187)  Ethnicity not reported.  **Enrolment setting**  PICU  **Key inclusion criteria**  Age criteria: <12 months  Infants admitted to PICU with a confirmed diagnosis of bronchiolitis with the International Classification of Diseases 10^th^ edition diagnostic criteria for bronchiolitis, and microbiological evidence of viral respiratory tract infection on nasopharyngeal aspirate from a PCR test.  Patients were excluded who did not have a confirmed viral infection through PCR, or if PICU or medical data were unavailable (n=30). | **Investigation**  Viral respiratory tract infection indicated by PCR assay with nasopharyngeal aspirate.  **Comparator**  No allocated comparator (testing was performed on all included patients, and comparisons were made by test results). | Retrospective observational study, single-centre | **Length of stay:** Median (IQR) hospital length of stay in days.  1 virus (n=215) vs. 2+ viruses (n=91):  4 (3 to 6) vs. 5 (4 to 9), *p*=.03.  **Length of ICU stay:** Median (IQR) length of PICU stay in days collected from medical records.  1 virus (n=215) vs. 2+ viruses (n=91):  2.1 days (IQR 1 to 3) vs. 3.0 days (IQR 1 to 4), *p*=.06.  **Primary outcomes:**   - Duration of respiratory support   Plus other secondary outcomes reported in the article. |  |
| **Erdede 2023**  Neutrophil-to-lymphocyte ratio and the systemic Immune-inflammation index: biomarkers in infants with bronchiolitis: a cross-sectional study. Japanese Journal of Infectious Diseases. 2023 Nov 30;76(6):351-7.    **Country**  Turkey  **Study Type**  Observational (retrospective)  **Location of study**  Zeynep Kamil Maternity and Children’s Disease Training and Research Hospital, Istanbul, Turkey  **Study Aim**  To evaluate the use of systemic immune-inflammation index and neutrophil-to-lymphocyte ratios in infants with bronchiolitis, and to determine whether they could predict bronchiolitis with RSV alone or co-infection with other viruses, and disease severity. | **Sample**  N=155 infants hospitalised for bronchiolitis  **Characteristics**  Mean (SD) age in months: 5.9 (5.5).  Ethnicity not reported.  **Enrolment setting**  Paediatric ward  **Key inclusion criteria**  Age criteria: <24 months  Infants admitted to hospital with moderate or severe bronchiolitis caused by RSV and/or other viral pathogens between 1 October 2021 to 31 January 2022, with first episode of wheezing. Illness severity determined by scoring system (Gadjos).  Exclusion criteria were presence of comorbidity, such as primary immunodeficiency, neuromuscular disease, chronic lung and heart disease. Presence of pneumonia confirmed by chest radiography, or SARS-CoV-2 infection. PICU admission or need for mechanical ventilation. | **Investigation**  Viral testing with nasopharyngeal aspirate samples and multiplex real-time PCR performed on day one of hospital admission, to assess for 15 viral pathogens. Results categorised into:  a) RSV infection only  b) Non-RSV infection only  c) RSV co-infection  **Comparator**  No allocated comparator (testing was performed on all patients, and comparisons were made by test results). | Single centre retrospective observational study using electronic health records. | **Length of stay:** Mean (SD) length of stay in days.  RSV alone (n=58) vs. non-RSV alone (n=45):  6.16 (2.31) vs. 5.49 (2.16), *p*=.14.  RSV alone (n=58) vs. RSV co-infection (n=52):  6.16 (2.31) vs. 6.85 (2.37), *p*=.13.  **Primary outcomes:**   - Disease severity score;   Plus other secondary outcomes reported in the article. | Only LOS results for SII were reported. Mean LOS not reported. |
| **Huguenin 2012**  Broad respiratory virus detection in infants hospitalized for bronchiolitis by use of a multiplex RT‐PCR DNA microarray system. Journal of medical virology. 2012 Jun;84(6):979-85.    **Country**  France  **Study Type**  Observational (prospective)  **Location of study**  Reims University Medical Centre, Reims, France  **Study Aim**  To evaluate the analytical and clinical performance of a commercially available multiplex RT-PCR DNA microarray. | **Sample**  N=138 infants hospitalised for bronchiolitis  **Characteristics**  Mean age (SD) in months: 4 (1.4)  Ethnicity not reported.  **Enrolment setting**  Paediatric ward  **Key inclusion criteria**  Age criteria: ≤12 months  Infants admitted to hospital with bronchiolitis from October 2007 to September 2008, within 3 days of symptom onset.  Exclusion criteria were not reported. | **Investigation**  Nasopharyngeal aspirate samples taken at admission, processed with multiplex RT-PCR DNA microarray and conventional respiratory virus detection assays (for >17 respiratory viruses). Classified into multiple versus single virus infection.  **Comparator**  No allocated comparator (testing was performed on all patients, and comparisons were made by test results). | Single centre prospective observational study | **ICU admission:** Number (%) of infants admitted to ICU.  Single vs. multiple viral infections:  5/41 (12.2%) vs. 5/85 (5.9%), *p*=.29.  **Length of stay:** Mean (SD) length of hospital stay in days.  Single (n=41) vs. multiple viral infections (n=85):  6 (4.5) vs. 5 (3.3), *p*=.52.  Reports a significantly higher length of stay (1.7 (SD 0.6) days, *p*=.04) for infants infected with RSV-A/B in age-adjusted multivariable analyses; RSV negative comparison group assumed.  **Primary outcomes:**   - NR;   Plus other secondary outcomes reported in the article. |  |
| **Mansbach 2012**  Prospective multicenter study of viral etiology and hospital length of stay in children with severe bronchiolitis. Archives of pediatrics & adolescent medicine. 2012 Aug 1;166(8):700-6.    **Country**  United States of America  **Study Type**  Observational (prospective)  **Location of study**  16 hospitals, USA  **Study Aim**  To determine whether hospital length of stay for acute bronchiolitis is influenced by the infecting pathogen. | **Sample**  N=2,207 infants hospitalised for bronchiolitis.  n=1,866 infants with RSV and/or HRV analysed  **Characteristics**  Median (IQR) age in months: 4 (2 to 8).  Ethnicity:  White 62.0%  Hispanic 35.6%  **Enrolment setting**  Paediatric ward, ICU  **Key inclusion criteria**  Age criteria: <24 months  Infants admitted to hospital with bronchiolitis during winter seasons of 2007 to 2010.  Exclusion criteria were previous enrolment.  Enrolments each month paused after meeting a monthly quota. | **Investigation**  Nasopharyngeal aspirate, collected within 24 hours of admission to hospital or ICU, analysed for multiple viruses using PCR assay.  **Comparator**  No allocated comparator (testing was performed on all patients, and comparisons were made by test results). | Multicentre, prospective observational cohort study (MARC-15).  Multivariable model adjusted for 15 demographic and clinical characteristics, and by site. Included age, comorbodiity, and illness-severity related outcomes. | **ICU admission:** Proportion of infants (%) admitted to ICU.  RSV only (n=1,075) vs. HRV only (n=167) vs. RSV plus HRV (n=287), vs. RSV plus non-HRV (n=227) vs. HRV plus non-RSV (n=110):  17.2% vs. 15.8% vs. 18.1% vs. 17.1% vs. 17.6%, *p*=.98.  **Length of stay:** Adjusted odds ratio (Adj OR) (95% CI) for a hospital length of stay of ≥3 days.  RSV only (n=1,075) vs. HRV only (n=167):  1 (reference) vs. 0.36 (0.20 to 0.63), *p*<.001.  RSV only (n=1,075) vs. RSV plus HRV (n=287):  1.33 (1.02 to 1.73), *p*=.04.  RSV only (n=1,075) vs. RSV plus any other non-HRV pathogen (n=227):  1.06 (0.67 to 1.69), *p*=.79.  RSV only (n=1,075) vs. HRV plus any other non-RSV pathogen (n=110):  0.39 (0.23 to 0.66), *p*<.001.  **Primary outcomes:**   - Hospital length of stay;   Plus other secondary outcomes reported in the article. |  |
| **Petrarca 2018**  Acute bronchiolitis: Influence of viral co‐infection in infants hospitalized over 12 consecutive epidemic seasons. Journal of Medical Virology. 2018 Apr;90(4):631-8.  **Country**  Italy  **Study Type**  Observational (prospective)  **Location of study**  Sapienza University Hospital, Rome, Italy  **Study Aim**  To evaluate clinical and serological differences in infants with bronchiolitis from a single or multiple viruses. | **Sample**  N=486 infants hospitalised for bronchiolitis with at least one virus detected  n=431 were infected with a single virus  n=55 were infected with more than one virus  **Characteristics**  Median age in months (IQR): 2.03 (0.23 to 11.17)  Ethnicity not reported.  **Enrolment setting**  Paediatric ED  **Key inclusion criteria**  Age criteria: <12 months  Included were full-term infants admitted to hospital for bronchiolitis, with at least one virus detected in nasopharyngeal aspirate.  Exclusion criteria were prematurity, underlying chronic diseases (cystic fibrosis, interstitial lung disease, congenital heart disease, immunodeficiency). | **Investigation**  Viral detection from reverse transcription polymerase chain reactions (RT-PCR) of nasopharyngeal aspirate for 14 respiratory viruses: RSV, influenza, human coronavirus, 229E, NL-63, HKU1, adenovirus, hRV, parainfluenza virus, human metapneumovirus (hMPV), human bocavirus (hBoV).  **Comparator**  No allocated comparator | Observational study, prospective with parent survey and medical record review. | **Rate of ICU admission:** number of infants (%) admitted to ICU.  Single infection vs. co-infection:  48 of 431 (11.2%) vs. 13 of 55 (23.6%), *p*=NS.  RSV vs. RSV co-infection:  38 of 320 (11.9%) vs. 6 of 45 (13.3%), *p*=NS.  hRV vs. hRV co-infection:  8 of 60 (13.6%) vs. 2 of 29 (6.9%), *p*=NS.  RSV vs. hRV vs. hRV + RSV:  38 of 320 (11.9%) vs. 8 of 60 (13.6%) vs. 2 of 20 (9.5%), *p*=NS.  **Length of stay:** median (range) days of hospitalisation.  Single infection (n=431) vs. co-infection (n=55):  5 (1-45) vs. 4 (2-12), *p*=NS.  RSV (n=320) vs. RSV co-infection (n=45):  5 (1-27) vs. 5 (2-12), *p*=NS.  hRV (n=60) vs. hRV co-infection (n=29):  5 (2-16) vs. 5 (2-9), *p*=NS.  RSV (n=320) vs. hRV (n=60) vs. hRV + RSV (n=20):  5 (1-27) vs. 5 (2-16) vs. 5 (3-9), *p*=NS.  **Primary outcomes:**   - Not specified “clinical or serological differences”;   Plus other secondary outcomes reported in the article. |  |
| **Sai Kotha 2023**  A Comparative Study On Clinical Profile Of Rsv And Non-Rsv Bronchiolitis Children Attending A Tertiary Care Centre. Journal of Pharmaceutical Negative Results. 2023 Apr 1;14(2).    **Country**  India  **Study Type**  Observational (prospective)  **Location of study**  Chettinad Hospital and Research Institute, Chennai, India  **Study Aim**  To compare the demographics, clinical characteristics, and prognosis of children with RSV bronchiolitis and non-RSV bronchiolitis admitted to hospital. | **Sample**  N=90 infants hospitalised with a bronchiolitis-like illness.  **Characteristics**  83.3% aged ≤12 months.  Ethnicity not reported.  **Enrolment setting**  Paediatric ward  **Key inclusion criteria**  Age criteria: 1 month to 3 years  Infants admitted to hospital with a diagnosis like bronchiolitis between July and December 2021.  Exclusion criteria were family history of asthma and chronic lung disease, or critical congenital heart defect. | **Investigation**  Nasopharyngeal aspirate taken within 24 hours of hospital admission, with viral panel. Patients classified into RSV and non-RSV groups.  **Comparator**  No allocated comparator (testing was performed on all patients, and comparisons were made by test results). | Prospective observational study | **Length of stay:** Mean (SD) duration of hospital stay in days.  RSV (n=45) vs. non-RSV (n=45):  4.44 (1.83) vs. 4.27 (1.74), *p*=.64.  **Primary outcomes:**   - NR;   Plus other secondary outcomes reported in the article. | Not specified what the non-RSV group consisted of |
| **Tan 2021**  Etiology, clinical characteristics and coinfection status of bronchiolitis in Suzhou. BMC Infectious Diseases. 2021 Feb 1;21(1):135.    **Country**  China  **Study Type**  Observational (retrospective)  **Location of study**  Children’s Hospital of Soochow University, Suzhou, China  **Study Aim**  To investigate the prevalence of pathogens in hospitalised children with bronchiolitis, and to evaluate the clinical characteristics of bronchiolitis with and without co-infections. | **Sample**  N=1,012 infants hospitalised for bronchiolitis (n=842 analysed with at least one pathogen)  **Characteristics**  Median (IQR) age in months: 5 (NR)  Ethnicity not reported.  **Enrolment setting**  Paediatric ward  **Key inclusion criteria**  Age criteria: 1 month to 2 years  Infants admitted to hospital with bronchiolitis between November 2011 and December 2018, experiencing their first episode of wheezing.  Exclusion criteria were immunodeficiency, history of a diagnosis of chronic lung disease or congenital heart disease. | **Investigation**  Nasopharyngeal aspirates for viral diagnostic tests. Participants divided into:  a) single virus  b) mixed viruses  **Comparator**  No allocated comparator (testing was performed on all patients, and comparisons were made by test results). | Retrospective observational study of medical records | **ICU admission:** proportion (%) of infants admitted to ICU.  Single virus (n=614) vs. mixed virus (n=70):  10.6% vs. 10%  **Length of stay:** Median length of stay in days (IQR not reported).  Single virus (n=614) vs. mixed virus (n=70):  8 vs. 8  **Primary outcomes:**   - NR;   Plus other secondary outcomes reported in the article. | Multiple virus plus mycoplasma pneumonae (MP) and MP-only infection were also reported; however this data were considered a different subgroup (broncho-pneumonia) |
| **Yu 2010**  The severity of bronchiolitis is not dependent on the co-infection of RSV with other respiratory viruses. Journal of Pediatric Infectious Diseases. 2010 Sep;5(03):255-61.    **Country**  China  **Study Type**  Observational (prospective)  **Location of study**  Children’s Hospital, Chongqing Medical University, Chongqing, China  **Study Aim**  To clarify the impact of RSV co-infection with other viruses on the severity of bronchiolitis and airway inflammation. | **Sample**  N=112 infants hospitalised for bronchiolitis.  **Characteristics**  Median (IQR) age in months: 5.9 (4.1)  Ethnicity not reported.  **Enrolment setting**  Paediatric ward  **Key inclusion criteria**  Age criteria: <3 years  Infants admitted to hospital with bronchiolitis during the 2006 to 2008 winter seasons.  Exclusion criteria were not reported. | **Investigation**  Nasopharyngeal aspirate taken immediately after hospital admission, with PCR to assess for 9 viruses. Compared by RSV alone versus RSV co-detection with other viruses.  **Comparator**  No allocated comparator (testing was performed on all patients, and comparisons were made by test results). | Prospective observational study, single centre | **ICU admission:** Number (%) of infants admitted to PICU.  RSV vs. RSV co-detection with other viruses:  1/31 (3.2%) vs. 1/31 (3.2%)  **Length of stay:** Mean (SD) length of hospitalisation in days.  RSV (n=31) vs. RSV co-detection with other viruses (n=31):  7.2 (3.6) vs. 7.2 (2.0), *p*=NS.  **Primary outcomes:**   - NR;   Plus other secondary outcomes reported in the article. |  |

## Appendix 5. GRADE certainty of evidence tables

**Chest Xray**

**Author:** PREDICT research network

**Question:** in infants presenting to hospital or hospitalised with bronchiolitis, does performing a CXR at the time of presentation or admission beneficially change medical management or clinically relevant end-points?

**Settings:** ED, hospital ward, ICU

**Bibliography:** Towriss 2025, Williams 2012, Wrotek 2019.

| Quality assessment | | | | | | | No. of patients | Key findings | Quality | Importance |
| --- | --- | --- | --- | --- | --- | --- | --- | --- | --- | --- |
| No. of studies | Design | Risk of bias | Inconsistency | Indirectness | Imprecision | Other considerations |  |  |  |  |
| **Diagnostic accuracy** (various measures; see findings) | | | | | | | | | | |
| 2^1^ | Systematic review (2 observational studies) | Serious^2^ | Not serious | Not serious | Serious^3^ | None | 366 | a) Shaw et al. 1991 (18% ICU): Atelectasis on CXR as a predictor of severe disease, as rated ‘mild’ or ‘severe’ by radiologists: OR 2.7 (95% CI 0.97 to 3.70), Se 21%, Sp 98%, PPV 82%, NPV 70%.  b) Dawson et al. 1990 (unreported proportion in ICU): Association between clinical severity grading and radiological changes of hyperinflation, infiltration, and atelectasis: No significant relationship between clinical severity ratings and the degree of hyperinflation (X^2^=9.92 (df 9), *p*<.10), degree of infiltration (X^2^=4.56 (df 12), *p*<.10), or a summed grading score indicative of the degree of hyperinflation, infiltration, and atelectasis (X^2^=6.55 (df 6), *p*<.10). | ⊕⊝⊝⊝  Very low | Critical |
| **Indicator for administration of antibiotics** (number of infants who received antibiotics) | | | | | | | | | | |
| 1^4^ | Retrospective observational | Very serious^5^ | NA | Serious^6^ | Serious^7^ | None | 431 | **Pathological CXR vs. benign CXR:**  155/248 (62.5%) vs. 73/183 (39.9%)  RR 1.57 (95% CI 1.28 to 1.92)  227 more per 1,000 (from 112 more to 367 more) | ⊕⊝⊝⊝  Very low | Critical |
| 1^8^ | Systematic review (1 observational study) | Serious^2^ | NA | Not serious | Very serious^9^ | None | 265 | **Pre-CXR vs. post-CXR:**  7/265 (2.6%) vs. 39/265 (14.7%)  RR 5.57 (95% CI 2.54 to 12.23)  121 more per 1,000 (from 41 more to 297 more) | ⊕⊝⊝⊝  Very low | Critical |
| 1^10^ | Retrospective observational | Very serious^11^ | NA | Not serious | Very serious^12^ | None | 581 | **CXR vs. no CXR:**  22.9 fold increase in risk of antibiotic treatment post CXR (irrespective of result) (95% CI 14.1 to 37.1), *p*<.01.  **Positive CXR vs. negative CXR:**  4.4 fold (95% CI 2.2 to 8.9) greater risk of antibiotic treatment with positive CXR compared to negative CXR (*p*<.01). | ⊕⊝⊝⊝  Very low | Critical |
| **Cost-effectiveness** (cost savings per patient with omitting CXR (2005 CAD)) | | | | | | | | | | |
| 1^13^ | Systematic review (1 observational study) | Serious^2^ | NA | Serious^14^ | Serious^15^ | None | 265 | Cost saving of 2005 CAD$59.09 per patient with omitting CXR. | ⊕⊝⊝⊝  Very low | Important |
| **Readmission to hospital** | | | | | | | | | | |
| 0 | NA | NA | NA | NA | NA | NA | NA | NA | NA | Important |
| CAD= Canadian dollar; CI= confidence interval; CXR= chest Xray; df= degrees of freedom; ICU= intensive care unit; NA= not applicable; NPV= negative predictive value; OR= odds ratio; PPV= positive predictive value; RR= relative risk; Se= sensitivity; Sp= specificity.  Observational evidence was downgraded to low quality at the outset due to being an observational study where randomised controlled trial evidence were sought.  ^1^ Williams 2012 (Shaw et al. 1991, Dawson et al. 1990).  ^2^ Serious risk of bias. Downgraded as the systematic review evidence was at high risk of bias.  ^3^ Serious imprecision. Downgraded due to the small sample sizes and wide 95% CIs where reported.  ^4^ Towriss 2025.  ^5^ Very serious risk of bias. Downgraded twice due to serious limitations in two domains (selection and comparability of cohorts).  ^6^ Serious indirectness. Downgraded due to indirectness in the comparison (by test results).  ^7^ Serious imprecision. Downgraded due to a small sample size and reasonably wide 95% CI.  ^8^ Williams 2012 (Schuh et al., 2007).  ^9^ Very serious imprecision. Downgraded twice due to a small sample size and very wide 95% CIs.  ^10^ Wrotek 2019.  ^11^ Very serious risk of bias. Downgraded twice due to limitations in all three domains (selection, comparability of cohorts, outcome measurement).  ^12^ Very serious imprecision. Downgraded twice due to very wide 95% CIs.  ^13^ Williams 2012 (Yong et al., 2009).  ^14^ Serious indirectness. Downgraded due to the dated currency estimate.  ^15^ Serious imprecision. Downgraded due to the small sample size, confidence intervals not reported. | | | | | | | | | | |

**Author:** PREDICT research network

**Question:** in infants severely unwell with bronchiolitis (HDU/ ICU level care), does performing a CXR beneficially change medical management or clinically relevant end points?

**Settings:** ICU

**Bibliography:** Akande 2024

| Quality assessment | | | | | | | No. of patients | Key findings | Quality | Importance |
| --- | --- | --- | --- | --- | --- | --- | --- | --- | --- | --- |
| No. of studies | Design | Risk of bias | Inconsistency | Indirectness | Imprecision | Other considerations |  |  |  |  |
| **Diagnostic accuracy** | | | | | | | | | | |
| 0 | NA | NA | NA | NA | NA | NA | NA | NA | NA | Critical |
| **Length of stay** | | | | | | | | | | |
| 0 | NA | NA | NA | NA | NA | NA | NA | NA | NA | Critical |
| **Length of ICU stay** (marginal mean days) | | | | | | | | | | |
| 1^1^ | Retrospective observational | Serious^2^ | NA | Serious^3^ | Serious^4^ | None | 149 | **Presence of normal vs. abnormal chest radiograph findings:**  RR 0.83 (95% CI 0.69 to 1.01), *p*=.07. | ⊕⊝⊝⊝  Very low | Critical |
| **Cost-effectiveness** | | | | | | | | | | |
| 0 | NA | NA | NA | NA | NA | NA | NA | NA | NA | Important |
| **Indicator for administration of antibiotics** | | | | | | | | | | |
| 0 | NA | NA | NA | NA | NA | NA | NA | NA | NA | Important |
| CI= confidence interval; CXR= chest Xray; ICU= intensive care unit; NA= not applicable; RR= relative risk.  Observational evidence was downgraded to low quality at the outset due to being an observational study where randomised controlled trial evidence were sought.  Observational evidence were downgraded to low quality at the outset as randomised controlled trial evidence were sought.  ^1^ Akande 2024.  ^2^ Serious risk of bias. Downgraded due to serious concerns in one domain (selection of cohort).  ^3^ Serious indirectness. Downgraded due to indirectness in the comparison (by test results).  ^4^ Serious imprecision. Downgraded due to the small sample size and reasonably wide 95% CI. | | | | | | | | | | |

**Laboratory testing**

**Author:** PREDICT research network

**Question:** In infants presenting to hospital or hospitalised with bronchiolitis, does performing laboratory tests (blood and/or urine), at the time of presentation or admission, beneficially change medical management or clinically relevant end points?

**Settings:** ED, hospital ward, ICU, outpatient clinic.

**Bibliography:** Al Shibli 2017; Burrack 2023; Cebey-Lopez 2016; Celik 2020; Erdede 2023; Fares 2011; Matera 2022; McDaniel 2019; Mitri 2012; Rodriguez-Gonzalez 2022, 2023; Sun 2020.

| Quality assessment | | | | | | | No. of patients | Key findings | Quality | Importance |
| --- | --- | --- | --- | --- | --- | --- | --- | --- | --- | --- |
| No. of studies | Design | Risk of bias | Inconsistency | Indirectness | Imprecision | Other considerations |  |  |  |  |
| **Length of stay** (mean (SD) length of hospital stay in days) | | | | | | | | | | |
| 1^1^ | Retrospective observational | Very serious^2^ | Not serious | Serious^3^ | Serious^4^ | None | 305 | **Thrombocytosis none vs. mild vs. moderate vs. severe:**  4.0 (5.5) vs. 4.4 (4) vs. 4.5 (3) vs. 5.0 (0), *p*=.9. | ⊕⊝⊝⊝  Very low | Critical |
| 1^5^ | Retrospective cohort | Serious^6^ | NA | Serious^3^ | Serious^7^ | None | 2,038 | **MLR quartiles:**  a) Q1 vs. Q2 vs. Q3 vs. Q4:  3.1 (3.0) vs. 3.2 (2.4) vs. 3.7 (3.2) vs. 4.1 (3.3), *p*<.001.  b) i) Q4 vs. Q1: Adj RR 1.6 (95% CI 1.3 to 2.0), *p*<.001.  ii) Q3 vs. Q1: Adj RR 1.4 (95% CI 1.1 to 1.8), *p*=.003.  iii) Q2 vs. Q1: Adj RR 1.0 (95% CI 0.8 to 1.3), *p*=.9. | ⊕⊝⊝⊝  Very low | Critical |
| 1^5^ | Retrospective cohort | Serious^6^ | NA | Serious^3^ | Serious^7^ | None | 2,038 | **NLR quartiles:**  a) Q1 vs. Q2 vs. Q3 vs. Q4:  3.3 (2.7) vs. 2.2 (3.0) vs. 3.6 (2.8) vs. 3.8 (3.6), *p*=.03.  b) i) Q4 vs. Q1: Adj RR 1.7 (95% CI 1.4 to 2.2), *p*<.001.  ii) Q3 vs. Q1: Adj RR 1.5 (95% CI 1.2 to 1.8), *p*<.001.  iii) Q2 vs. Q1: Adj RR 1.1 (95% CI 0.9 to 1.4), *p*=.5. | ⊕⊝⊝⊝  Very low | Critical |
| 1^8^ | Prospective observational | Very serious^9^ | NA | Serious^3^ | Very serious^10^ | None | 66 | **PCR-confirmed bacteraemia positive vs. negative:**  12.1 (4.3) vs. 7.5 (4.7), *p*=.007. | ⊕⊝⊝⊝  Very low | Critical |
| 1^11^ | Retrospective cohort | Very serious^12^ | NA | Serious^3^ | Very serious^13^ | None | 155 | **Systemic immune-inflammation index:**  r= -0.03, *p*=.66. | ⊕⊝⊝⊝  Very low | Critical |
| 1^14^ | Prospective observational | Very serious^15^ | NA | Serious^3^ | Very serious^16^ | None | 49 | **Positive vs. negative tracheal aspirate culture:**  7.60 (2.38) vs. 4.50 (0.71), *p*>.05. | ⊕⊝⊝⊝  Very low | Critical |
| 1^17^ | Retrospective cohort | Very serious^18^ | NA | Serious^3^ | Not serious | None | 1,297 | **Lymphocyte count:**  G1 vs. G2 vs. G3:  5.8 (3.3) vs. 5.1 (3.3) vs. 5.0 (2.3), *p*<.001. | ⊕⊝⊝⊝  Very low | Critical |
| **Length of stay** (median (IQR) length of hospital stay in days) | | | | | | | | | | |
| 1^19^ | Pilot prospective observational | Serious^20^ | Not serious | Serious^3^ | Very serious^21^ | None | 17 | **Urinary log-10-NT-proBNP/creatinine ratio:**  Spearman’s ρ=0.84, *p*<.001.  4 days (IQR 2 to 11) | ⊕⊝⊝⊝  Very low | Critical |
| 1^22^ | Prospective observational | Very serious^23^ | Not serious | Serious^3^ | Very serious^24^ | None | 149 | **Glycemia:**  Spearman’s ρ=0.252, *p*=.005  **CRP:**  ρ=0.348, *p*=.000  **CRP/Albumin:**  ρ=0.340, *p*=.000  **NT-ProBNP:**  ρ=0.581, *p*=.000  Overall sample: 4 days (IQR 2 to 9) | ⊕⊝⊝⊝  Very low | Critical |
| 1^25^ | Retrospective observational | Very serious^26^ | Not serious | Serious^3^ | Serious^27^ | None | 404 | **Thrombocytosis vs. normal platelet count:**  7.0 (6.0 to 8.0) vs. 7.0 (7.0 to 8.0), *p*=.54. | ⊕⊝⊝⊝  Very low | Critical |
| **Length of stay** (number of infants with a hospital stay <3 days versus ≥3 days) | | | | | | | | | | |
| 1^28^ | Prospective cohort (secondary analysis) | Not serious | NA | Serious^3^ | Serious^29^ | None | 1,016 | **Normal vs. high blood eosinophil values:**  LOS <3 days:  430/713 (60%) vs. 103/160 (64%)  RR 1.08 (95% CI 0.94 to 1.22)  LOS ≥3 days:  283/713 (40%) vs. 57/160 (36%).  RR 0.90 (95% CI 0.72 to 1.13) | ⊕⊝⊝⊝  Very low | Critical |
| **Length of stay** (hospital duration ≥9 days) | | | | | | | | | | |
| 1^30^ | Prospective cohort | Very serious^31^ | NA | Serious^3^ | Very serious^32^ | None | 146 | **NLR cut-off value >1.04:**  AUC 0.675 (95% CI 0.548 – 0.802), Se 44.4%, Sp 81%, *p*=.022. | ⊕⊝⊝⊝  Very low | Critical |
| **Death** (number of deaths) | | | | | | | | | | |
| 1^1^ | Retrospective observational | Very serious^2^ | NA | Serious^3^ | Very serious^33^ | None | 305 | **Thrombocytosis none vs. mild vs. moderate vs. severe:**  0/217 (0%) vs. 0/78 (0%) vs. 0/9 (0%) vs. 0/1 (0%). | ⊕⊝⊝⊝  Very low | Critical |
| 1^19^ | Pilot prospective observational | Serious^20^ | NA | Serious^3^ | Very serious^33^ | None | 17 | **Urinary log-10-NT-proBNP/creatinine ratio:**  0/17 (0%). | ⊕⊝⊝⊝  Very low | Critical |
| 1^22^ | Prospective observational | Very serious^23^ | NA | Serious^3^ | Very serious^33^ | None | 149 | **Glycemia, CRP, CRP/albumin, NT-proBNP:**  0/149 (0%). | ⊕⊝⊝⊝  Very low | Critical |
| **Length of ICU stay** | | | | | | | | | | |
| 0 | NA | NA | NA | NA | NA | NA | NA | NA | NA | Important |
| **Diagnosis of bacterial co-infection** (diagnosis of UTI; range of definitions) | | | | | | | | | | |
| 1^34^ | Systematic review (18 observational studies) | Not serious | Serious^35^ | Serious^36^ | Not serious | None | 7,453 | Definitions of a positive urinalysis test result for diagnosing UTI in infants with bronchiolitis varied between studies.  The most common definition was at least 10,000 cfu/mL of a single pathogen on a catheterized specimen.  Others accepted any growth of a known pathogen as a positive result.  Three studies did not define what a positive UA result entailed. | ⊕⊝⊝⊝  Very low | Important |
| **Diagnosis of bacterial co-infection** (number of infants with a diagnosis of bacteremia, pneumonia, UTI) | | | | | | | | | | |
| 1^5^ | Retrospective cohort | Serious^6^ | NA | Serious^3^ | Not serious | None | 2,038 | **MLR:**  Q1 vs. Q2 vs. Q3 vs. Q4:  i) Bacteremia: 0 (0.0%) vs. 3 (0.6%) vs. 4 (0.8%) vs. 1 (0.2%), *p*=.13.  ii) Pneumonia: 34 (6.7%) vs. 33 (6.5%) vs. 30 (5.9%) vs. 34 (6.7%), *p*>.9.  iii) UTI: 2 (0.4%) vs. 6 (1.2%) vs. 6 (1.2%) vs. 8 (1.6%), *p*=.3. | ⊕⊝⊝⊝  Very low | Important |
| 1^5^ | Retrospective cohort | Serious^6^ | NA | Serious^3^ | Not serious | None | 2,038 | **NLR:**  a) Q1 vs. Q2 vs. Q3 vs. Q4:  i) Bacteremia: 2 (0.4%) vs. 3 (0.6%) vs. 1 (0.2%) vs. 2 (0.4%), *p*>.9.  ii) Pneumonia: 19 (3.7%) vs. 29 (5.7%) vs. 34 (6.7%) vs. 49 (9.6%), *p*=.002.  iii) UTI: 1 (0.2%) vs. 6 (1.2%) vs. 6 (1.2%) vs. 9 (1.8%), *p*=.11. | ⊕⊝⊝⊝  Very low | Important |
| **Diagnosis of bacterial co-infection** (number of infants with definite bacterial co-infection (CRP ≥2 mg/dL)) | | | | | | | | | | |
| 1^14^ | Prospective observational | Very serious^15^ | NA | Serious^3^ | Very serious^37^ | None | 49 | **Positive vs. negative tracheal aspirate culture:**  5/29 (17.2%) vs. 0/20 (0%).  RR 7.70 (95% CI 0.45 to 131.91) | ⊕⊝⊝⊝  Very low | Important |
| Adj RR= adjusted relative risk; AUC= area under the curve; CI= confidence interval; CRP= c-reactive protein; ICU= intensive care unit; LOS= length of stay; MLR= monocyte-to-lymphocyte ratio; NA= not applicable; NLR= neutrophil-to-lymphocyte ratio; NT-proBNP= N-terminal pro-brain natriuretic peptide; RR= relative risk; SD= standard deviation; Se= sensitivity; Sp= specificity; UTI= urinary tract infection.  Observational evidence were downgraded to low quality at the outset as randomised controlled trial evidence were sought.  ^1^ Al Shibli 2017.  ^2^ Very serious risk of bias. Downgraded twice due to concerns in all three domains (selection, comparability of cohorts, outcome measurement).  ^3^ Serious indirectness. Downgraded due to indirectness in the comparison (by test results).  ^4^ Serious imprecision. Downgraded due to a small sample size, lack of CIs.  ^5^ Burrack 2023.  ^6^ Serious risk of bias. Downgraded due to serious concerns in one domain (selection of cohort).  ^7^ Serious imprecision. Downgraded due to reasonably wide CIs.  ^8^ Cebey-Lopez 2016.  ^9^ Very serious risk of bias. Downgraded twice due to serious concerns in two domains (selection, comparability of cohorts).  ^10^ Very serious imprecision. Downgraded twice due to the very small sample size.  ^11^ Erdede 2023.  ^12^ Very serious risk of bias. Downgraded twice due to serious concerns in two domains (selection, comparability of cohorts).  ^13^ Very serious imprecision. Downgraded twice due to the very small sample size. Means (SD) and CIs not reported.  ^14^ Fares 2011.  ^15^ Very serious risk of bias. Downgraded twice due to serious concerns in two domains (selection, comparability of cohorts).  ^16^ Very serious imprecision. Downgraded twice due to the very small sample size.  ^17^ Matera 2022.  ^18^ Very serious risk of bias. Downgraded twice due to concerns in two domains (selection, comparability of cohorts).  ^19^ Rodriguez-Gonzalez 2023.  ^20^ Serious risk of bias. Downgraded due to serious concerns in one domain (selection of cohort).  ^21^ Very serious imprecision. Downgraded twice due to the extremely small sample size (pilot study of 17 participants).  ^22^ Rodriguez-Gonzalez 2022.  ^23^ Very serious risk of bias. Downgraded twice due to concerns in two domains (selection, comparability of cohorts).  ^24^ Very serious imprecision. Downgraded twice due to the very small sample size, CIs not reported.  ^25^ Sun 2020.  ^26^ Very serious risk of bias. Downgraded twice due to serious concerns in two domains (selection, comparability of cohorts).  ^27^ Serious imprecision. Downgraded due to the small sample size.  ^28^ Mitri 2012.  ^29^ Serious imprecision. Downgraded due to reasonably wide 95% CIs.  ^30^ Celik 2020.  ^31^ Very serious risk of bias. Downgraded twice due to concerns in all three domains (selection, comparability of cohorts, outcome measurement).  ^32^ Very serious imprecision. Downgraded twice due to the small sample size and very wide 95% CIs that affect the interpretation of the value.  ^33^ Very serious imprecision. Downgraded twice due to a rare event and very small sample size.  ^34^ McDaniel 2019.  ^35^ Serious inconsistency. Downgraded due to some inconsistency in how UTIs were diagnosed between studies.  ^36^ Serious indirectness. Downgraded due to indirectness in the outcome measurement; no statistical evaluation for sensitivity, specificity.  ^37^ Very serious imprecision. Downgraded twice due to the very small sample size and extremely wide CIs. | | | | | | | | | | |

**Author:** PREDICT research network **Question:** In infants severely unwell with bronchiolitis (HDU/ICU level care), does performing laboratory tests (blood and/or urine) beneficially change medical management or clinically relevant end-points? **Settings:** ICU **Bibliography:** Akande 2024; Alejandre 2021; Burrack 2023; Cebey-Lopez 2016; Gaurav 2024; Laham 2014; Rodriguez-Gonzalez 2022; Sun 2020.

| Quality assessment | | | | | | | No. of patients | Key findings | Quality | Importance |
| --- | --- | --- | --- | --- | --- | --- | --- | --- | --- | --- |
| No. of studies | Design | Risk of bias | Inconsistency | Indirectness | Imprecision | Other considerations |  |  |  |  |
| **Length of stay** | | | | | | | | | | |
| 0 | NA | NA | NA | NA | NA | NA | NA | NA | NA | Critical |
| **Death** | | | | | | | | | | |
| 1^1^ | Prospective observational | Serious^2^ | NA | Serious^3^ | Very serious^4^ | None | 60 | **Hyponatremia vs. normonatremia:**  0/21 (0.0%) vs. 0/39 (0.0%). | ⊕⊝⊝⊝  Very low | Critical |
| 1^5^ | Prospective observational | Very serious^6^ | NA | Serious^3^ | Very serious^7^ | None | 37 | **Glycemia, CRP, CRP/albumin, NT-proBNP:**  0/37 (0%) | ⊕⊝⊝⊝  Very low | Critical |
| **Length of ICU stay** (mean (SD) length of ICU stay in days) | | | | | | | | | | |
| 1^8^ | Retrospective cohort | Very serious^9^ | NA | Serious^3^ | Very serious^10^ | None | 104 | **MLR:**  Q1 vs. Q2 vs. Q3 vs. Q4:  4.9 (SD 5.9) vs. 3.8 (SD 1.8) vs. 4.8 (SD 4.2) vs. 5.5 (SD 4.8), *p*=.6. | ⊕⊝⊝⊝  Very low | Important |
| 1^8^ | Retrospective cohort | Very serious^9^ | NA | Serious^3^ | Very serious^10^ | None | 104 | **NLR:**  Q1 vs. Q2 vs. Q3 vs. Q4:  5.3 (SD 5.0) vs. 4.0 (SD 3.3) vs. 4.6 (SD 2.7) vs. 5.7 (SD 5.7), *p*=.8. | ⊕⊝⊝⊝  Very low | Important |
| **Length of ICU stay** (median length of ICU stay in days (IQR)) | | | | | | | | | | |
| 1^11^ | Retrospective observational | Very serious^12^ | NA | Serious^3^ | Very serious^4^ | None | 38/404 | **Thrombocytosis vs. normal platelet count:**  6.0 days (4.5 to 10.5) vs. 5.50 days (4.0 to 9.0), *p*=.553. | ⊕⊝⊝⊝  Very low | Important |
| 1^13^ | Retrospective cohort | Serious^14^ | NA | Serious^3^ | Serious^15^ | None | 148 | **Codetection vs. no codetection in lower respiratory cultures:**  6.9 days (95% CI 6.21-7.68) vs. 8.57 days (7.68–9.56)  Adj RR 0.81 (95% CI 0.69–0.94), *p*<.01. | ⊕⊝⊝⊝  Very low | Important |
| 1^1^ | Prospective cohort | Serious^2^ | NA | Serious^3^ | Very serious^4^ | None | 60 | **Hyponatremia vs. normonatremia:**  <2 days: 13 vs. 25  2-4 days: 7 vs. 12  >4 days: 1 vs. 2 | ⊕⊝⊝⊝  Very low | Important |
| 1^16^ | Prospective observational | Very serious^17^ | NA | Serious^3^ | Very serious^18^ | None | 149 | **Glycemia:**  Spearman’s ρ=0.256, *p*=.15  **CRP:**  ρ=0.043, *p*=.81  **CRP/Albumin:**  ρ=-0.07, *p*=.99  **NT-ProBNP:**  ρ=0.490, *p*=.02 | ⊕⊝⊝⊝  Very low | Important |
| **Diagnosis of bacterial co-infection** (performance at predicting bacterial co-infection via AUC (95% CI), and cut-off values with sensitivity and specificity) | | | | | | | | | | |
| 1^19^ | Prospective observational | Not serious | NA | Not serious | Serious^20^ | None | 675 | **PCT:**  Best cut-off points for invasive bacterial infection (IBI) diagnosis:  a) At admission:  1.4 ng/mL, Sn 69% (95% CI 58.4 to 74.9), Sp 91% (95% CI 88.1 to 92.5), positive predictive value 76.7% (95% CI 70.2 to 83.1), negative predictive value 86.2% (95% CI 83.1 to 89.3).  b) 24 hours after admission:  3.3 ng/mL, Sn 56% (95% CI 48.5 to 64.5), Sp 90.7% (95% CI 84.9 to 93.6%), positive predictive value 81.9% (95% CI 72.1 to 87.2), negative predictive value 74.3% (95% CI 68.2 to 79.1).  **CRP:**  Best cut-off points for IBI diagnosis:  a) At admission:  26 mg/dL, Sn 62.7%, Sp 71.9% (95% CIs, PPV, NPV not reported)  b) 24 hours after admission:  38 mg/dL, Sn 72.9%, Sp 64.1% (95% CIs, PPV, NPV not reported)  **PCT and CRP:**  Area under the curve (AUC) (95% CI) for diagnosing IBI according to the values of PCT and CRP at admission, 24h, and 48h.  Admission:  PCT: 0.835 (0.792 to 0.878)  CRP: 0.716 (0.66 to 0.76)  After 24 hours:  PCT: 0.833 (0.765 to 0.902)  CRP: 0.718 (0.638 to 0.798)  After 48 hours:  PCT: 0.670 (0.570 to 0.771)  CRP: 0.630 (0.526 to 0.733)  AUC (95% CI) for diagnosing sepsis.  At admission:  PCT: 0.91 (0.87 to 0.95)  CRP: 0.73 (0.66 to 0.8)  After 24 hours:  PCT: 0.89 (0.92 to 0.95)  CRP: 0.73 (0.64 to 0.82)  After 48 hours:  PCT: 0.81 (0.71 to 0.91)  CRP: 0.64 (0.50 to 0.78)  AUC (95% CI) for diagnosing pneumonia.  At admission:  PCT: 0.82 (0.77 to 0.87)  CRP: 0.77 (0.72 to 0.83)  After 24 hours:  PCT: 0.81 (0.74 to 0.88)  CRP: 0.71 (0.62 to 0.8)  After 48 hours:  PCT: 0.64 (0.52 to 0.75)  CRP: 0.65 (0.53 to 0.76)  AUC (95% CI) for diagnosing UTI not reported. | ⊕⊝⊝⊝  Very low | Important |
| **Diagnosis of bacterial co-infection** (number of patients with a positive PCR and blood bacterial PCR) | | | | | | | | | | |
| 1^21^ | Prospective observational | Serious^22^ | NA | Serious^3^ | Very serious^4^ | None | 28 | **Number of PICU patients with positive PCR:**  7 of 28 (25%)  **Number of patients with a positive blood bacterial PCR:**  1 of 7 (14.3%) | ⊕⊝⊝⊝  Very low | Important |
| **Diagnosis of bacterial co-infection** (various measures) | | | | | | | | | | |
| 1^23^ | Retrospective cohort | Very serious^24^ | NA | Not serious | Very serious^4^ | None | 40 | **Bacterial cultures:**  Number of patients diagnosed with bacterial co-infection from bacterial cultures.  Any co-infection: 9 of 40 (22.5%).  Bacterial pneumonia: 8 of 18 (44.4%) tested.  Urinary tract infection: 2 of 33 (6.1%) tested.  Bacteraemia: 0 of 36 (0.0%) tested.  Meningitis: 0 of 12 (0.0%) tested.  **PCT vs. WBC:**  Performance of PCT versus WBC at predicting a diagnosis of bacterial co-infection.  PCT (>1.5 ng/mL cut-off value):  AUC 0.88, Se 0.80, Sp 1.00. Serum PCT was significantly associated with bacterial co-infection (*p*<.0001).  WBC (<6400/µL cut-off value):  AUC 0.67, Se 0.33, Sp 0.96, *p*=.06. | ⊕⊝⊝⊝  Very low | Important |
| Adj RR= adjusted relative risk; AUC= area under the operating curve; CI= confidence interval; CRP= C-reactive protein; IBI= invasive bacterial infection; ICU= intensive care unit; IQR= interquartile range; MLR= monocyte-to-lymphocyte ratio; NA= not applicable; NLR= neutrophil-to-lymphocyte ratio; NT-proBNP= N-terminal pro-brain natriuretic peptide; PCR= polymerase chain reaction; PCT= serum procalcitonin; PICU= paediatric intensive care unit; Se= sensitivity; Sp= specificity; UTI= urinary tract infection; WBC= white blood cell count.  Observational evidence was downgraded to low quality at the outset due to being observational evidence in a question where RCT evidence was sought.  ^1^ Gaurav 2024.  ^2^ Serious risk of bias. Downgraded due to serious concerns about confounding, selection of participants, and selection of the reported result, and insufficient information about missing data.  ^3^ Serious indirectness. Downgraded due to indirectness in the comparison (by test result).  ^4^ Very serious imprecision. Downgraded due to the very small sample size relative to an adequately powered trial.  ^5^ Rodriguez-Gonzalez 2022.  ^6^ Very serious risk of bias. Downgraded twice due to serious concerns in two domains (selection, comparability of cohorts).  ^7^ Very serious imprecision. Downgraded twice due to the very small sample size, CIs not reported.  ^8^ Burrack 2023.  ^9^ Very serious risk of bias. Downgraded twice as the evidence was rated at critical risk of bias due to critical concerns about confounding, moderate concerns about selection of participants and the reported result, and insufficient information to assess bias from missing data.  ^10^ Very serious imprecision. Downgraded due to a very small sample size and reasonably large IQRs. 95% CJs not reported.  ^11^ Sun 2020.  ^12^ Very serious risk of bias. Downgraded twice as the evidence was rated at high risk of bias due to serious concerns about confounding and participant selection, moderate concerns about selection of the reported result, and insufficient information reported to assess bias from missing data.  ^13^ Akande 2024.  ^14^ Serious risk of bias. Downgraded due to serious concerns about confounding, insufficient information about missing data, and moderate concerns about selection of the reported result.  ^15^ Serious imprecision. Downgraded due to the small sample size and reasonably wide 95% CIs.  ^16^ Rodriguez-Gonzalez 2022.  ^17^ Very serious risk of bias. Downgraded twice due to serious concerns in two domains (selection, comparability of cohorts).  ^18^ Very serious imprecision. Downgraded twice due to the very small sample size, CIs not reported.  ^19^ Alejandre 2021.  ^20^ Serious imprecision. Downgraded as 95% CIs for AUC values crossed the threshold for good predictive performance in multiple instances.  ^21^ Cebey-Lopez 2016.  ^22^ Serious risk of bias. Downgreded as the evidence is at unclear risk of bias, where the limitations were sufficient to lower confidence in the estimates of effect.  ^23^ Laham 2014.  ^24^ Very serious risk of bias. Downgraded twice due to critical concerns about confounding, serious concerns about the selection of participants, moderate concerns about selection of the reported result, and insufficient information about missing data. | | | | | | | | | | |

**Viral testing**

**Author:** PREDICT research network

**Question:** In infants presenting to hospital or hospitalised with bronchiolitis, does performing virological investigations beneficially change medical management or clinically relevant end-points?

**Settings:** ED, hospital ward, ICU, outpatient clinic.

**Bibliography:** Akande 2024; Ambrożej 2024; Bamberger 2012; Bermúdez-Barrezueta 2023; Boggio 2023; Celik 2020; Coleman 2019; Erdede 2023; Huguenin 2012; Mansbach 2012; Petrarca 2018; Sai Kotha 2023; Tan 2021; Yu 2010.

| Quality assessment | | | | | | | No. of patients | Key findings | Quality | Importance |
| --- | --- | --- | --- | --- | --- | --- | --- | --- | --- | --- |
| No. of studies | Design | Risk of bias | Inconsistency | Indirectness | Imprecision | Other considerations |  |  |  |  |
| **Rate of hospitalisation** (number of infants hospitalised) | | | | | | | | | | |
| 3^1^ | Systematic review (3 observational studies) | Serious^2^ | Not serious | Serious^3^ | Serious^4^ | None | 232 | **RSV-bronchiolitis vs. RV-bronchiolitis:**  1) Nascimento 2010:  2 of 13 (15%) vs. 19 of 36 (53%), *p* not reported.  2) Luchsinger 2014:  10 of 22 (46%) vs. 57 of 74 (77%), ***p*<.05.**  3) Diaz 2015:  1 of 27 (4%) vs. 49 of 60 (82%), *p* not reported. | ⊕⊝⊝⊝  Very low | Critical |
| **Death** | | | | | | | | | | |
| 0 | NA | NA | NA | NA | NA | NA | NA | NA | NA | Critical |
| **Rate of ICU admission** (OR for PICU transfer) | | | | | | | | | | |
| 5^1^ | Systematic review (5 observational studies) | Serious^5^ | Serious^6^ | Serious^3^ | Serious^7^ | None | 1,010 | **RSV-bronchiolitis vs. RV-bronchiolitis**:  130/ 818 (15.9%) vs. 28/ 192 (14.6%)  OR 0.87 (95% CI 0.01 to 3.97), *p*=.76. | ⊕⊝⊝⊝  Very low | Critical |
| **Rate of ICU admission** (number of infants admitted to ICU) | | | | | | | | | | |
| 1^8^ | Retrospective cohort | Serious^9^ | NA | Serious^3^ | Very serious^10^ | None | 445 | **Single virus vs. co-infections:**  61 of 270 (22.6%) vs. 44 of 175 (25.1), *p*=.54.  **No virus (reference) vs. 2 viruses:**  Adj HR 0.94 (95% CI 0.53 to 1.67), *p*=.56.  **No virus (reference) vs. ≥3 viruses:**  Adj HR 1.84 (95% CI 0.88 to 3.86), *p*=.07. | ⊕⊝⊝⊝  Very low | Critical |
| 1^11^ | Prospective observational | Serious^12^ | NA | Serious^3^ | Very serious^10^ | None | 126 | **Single vs. multiple viral infections:**  5/41 (12.2%) vs. 5/85 (5.9%), *p*=.29.  RR 2.10 (95% CI 0.55 to 7.97) | ⊕⊝⊝⊝  Very low | Critical |
| 1^13^ | Prospective observational | Serious^14^ | NA | Serious^3^ | Serious^14^ | None | 1,866 | **RSV only vs. HRV only vs. RSV plus HRV, vs. RSV plus non-HRV vs. HRV plus non-RSV:**  17.2% vs. 15.8% vs. 18.1% vs. 17.1% vs. 17.6%, *p*=.98. | ⊕⊝⊝⊝  Very low | Critical |
| 1^15^ | Prospective observational | Very serious^16^ | NA | Serious^3^ | Very serious^10^ | None | 486 | **Single infection vs. co-infection:**  48/ 431 (11.2%) vs. 13/ 55 (23.6%), *p*=NS.  RR 0.47 (95% CI 0.27 to 0.81)  **RSV vs. RSV co-infection:**  38/ 320 (11.9%) vs. 6/ 45 (13.3%), *p*=NS.  RR 0.89 (95% CI 0.40 to 1.99)  **hRV vs. hRV co-infection:**  8/ 60 (13.6%) vs. 2/ 29 (6.9%), *p*=NS.  RR 1.93 (95% CI 0.44 to 8.53)  **RSV vs. hRV vs. hRV + RSV:**  38/ 320 (11.9%) vs. 8/ 60 (13.6%) vs. 2/ 20 (9.5%), *p*=NS. | ⊕⊝⊝⊝  Very low | Critical |
| 1^17^ | Retrospective observational | Very serious^18^ | NA | Serious^3^ | Serious^19^ | None | 842 | **Single virus vs. mixed virus:**  65/614 (10.6%) vs. 7/70 (10%)  RR 1.06 (95% CI 0.51 to 2.22) | ⊕⊝⊝⊝  Very low | Critical |
| 1^20^ | Prospective observational | Very serious^18^ | NA | Serious^3^ | Very serious^21^ | None | 112 | **RSV vs. RSV co-detection with other viruses:**  1/31 (3.2%) vs. 1/31 (3.2%)  RR 1.00 (95% CI 0.07 to 15.28) | ⊕⊝⊝⊝  Very low | Critical |
| **Length of stay** (varying measures) | | | | | | | | | | |
| 5^1^ | Systematic review (5 observational studies) | Serious^5^ | Serious^22^ | Serious^3^ | Serious^23^ | None | 2,729 | **RSV-bronchiolitis vs. RV-bronchiolitis:**  1) Marguet 2009:  Median 3 days (IQR 3 to 4.7) vs. 6 days (IQR 5 to 8), ***p*<.05**.  2) Mansbach 2016:  Mean 2.6 days (SD 3.5) vs. 3.3 days (3.4), *p* not reported.  Median 2 days (IQR 1 to 3) vs. 2 days (IQR 1 to 4), *p* not reported.  3) Janahi 2017 (n of infants with hospitalisation length >4 days):  52 of 58 (90%) vs. 133 of 160 (83%), *p* not reported.  4) Hasegawa 2019:  Mean 2.9 days (SD 5.8) vs. 3.0 days (SD 3.7), *p* not reported.  Median 2 days (IQR 1 to 2) vs. 2 days (IQR 1 to 3), *p* not reported.  5) Arroyo 2020:  Mean 6.47 days (SD 11.01) vs. 12.91 days (SD 20.41), *p* not reported. | ⊕⊝⊝⊝  Very low | Important |
| **Length of stay** (number of infants with ≤3 days, 4-7 days, >7 days of hospitalisation) | | | | | | | | | | |
| 1^24^ | Prospective observational | Very serious^18^ | NA | Serious^3^ | Serious^4^ | None | 307 | ≤3 days:  Only RSV: 62 (41.6%)  Also RSV: 47 (52.2%)  No RSV: 28 (65.1%)  No pathogen: 11 (44%)  4-7 days:  Only RSV: 76 (51%)  Also RSV: 35 (38.9%)  No RSV: 13 (38.9%)  No pathogen: 11 (44%)  >7 days:  Only RSV: 11 (7.4%)  Also RSV: 8 (8.9%)  No RSV: 2 (4.7%)  No pathogen: 3 (12%)  *p*=.01.  (Post hoc tests, only RSV vs. no RSV *p*<.02). | ⊕⊝⊝⊝  Very low | Important |
| **Length of stay** (median (IQR) length of hospital stay in days) | | | | | | | | | | |
| 1^8^ | Retrospective cohort | Serious^9^ | NA | Serious^3^ | Serious^25^ | None | 455 | **Single virus vs. co-infections:**  6 (4 to 8) vs. 7 (5 to 10), *p*=.004.  **No viruses (reference) vs. 2 viruses:**  Adj HR 0.89 (95% CI 0.69 to 1.07), *p*=.18.  **No viruses (reference) vs. ≥3 viruses:**  Adj HR 0.57 (95% CI 0.41 to 0.79), *p*<.001. | ⊕⊝⊝⊝  Very low | Important |
| 1^26^ | Retrospective observational | Very serious^18^ | NA | Serious^3^ | Serious^27^ | None | 306 | **1 virus vs. 2+ viruses:**  4 (3 to 6) vs. 5 (4 to 9), *p*=.03. | ⊕⊝⊝⊝  Very low | Important |
| 1^15^ | Prospective observational | Very serious^16^ | NA | Serious^3^ | Very serious^28^ | None | 486 | **Single infection vs. co-infection:**  5 (1-45) vs. 4 (2-12), *p*=NS.  **RSV vs. RSV co-infection:**  5 (1-27) vs. 5 (2-12), *p*=NS.  **hRV vs. hRV co-infection:**  5 (2-16) vs. 5 (2-9), *p*=NS.  **RSV vs. hRV vs. hRV + RSV):**  5 (1-27) vs. 5 (2-16) vs. 5 (3-9), *p*=NS. | ⊕⊝⊝⊝  Very low | Important |
| 1^17^ | Retrospective observational | Very serious^18^ | NA | Serious^3^ | Serious^29^ | None | 842 | **Single virus vs. mixed virus:**  8 days vs. 8 days | ⊕⊝⊝⊝  Very low | Important |
| **Length of stay** (mean (SD) duration of hospital stay in days) | | | | | | | | | | |
| 1^30^ | Prospective observational | Very serious^16^ | NA | Serious^3^ | Very serious^31^ | None | 146 | **RSV vs. RV:**  6.39 (3.70) vs. 5.82 (3.48), *p*=.45. | ⊕⊝⊝⊝  Very low | Important |
| 1^32^ | Retrospective observational | Very serious^18^ | NA | Serious^3^ | Very serious^31^ | None | 155 | **RSV alone vs. non-RSV alone:**  6.16 (2.31) vs. 5.49 (2.16), *p*=.14.  **RSV alone vs. RSV co-infection:**  6.16 (2.31) vs. 6.85 (2.37), *p*=.13. | ⊕⊝⊝⊝  Very low | Important |
| 1^11^ | Prospective observational | Serious^12^ | NA | Serious^3^ | Very serious^33^ | None | 126 | **Single vs. multiple viral infections:**  6 (4.5) vs. 5 (3.3), *p*=.52.  Reports a significantly higher length of stay (1.7 (SD 0.6) days, *p*=.04) for infants infected with RSV-A/B in age-adjusted multivariable analyses; RSV negative comparison group assumed. | ⊕⊝⊝⊝  Very low | Critical |
| 1^34^ | Prospective observational | Very serious^18^ | NA | Serious^3^ | Very serious^31^ | None | 90 | **RSV vs. non-RSV:**  4.44 (1.83) vs. 4.27 (1.74), *p*=.64. | ⊕⊝⊝⊝  Very low | Critical |
| 1^20^ | Prospective observational | Very serious^18^ | NA | Serious^3^ | Very serious^31^ | None | 112 | **RSV vs. RSV co-detection with other viruses:**  7.2 (3.6) vs. 7.2 (2.0), *p*=NS. | ⊕⊝⊝⊝  Very low | Critical |
| **Length of stay** (Adj OR for hospital length of stay ≥3 days) | | | | | | | | | | |
| 1^13^ | Prospective observational | Serious^12^ | NA | Serious^3^ | Serious^14^ | None | 1,866 | **RSV only (reference) vs. HRV only:**  Adj OR 0.36 (95% CI 0.20 to 0.63), *p*<.001.  **RSV only (reference) vs. RSV plus HRV:**  Adj OR 1.33 (95% CI 1.02 to 1.73), *p*=.04.  **RSV only vs. RSV plus any other non-HRV pathogen:**  Adj OR 1.06 (95% CI 0.67 to 1.69), *p*=.79.  **RSV only vs. HRV plus any other non-RSV pathogen:**  Adj OR 0.39 (95% CI 0.23 to 0.66), *p*<.001. | ⊕⊝⊝⊝  Very low | Critical |
| **Length of ICU stay** (median length of ICU stay in days) | | | | | | | | | | |
| 1^26^ | Retrospective observational | Very serious^18^ | NA | Serious^3^ | Serious^35^ | None | N=306 | **1 virus vs. 2+ viruses:**  2.1 days (IQR 1 to 3) vs. 3.0 days (IQR 1 to 4), *p*=.06. | ⊕⊝⊝⊝  Very low | Important |
| 1^8^ | Retrospective cohort | Very serious^9^ | NA | Serious^3^ | Serious^35^ | None | 445 | **Single virus vs. co-infections:**  Median 4 days (IQR 3 to 6) vs. 5 days (IQR 3 to 7), *p*=.08. | ⊕⊝⊝⊝  Very low | Important |
| **Length of ICU stay** (proportion of infants per group with the specified number of days in PICU) | | | | | | | | | | |
| 1^24^ | Prospective observational | Very serious^18^ | NA | Serious^3^ | Serious^4^ | None | 311 | 0 days in PICU:  Only RSV: 144/149 (96.6%)  Also RSV: 88/92 (95.7%)  No RSV: 41/44 (93.2%)  No pathogen: 23/26 (88.5%)  1 day in PICU:  Only RSV: 0/149 (0%)  Also RSV: 1/92 (1.1%)  No RSV: 1/44 (2.3%)  No pathogen: 1/26 (3.9%)  >1 day in PICU:  Only RSV: 5/149 (3.4%)  Also RSV: 3/92 (3.3%)  No RSV: 2/44 (4.6%)  No pathogen: 2/26 (7.7%)  *p*=.437. | ⊕⊝⊝⊝  Very low | Important |
| **Length of ICU stay** (mean length of ICU stay in days) | | | | | | | | | | |
| 1^36^ | Prospective cross-sectional | Very serious^18^ | NA | Serious^3^ | Very serious^37^ | None | 70 | **RSV vs. HBoV1:**  13.11 days (SD 8.19) vs. 14.6 days (SD 7.92), *p*>.05. | ⊕⊝⊝⊝  Very low | Important |
| **Length of ICU stay** (marginal mean ICU length of stay in days) | | | | | | | | | | |
| 1^38^ | Retrospective cohort | Serious^9^ | NA | Serious^3^ | Serious^17^ | None | 168 | **RSV positive only vs. other viral categories:**  RR 1.35 (95% CI 1.15-1.60), *p*<.001.  Adj RR 1.40 (95% CI 1.20-1.63), *p*<.0001. | ⊕⊝⊝⊝  Very low | Important |
| Adj HR= adjusted hazard ratio; Adj OR= adjusted odds ratio; Adj RR= adjusted relative risk; CI= confidence interval; HboV1= human bocavirus; HRV= human rhinovirus; ICU= intensive care unit; IQR= interquartile range; NA= not applicable; NA= not significant; OR= odds ratio; PICU= paediatric intensive care unit; RR= relative risk; RSV= respiratory syncytial virus; RV= rhinovirus; SD= standard deviation.  Observational evidence was downgraded to low quality at the outset due to being an observational study where randomised controlled trial evidence was sought.  ^1^ Ambrożej 2024.  ^2^ Serious risk of bias. Downgraded due to moderate risk of bias in one of the three included studies that was sufficient to lower confidence.  ^3^ Serious indirectness. Downgraded due to indirectness in the comparison (by test result).  ^4^ Serious imprecision. Downgraded due to the small sample sizes. 95% CIs not reported.  ^5^ Serious risk of bias. Downgraded due to moderate risk of bias in several of the included studies.  ^6^ Serious inconsistency. Downgraded due to moderate heterogeneity (I^2^=42%).  ^7^ Serious imprecision. Downgraded due to wide 95% CIs that include reasonable benefit and harm.  ^8^ Bermúdez-Barrezueta 2023.  ^9^ Serious risk of bias. Downgraded due to serious concerns in one domain (selection of cohort).  ^10^ Very serious imprecision. Downgraded twice due to the relatively small sample size and very wide 95% CIs.  ^11^ Huguenin 2012.  ^12^ Serious risk of bias. Downgraded due to serious concerns in one domain (selection of cohort).  ^13^ Mansbach 2012.  ^14^ Serious imprecision. Downgraded due to relatively small sizes of all groups aside from RSV (n=1,075). 95% CIs not available.  ^15^ Petrarca 2018.  ^16^ Very serious risk of bias. Downgraded due to concerns in all three domains (selection, comparability, outcome measurement).  ^17^ Tan 2021.  ^18^ Very serious risk of bias. Downgraded twice due to serious concerns in two domains (selection, comparability of cohorts).  ^19^ Serious imprecision. Downgraded due to the small sample size and reasonably wide 95% Cis.  ^20^ Yu 2010.  ^21^ Very serious imprecision. Downgraded twice due to the very small sample size and extremely wide 95% Cis.  ^22^ Serious inconsistency. Downgraded due to reasonable variance in point estimates across studies.  ^23^ Serious imprecision. 95% CIs not reported, very large SDs for effect estimates in one study.  ^24^ Bamberger 2012.  ^25^ Very serious imprecision. Downgraded twice due to the small sample size and reasonably wide 95% CI.  ^26^ Coleman 2019.  ^27^ Serious imprecision. Downgraded due to a small sample size and reasonably wide IQRs.  ^28^ Very serious imprecision. Downgraded twice due to the reasonably small sample size, and very wide IQRs.  ^29^ Serious imprecision. Downgraded due to the reasonably small sample size. 95% Cis, IQRs not reported.  ^30^ Celik 2020.  ^31^ Very serious imprecision. Downgraded twice due to the very small sample size.  ^32^ Erdede 2023.  ^33^ Very serious imprecision. Downgraded twice due to the very small sample size. Reasonably large SDs, 95% Cis not reported.  ^34^ Sai Kotha 2023.  ^35^ Serious imprecision. Downgraded due to the relatively small sample size. 95% CIs not provided.  ^36^ Boggio 2023.  ^37^ Very serious imprecision. Downgraded due to a very small sample size and large SDs for effect estimates. 95% CIs not reported.  ^38^ Akande 2024.  ^39^ Serious imprecision. Downgraded due to the small sample size and reasonably wide 95% CIs. | | | | | | | | | | |
